# Plasma p-tau217 and tau-PET predict future cognitive decline among cognitively unimpaired individuals: implications for clinical trials

**DOI:** 10.1038/s43587-025-00835-z

**Published:** 2025-03-28

**Authors:** Rik Ossenkoppele, Gemma Salvadó, Shorena Janelidze, Alexa Pichet Binette, Divya Bali, Linda Karlsson, Sebastian Palmqvist, Niklas Mattsson-Carlgren, Erik Stomrud, Joseph Therriault, Nesrine Rahmouni, Pedro Rosa-Neto, Emma M. Coomans, Elsmarieke van de Giessen, Wiesje M. van der Flier, Charlotte E. Teunissen, Erin M. Jonaitis, Sterling C. Johnson, Sylvia Villeneuve, PREVENT-AD Research Group, Sylvia Villeneuve, Tammie L. S. Benzinger, Suzanne E. Schindler, Randall J. Bateman, James D. Doecke, Vincent Doré, Azadeh Feizpour, Colin L. Masters, Christopher Rowe, Heather J. Wiste, Ronald C. Petersen, Clifford R. Jack, Oskar Hansson

**Affiliations:** 1 Clinical Memory Research Unit, Department of Clinical Sciences in Malmö, Lund University, Lund, Sweden; 2 Alzheimer Center Amsterdam, Neurology, Vrije Universiteit Amsterdam, Amsterdam, the Netherlands; 3 Neurodegeneration, Amsterdam Neuroscience, Amsterdam, the Netherlands; 4 Memory Clinic, Skåne University Hospital, Malmö, Sweden; 5 Department of Neurology, Skåne University Hospital, Lund University, Lund, Sweden; 6 Wallenberg Center for Molecular Medicine, Lund University, Lund, Sweden; 7 Translational Neuroimaging Laboratory, McGill Research Centre for Studies in Aging, Montreal, Quebec Canada; 8 Department of Neurology and Neurosurgery, Faculty of Medicine, McGill University, Montreal, Quebec Canada; 9 Department of Radiology and Nuclear Medicine, Vrije Universiteit Amsterdam, Amsterdam, the Netherlands; 10 Department of Epidemiology and Biostatistics, Vrije Universiteit Amsterdam, Amsterdam, the Netherlands; 11 Neurochemistry Laboratory, Department of Laboratory Medicine, Vrije Universiteit Amsterdam, Amsterdam, the Netherlands; 12 Wisconsin Alzheimer’s Institute, School of Medicine and Public Health, University of Wisconsin–Madison, Madison, WI USA; 13 Wisconsin Alzheimer’s Disease Research Center, School of Medicine and Public Health, University of Wisconsin–Madison, Madison, WI USA; 14 Centre for Studies on the Prevention of Alzheimer’s Disease (StoP-AD), Douglas Mental Health University Institute, Montreal, Quebec Canada; 15 Department of Psychiatry, McGill University, Montreal, Quebec Canada; 16 Department of Radiology, Washington University School of Medicine, St. Louis, MO USA; 17 Charles F. and Joanne Knight Alzheimer Disease Research Center, Washington, Washington University School of Medicine, St. Louis, MO USA; 18 Department of Neurology, Washington University School of Medicine, St. Louis, MO USA; 19 The Tracy Family SILQ Center, Washington University School of Medicine, St. Louis, MO USA; 20 Australian eHealth Research Centre, Commonwealth Scientific and Industrial Research Organization, Melbourne, Victoria Australia; 21 Department of Molecular Imaging and Therapy, Austin Health, Heidelberg, Victoria Australia; 22 The Florey Institute of Neuroscience and Mental Health, the University of Melbourne, Parkville, Victoria Australia; 23 Department of Quantitative Health Sciences, Mayo Clinic, Rochester, MN USA; 24 Department of Neurology, Mayo Clinic, Rochester, MN USA; 25 Department of Radiology, Mayo Clinic, Rochester, MN USA

**Keywords:** Predictive markers, Alzheimer's disease, Ageing

## Abstract

Plasma p-tau217 and tau positron emission tomography (PET) are strong prognostic biomarkers in Alzheimer’s disease (AD), but their relative performance in predicting future cognitive decline among cognitively unimpaired (CU) individuals is unclear. In a head-to-head comparison study including nine cohorts and 1,474 individuals, we show that plasma p-tau217 and medial temporal lobe tau-PET signal display similar associations with cognitive decline on a global cognitive composite test (*R*^2^_PET_ = 0.34 versus *R*^2^_plasma_ = 0.33, *P*_difference_ = 0.653) and with progression to mild cognitive impairment (hazard ratio (HR)_PET_ = 1.61 (1.48–1.76) versus HR_plasma_ = 1.57 (1.43–1.72), *P*_difference_ = 0.322). Combined plasma and PET models were superior to the single-biomarker models (*R*^2^ = 0.35, *P* < 0.01). Sequential selection using plasma phosphorylated tau at threonine 217 (p-tau217) and then tau-PET reduced the number of participants required for a clinical trial by 94%, compared to a 76% reduction when using plasma p-tau217 alone. Thus, plasma p-tau217 and tau-PET showed similar performance for predicting future cognitive decline in CU individuals, and their sequential use enhances screening efficiency for preclinical AD trials.

## Abstract (web summary)

Ossenkoppele, Salvado et al. show that plasma p-tau217 and tau-PET predict cognitive decline equally well among cognitively unimpaired individuals. Their combined use further boosts prognostic accuracy. A sequential approach can enhance precision and efficiency of preclinical Alzheimer trials.

## Main

In recent years, there has been a substantial increase in the availability of biomarkers that reflect core AD neuropathological hallmarks, specifically amyloid-β (Aβ) plaques and tau neurofibrillary tangles^1^. Tau-PET has shown excellent diagnostic accuracy and strong associations with both concurrent and longitudinal cognitive decline, outperforming established AD biomarkers like amyloid-PET and structural magnetic resonance imaging across the clinical continuum of AD^1–7^. However, tau-PET is expensive and labor intensive and has inadequate availability, and its sensitivity to detect the earliest stages of tau aggregation is limited. This is particularly troublesome in individuals with preclinical AD who harbor AD pathology but have not (yet) developed symptoms^8^. The recent advent of blood-based biomarkers of AD pathology potentially offers a low-cost, non-invasive and scalable alternative^9^. Within the swiftly evolving realm of plasma biomarkers, plasma p-tau217 has demonstrated excellent performance in detecting AD pathology, distinguishing between AD and non-AD neurodegenerative disorders and predicting future clinical progression^10–19^. However, in contrast with tau-PET, plasma p-tau217 provides no regional information on AD pathology, its continuous values are less representative of the full dynamic range of tau pathology and its signal represents a mix of tau and Aβ pathology and is therefore a less tau-specific biomarker^20–22^. In cognitively impaired individuals, tau-PET has shown performance superior to that of plasma p-tau217 in predicting future cognitive decline^23,24^. In CU individuals, however, it is yet unclear whether there is a meaningful difference in prognostic utility between tau-PET and plasma p-tau217 biomarkers^25^. Determining which of the two biomarkers is the strongest predictor of future cognitive deterioration in initially CU individuals is of utmost importance, as clinical trials are increasingly recruiting participants with preclinical AD to enable early intervention^26^. This information would become even more crucial if treatments such as lecanemab^27^ and donanemab^28^ are found to be effective in preclinical AD, as this would require large-scale screening of CU populations for AD pathology.

In previous multicenter cohort studies, we described that tau-PET^29^ and plasma p-tau217 (ref. ^30^) individually showed good prognostic performance in CU populations. To address current knowledge gaps in the literature (that is, which is the best prognostic tau biomarker in CU individuals? Is there added value in the combined use of plasma p-tau217 and tau-PET? Can the two tau biomarkers be effectively implemented in a screening approach for clinical trials?), we performed a large-scale head-to-head comparison study between tau-PET and plasma p-tau217. For tau-PET, we extracted signals from brain regions covering the medial temporal lobe (tau-PET_MTL_) and the temporal neocortex (tau-PET_NEO_)^29^. We assessed their associations with longitudinal cognitive decline and diagnostic progression to mild cognitive impairment (MCI). Additionally, we examined whether and how plasma p-tau217 and tau-PET can be combined to further increase their prognostic accuracy and to optimize recruitment strategies for clinical trials.

## Results

### Participants

**Extended Data Fig. 1:**
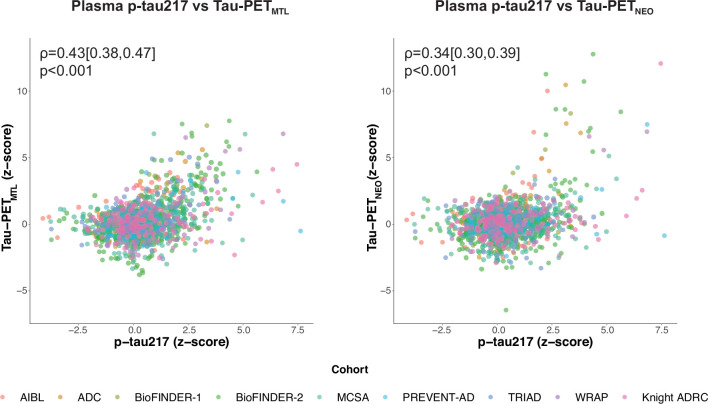
The association between plasma p-tau217 and Tau-PET_MTL_/Tau-PET_NEO_ across cohorts. Spearman correlation analyses between plasma p-tau217 and Tau-PET_MTL_ (left panel) and between plasma p-tau217 and Tau-PET_NEO_ (right panel), color coded by cohort.

We included 1,474 CU participants from nine cohorts with tau-PET and plasma p-tau217 data available at baseline, of whom 408 (27.7%) were Aβ positive on PET (see Table 1 and Supplementary Table 1 for data by cohort). The mean ± s.d. age of the participants was 69.3 ± 10.2 years, 52.8% were females, and the follow-up duration was 3.8 ± 1.8 years. The associations between tau-PET and plasma p-tau217 levels were moderate (plasma p-tau217 versus tau-PET_MTL_, *ρ* (95% confidence interval (CI) = 0.43 (0.38–0.47), *P* < 0.001; plasma p-tau217 versus tau-PET_NEO_, *ρ* = 0.34 (0.30–0.39), *P* < 0.001; Extended Data Fig. 1).

**Table 1:** Participant characteristics

	All participants	Aβ^+^ participants only
*n*	1,474	408
Age, years	69.3 ± 10.2	72.7 ± 8.2
Sex, % female	52.8	55.1
Education, years	14.1 ± 3.3	13.8 ± 3.5
MMSE score	28.8 ± 1.4	28.6 ± 1.4
*APOE* ε4 status, % carriers	37.1	56.6
Aβ status, % positive	27.7	100
Follow-up duration, years	3.8 ± 1.8	3.5 ± 1.8
Follow-up visits, median (range)	3 (1–8)	3 (1–8)
Plasma p-tau217, *z* score (IQR)	0.46 ± 1.41 (1.44)	1.65 ± 1.49 (1.61)
Tau-PET_MTL_, *z* score (IQR)	0.29 ± 1.41 (1.39)	1.29 ± 1.84 (2.39)
Tau-PET_NEO_, *z* score (IQR)	0.18 ± 1.49 (1.28)	0.89 ± 2.18 (1.49)
mPACC5, baseline score	0.04 ± 0.76	−0.25 ± 0.78
mPACC5, annual change	−0.041 ± 0.079	−0.12 ± 0.15
% progression to MCI during max FU	11.0	26.5
% progression to MCI within 2 years	4.6	11.5
% progression to MCI within 4 years	9.5	22.5

Values represent mean ± s.d. unless otherwise indicated. The following variables had missing data: Aβ status (*n* = 1, 0.1%), mPACC (*n* = 98, 6.6%), MMSE (*n* = 8, 0.5%), progression to MCI (*n* = 48, 3.3%).

APOE, apolipoprotein E; FU, follow-up; IQR, interquartile range; max, maximum; MMSE, Mini-Mental State Examination.

### Prediction of future decline in cognitive function

**Fig. 1:**
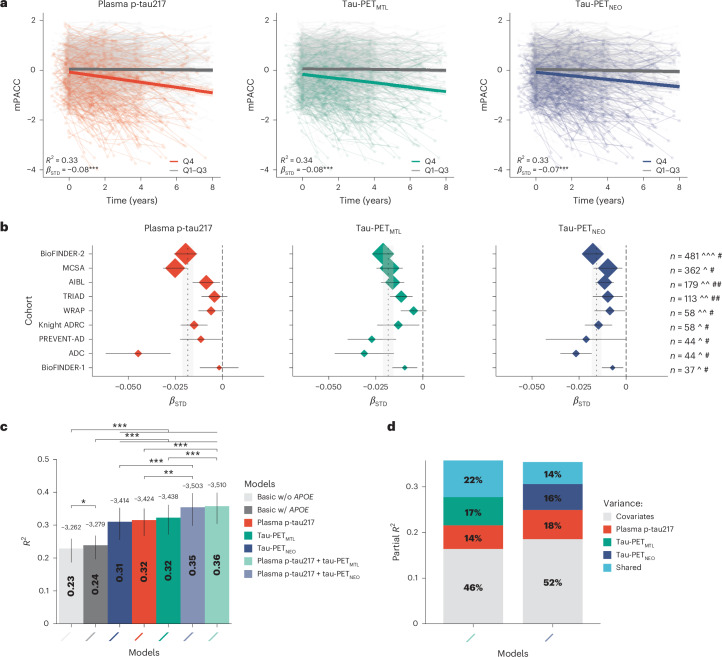
Plasma p-tau217 and tau-PET prediction of future cognitive decline. **a**, Scatterplots showing the association between cognitive change over time on the mPACC5 and the tau biomarkers (Q1–Q3 versus Q4) across all participants. The shaded area indicates the 95% CI around the mean derived from a linear regression model. Note that the standardized *β* (*β*_STD_) coefficients and *R*^2^ statistics relate to the tau biomarker as a continuous variable and that classification into quartiles was performed for visualization purposes only. **b**, Standardized *β* coefficients and 95% CIs from linear regression models for the association between the continuous tau biomarker and annual change in the mPACC5 (adjusted for age, sex, education and *APOE* ε4 status) for each cohort (ordered by sample size). The size of the rhomboid resembles the sample size of each cohort. The vertical dashed line represents standardized *β* = 0, while the thinner vertical dashed line represents the average standardized *β* across all cohorts with the 95% CI indicated in gray. ADC, Amsterdam Dementia Cohort; ADRC, Alzheimer’s Disease Research Center; AIBL, Australian Imaging Biomarkers and Lifestyle Study of Ageing; MCSA, Mayo Clinic Olmsted Study of Aging; PREVENT-AD, Pre-symptomatic Evaluation of Experimental or Novel Treatments for Alzheimer’s Disease; TRIAD, Translational Biomarkers in Aging and Dementia; WRAP, Wisconsin Registry for Alzheimer’s Prevention. **c**, Explained variance (*R*^2^, inside the bar plot) and model fit (corrected Akaike criterion, outside the bar plot) for various models predicting longitudinal change in the mPACC5 across all participants. Error bars represent the 95% CI around the mean derived from a linear regression model. w/, with; w/o, without. **d**, Partial explained variance (*R*^2^) for combined biofluid and neuroimaging models predicting longitudinal change in the mPACC5 across all participants. ‘Shared’ in **d** refers to the explained variance shared between tau-PET and plasma p-tau217 that could not be attributed to a single tau biomarker in the model. Note that we computed cohort-specific *z* scores for plasma p-tau217 and tau-PET using amyloid-PET-negative CU individuals from the same cohort as the reference group. The analyses presented in this figure are based on 1,376 CU individuals. ^[^18^F]flortaucipir PET, ^^[^18^F]MK6240 PET, ^^^[^18^F]RO948 PET. #Lilly plasma p-tau217 immunoassay, ##Janssen plasma p-tau217+ assay. *P < 0.05, **P < 0.01, ***P < 0.001. Exact *P* values can be found in Supplementary Tables 2 and 3.

First, we examined across all participants whether the soluble phosphorylated and aggregated tau biomarkers individually and combined were associated with cognitive decline over time on the modified preclinical Alzheimer cognitive composite (mPACC5). We selected the mPACC5 because it is a sensitive measure that can reliably detect longitudinal changes over time in CU populations and is therefore often used as an outcome measure in research studies and clinical trials focusing on preclinical AD^26,31^. mPACC5 slopes were generated using linear mixed effects models and then used as dependent variables in linear regression models adjusting for age, sex, years of education, *APOE* ε4 carriership and cohort. To account for different assays and tracers across cohorts, we computed cohort-specific *z* scores for plasma p-tau217 and tau-PET using amyloid-PET-negative CU individuals from the same cohort as the reference group. The associations for plasma p-tau217 and tau-PET with annual mPACC5 change are shown both across (Fig. 1a) and within (Fig. 1b) cohorts. The linear regression models showed that plasma p-tau217 concentrations (*R*^2^ = 0.33, corrected Akaike information criterion (AIC_c_) = 7,239.1), tau-PET uptake in the medial temporal lobe (tau-PET_MTL_, *R*^2^ = 0.34, AIC_c_ = 7,232.8) and tau-PET uptake in the temporal neocortex (tau-PET_NEO_, *R*^2^ = 0.33, AIC_c_ = 7,252.6) were all better predictors of longitudinal cognitive decline than basic models that included age, sex, education and cohort with *APOE* ε4 status (*R*^2^ = 0.24, AIC_c_ = 7,507.5) or without *APOE* ε4 status (*R*^2^ = 0.23, AIC_c_ = 7,524.3) (Fig. 1c and Supplementary Tables 2 and 3). There were no significant differences between single-biomarker models, that is, between plasma p-tau217 and tau-PET_MTL_ (*P* = 0.653), between plasma p-tau217 and tau-PET_NEO_ (*P* = 0.752) and between tau-PET_MTL_ and tau-PET_NEO_ (*P* = 0.356). Combined soluble and aggregated tau biomarker models, that is, plasma p-tau217 and tau-PET_MTL_ (*R*^2^ = 0.35, AIC_c_ = 7,146.6) and plasma p-tau217 and tau-PET_NEO_ (*R*^2^ = 0.35, AIC_c_ = 7,149.6), were more strongly associated with longitudinal mPACC5 decline than the single-biomarker models (all *P* < 0.01). The relative contribution of each biomarker in the combined models further indicated their complementary value, as, for example, in the combined plasma p-tau217 and tau-PET_MTL_ model; of all explained mPACC5 variance, 14% was explained by plasma p-tau217 alone, 17% by tau-PET_MTL_ alone, 22% was shared variance and the remaining 46% was explained by covariates (Fig. 1d and Supplementary Table 4). The results were overall consistent across cohorts (Fig. 1b, Supplementary Table 5 and Supplementary Fig. 1) and were largely similar when restricting the sample to CU individuals with at least 4 or 5 years of follow-up data available (Supplementary Fig. 2).

**Extended Data Fig. 2:**
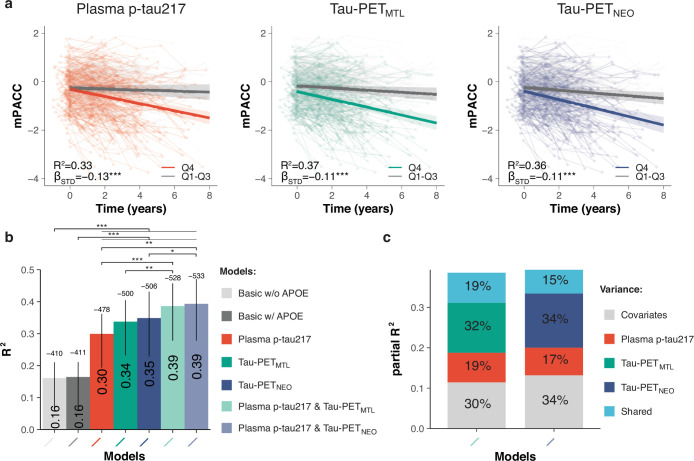
Plasma p-tau217 and Tau-PET prediction of future cognitive decline on the mPACC5 in Aβ+ participants. **a**, Scatterplots showing the association between cognitive change over time on the mPACC5 and the tau biomarkers (Quartile 1-3 vs Quartile 4) across Aβ+ participants. The shadow area indicated the 95% confidence interval around the mean derived from linear regression models. Note that the standardized β-coefficients and R^2^ statistics relate to the tau biomarker as a continuous variable and that classification into quartiles was performed for visualization purposes only. **b**, Explained variance (R^2^, inside the bar plot) and model fit (corrected Akaike criterion, outside the bar plot) for various models predicting longitudinal change on the mPACC5 across all participants. Errorbars represent the 95% CI around the mean derived from linear regression models. **c**, Partial explained variance (R^2^) for combined biofluid and neuroimaging models predicting longitudinal change on the mPACC5 across all participants. “Shared” in panel **c** refers to the explained variance shared between Tau-PET and plasma p-tau217 that could not be attributed to a single tau biomarker in the model. Note that we computed cohort-specific z-scores for plasma p-tau217 and Tau-PET using amyloid-PET negative CU individuals from the same cohort as the reference group. *p<0.05, **p<0.01, ***p<0.001. Exact p-values can be found in Supplementary Tables 6 and 7.

Next, we repeated the same set of analyses with mPACC5 as the outcome measure in amyloid-PET-positive CU individuals only (*n* = 396). This analysis yielded a largely similar pattern compared to analyses of the entire sample (Extended Data Fig. 2 and Supplementary Tables 6 and 7). The single-biomarker models including plasma p-tau217 (*R*^2^ = 0.33, AICc = 2,444.9), tau-PET_MTL_ (*R*^2^ = 0.37, AIC_c_ = 2,420.0) or tau-PET_NEO_ (*R*^2^ = 0.36, AIC_c_ = 2,405.6) outperformed the basic models with and without *APOE* ε4 status (*R*^2^ = 0.20, AICc = 2,535.5 and 2,536.5). Furthermore, there were no differences between the three single-biomarker models (plasma p-tau217 versus tau-PET_MTL_, *P* = 0.257; plasma p-tau217 versus tau-PET_NEO_, *P* = 0.241; tau-PET_MTL_ versus tau-PET_NEO_, *P* = 0.725). Also, combined biomarker models, that is, plasma p-tau217 and tau-PET_MTL_ (*R*^2^ = 0.39, AIC_c_ = 2,389.7) and plasma p-tau217 and tau-PET_NEO_ (*R*^2^ = 0.38, AIC_c_ = 2,377.7), were more strongly associated with longitudinal mPACC5 decline than their respective single-biomarker models (both *P* < 0.02). Among Aβ-positive CU individuals, the relative contribution of the tau-PET measures in the combined model was substantially greater than in the entire study population, that is, tau-PET_MTL_ 32% versus 17% and tau-PET_NEO_ 34% versus 16%, which was not the case for plasma p-tau217 (19% versus 17%; Extended Data Fig. 2c and Supplementary Table 8).

### Prediction of clinical progression to MCI

**Fig. 2:**
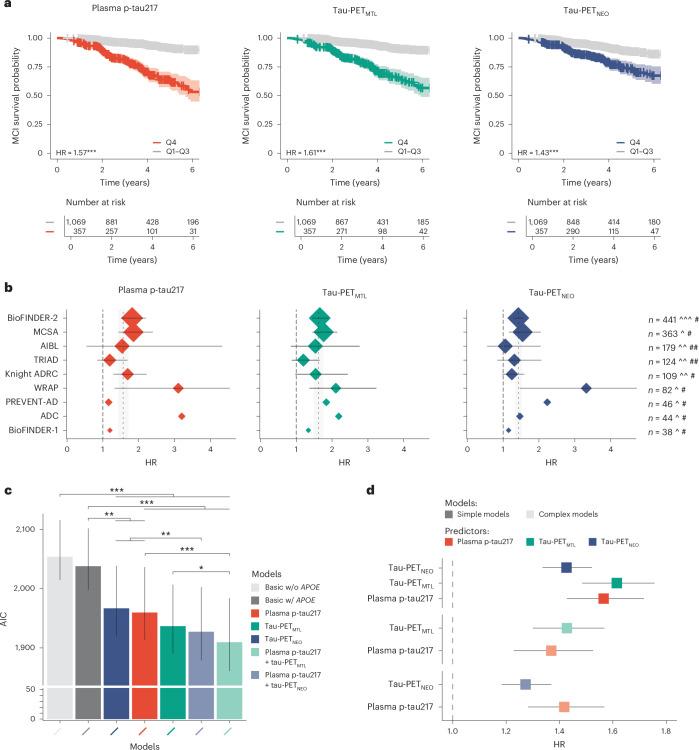
Plasma p-tau217 and tau-PET prediction of progression to MCI. **a**, Survival curves for progression to MCI (Q1–Q3 versus Q4) across all participants, including a table of the total number of participants available at each time point. The shaded area indicates the 95% CI around the mean derived from Cox proportional hazard models. Note that the HR and Akaike information criterion (AIC) statistics relate to the tau biomarker as a continuous variable and that classification into quartiles was only done for visualization purposes. **b**, HRs and 95% CIs from Cox proportional hazard models for the association between the tau continuous biomarker and progression to MCI (adjusted for age, sex, education and *APOE* ε4 status) for each cohort (ordered by sample size). The size of the rhomboid relates to the sample size of each cohort. The vertical dashed line represents standardized HR = 1, while the thinner vertical dashed line represents the average HR across all cohorts with the 95% CI indicated in gray. **c**, Model fit (corrected Akaike criterion) for various models predicting future clinical progression to MCI across all participants. Error bars represent the 95% CI around the mean derived from Cox proportional hazard models. **d**, HRs and 95% CIs around the mean from Cox proportional hazard models in simple models (that is, modeling one tau biomarker at a time, top three HRs) and combined models (that is, modeling plasma and PET tau biomarkers simultaneously, bottom four HRs) when assessing all cohorts together. Vertical dashed line shows the HR = 1 (no effect). Note that we computed cohort-specific *z* scores for plasma p-tau217 and tau-PET using amyloid-PET-negative CU individuals from the same cohort as the reference group. The analyses presented in this figure are based on 1,426 CU individuals. ^[^18^F]flortaucipir PET, ^^[^18^F]MK6240 PET, ^^^[^18^F]RO948 PET. #Lilly plasma p-tau217 immunoassay, ##Janssen plasma p-tau217+ assay. *P < 0.05, **P < 0.01, ***P < 0.001. Exact *P* values can be found in Supplementary Tables 9 and 10.

Next, we examined across all participants whether the tau biomarkers individually and combined were associated with the rate of clinical progression to MCI using Cox proportional hazard models, adjusting for age, sex, years of education, cohort and *APOE* ε4 carriership. The associations for plasma p-tau217 and tau-PET with clinical progression to MCI are shown both across (Fig. 2a) and within (Fig. 2b) cohorts. The analysis revealed that higher baseline plasma p-tau217 (HR = 1.57 (1.43–1.72), AIC_c_ = 1,960), tau-PET_MTL_ (HR = 1.61 (1.48–1.76), AIC_c_ = 1,937) and tau-PET_NEO_ (HR = 1.43 (1.30–1.52), AIC_c_ = 1,967) levels were all associated with an increased risk for future progression to MCI (all *P* < 0.001; Fig. 2a,b and Supplementary Tables 9 and 10). The individual fluid versus neuroimaging tau biomarker models did not differ from each other (plasma p-tau217 versus tau-PET_MTL_, *P* = 0.322; plasma p-tau217 versus tau-PET_NEO_, *P* = 0.750), while the performance of tau-PET_MTL_ showed a trend toward better performance than tau-PET_NEO_ (*P* = 0.059). The fit was improved for the plasma p-tau217 model when adding tau-PET_MTL_ (∆AIC_c_ = −50, *P* ≤ 0.001) or tau-PET_NEO_ (∆AIC_c_ = −33, *P* = 0.009; Supplementary Table 10). Likewise, the model fit also improved when adding plasma p-tau217 to tau-PET_MTL_ (∆AIC_c_ = −27, *P* = 0.017) and to tau-PET_NEO_ (∆AIC_c_ = −40, *P* = 0.004). The results were largely consistent across cohorts (Fig. 2b, Supplementary Table 11 and Supplementary Fig. 3) and were largely similar when restricting the sample to CU individuals with at least 4 or 5 years of follow-up data available (Supplementary Fig. 4).

**Extended Data Fig. 3:**
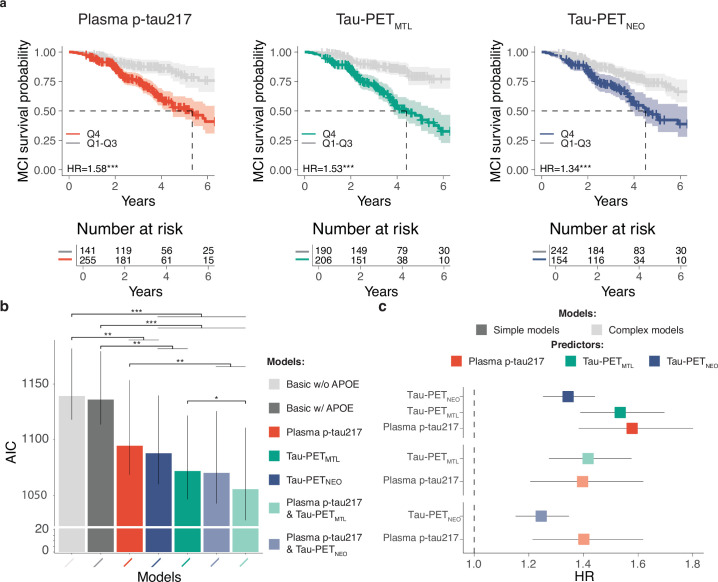
Plasma p-tau217 and Tau-PET prediction of future progression to MCI in Aβ+ participants. Note that all analyses are performed in Aβ+ participants only. **a**, Survival curves for progression to mild cognitive impairment (Quartile 1-3 vs Quartile 4) across in Aβ+ participants, including a table of total number of participants available at each time point. The shadow area indicated the 95% confidence interval around the mean derived from Cox proportional hazard models. Note that the HR and AIC statistics relate to the tau biomarker as a continuous variable, and that classification into quartiles was only done for visualization purposes. **b**, Model fit (corrected Akaike criterion) for various models predicting future clinical progression to mild cognitive impairment across all participants. Error bars represent the 95% CI around the mean derived from Cox proportional hazard models. **c**, Hazard ratios and 95% confidence intervals around the mean derived from Cox proportional hazard models in simple models (that is, modeling one tau biomarker at the time, top three HR’s) and combined models (that is, modeling plasma and PET biomarkers simultaneously, bottom four HR’s). Note that we computed cohort-specific z-scores for plasma p-tau217 and Tau-PET using amyloid-PET negative CU individuals from the same cohort as the reference group. * p<0.05, **p<0.01, ***p<0.001. Exact p-values can be found in Supplementary Tables 12 and 13.

When repeating the same set of analyses in Aβ-positive CU individuals only (*n* = 396), we found a largely similar pattern (Extended Data Fig. 3 and Supplementary Tables 10 and 11). Baseline plasma p-tau217 (HR = 1.58 (1.38–1.80), AIC_c_ = 1,094), tau-PET_MTL_ (HR = 1.53 (1.39–1.70), AIC_c_ = 1,072) and tau-PET_NEO_ (HR = 1.34 (1.25–1.44), AIC_c_ = 1,088) levels were all associated with an increased risk for future progression to MCI (all *P* < 0.001). The individual tau biomarker models did not differ from each other (plasma p-tau217 versus tau-PET_MTL_, *P* = 0.200; plasma p-tau217 versus tau-PET_NEO_, *P* = 0.711; tau-PET_MTL_ versus tau-PET_NEO_, *P* = 0.209). Adding tau-PET to plasma p-tau217 models improved the model fit when adding tau-PET_MTL_ (∆AIC_c_ = −39, *P* = 0.007) and tau-PET_NEO_ (∆AIC_c_ = −24, *P* = 0.031; Supplementary Tables 12 and 13). The model fit also slightly improved when adding plasma p-tau217 to either tau-PET_MTL_ (∆AIC_c_ = −17, *P* = 0.042) and at trend level for tau-PET_NEO_ (∆AIC_c_ = −18, *P* = 0.061).

### A two-step approach to reduce the sample size in clinical trials

**Fig. 3:**
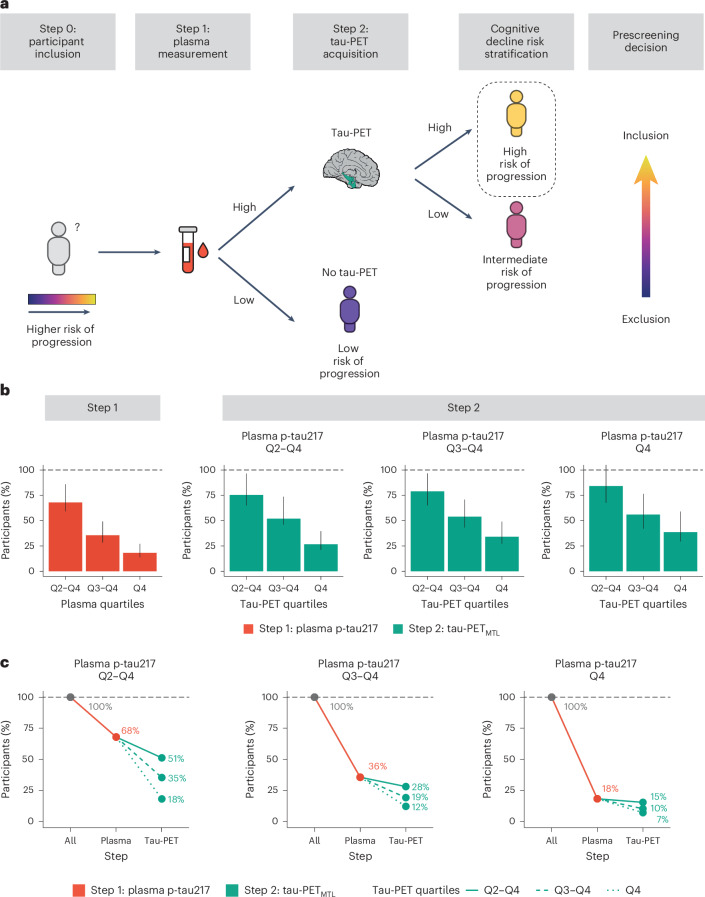
A conceptual two-step recruitment approach for clinical trials in preclinical AD using the mPACC as the outcome measure. **a**, Conceptual framework of a sequential two-step recruitment strategy of a clinical trial in preclinical AD using a cognitive endpoint. **b**, The obtained sample size reduction using sample selection based on different percentiles (75th, 50th and 25th) of baseline plasma p-tau217 levels in step 1 followed by selection based on the same percentiles (75th, 50th and 25th) of the tau-PET_MTL_ measurement in step 2 with the mPACC5 as the primary endpoint. Note that 100% in step 2 refers to the participants selected by plasma p-tau217 in step 1. Error bars represent the 95% CI around the mean derived from linear effects models. **c**, The calculated sample size reductions for various plasma p-tau217 and tau-PET_MTL_ quartile combinations. Red lines represent step 1 with plasma p-tau217, and green lines represent step 2 with tau-PET_MTL_. Different line styles represent different quartiles of tau-PET_MTL_ from those participants already selected from step 1. Dashed black lines represent 100% of participants needed without that step. Calculations in **b**,**c** are based on the assumption of 80% power to detect a 30% change in the mPACC5 in a 4-year trial. The analyses presented in this figure are based on 1,376 CU individuals.

**Fig. 4:**
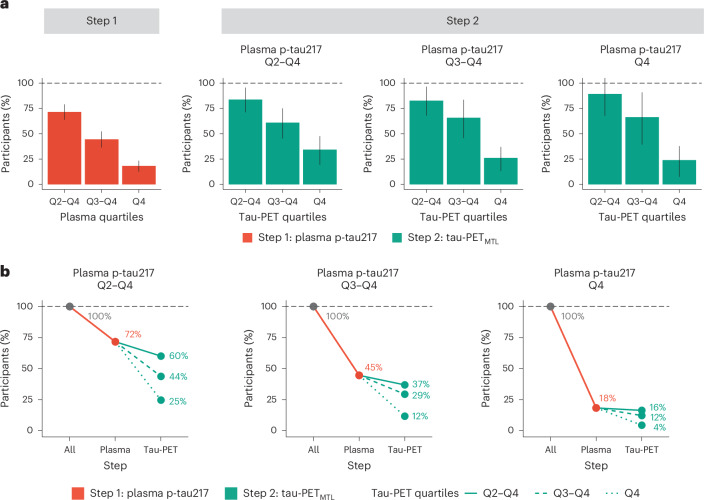
Clinical trial sample size reductions through a two-step recruitment strategy when using clinical progression to MCI as an outcome measure. **a**, The obtained sample size reduction using sample selection based on different percentiles (75th, 50th and 25th) of baseline plasma p-tau217 levels in step 1 followed by selection based on the same percentiles (75th, 50th and 25th) of the tau-PET_MTL_ measurement in step 2 with clinical progression to MCI as the primary endpoint. Note that 100% in step 2 refers to the participants selected by plasma p-tau217 in step 1. Error bars represent the 95% CI around the mean. **b**, The calculated sample size reductions for various plasma p-tau217 and tau-PET_MTL_ quartile combinations. Red lines represent step 1 with plasma p-tau217, and green lines represent step 2 with tau-PET_MTL_. Different line styles represent different quartiles of tau-PET_MTL_ from those participants already selected from step 1. Dashed black lines represent 100% of participants needed without that step. The analyses presented in this figure are based on 1,426 CU individuals.

**Extended Data Fig. 4:**
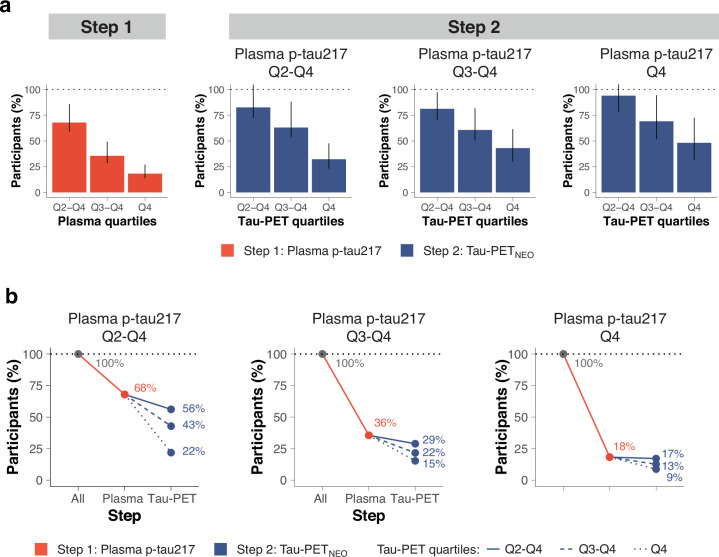
A two-step recruitment approach for clinical trials in preclinical Alzheimer’s disease using the mPACC as outcome measure with plasma p-tau217 and Tau-PET_NEO_. Note that all analyses are performed using Tau-PET_NEO_ only. **a**, the obtained sample size reduction using sample selection based on different percentiles (75th, 50th and 25th) of baseline plasma p-tau217 levels in step 1 followed by the selection based on the same percentiles (75th, 50th and 25th) of the Tau-PET_NEO_ measurement in step 2 with mPACC5 as the primary endpoint. Note that 100% in step 2 refers to the participants selected by plasma p-tau217 in step 1. Error bars represent the 95% CI around the mean derived from mixed effects models. **b**, shows the calculated sample size reductions for various plasma p-tau217 and Tau-PET_NEO_ quartile combinations. Calculations are based on the assumption of 80% power to detect a 30% change on the mPACC5 in a 4-year trial. Red lines represent step 1 with plasma p-tau217 and blue lines represent step 2 with Tau-PET_NEO_. Different line styles represent different quartiles of Tau-PET_NEO_ from those subjects already selected from step 1. Dotted black lines represent 100% participants needed without that step.

**Extended Data Fig. 5:**
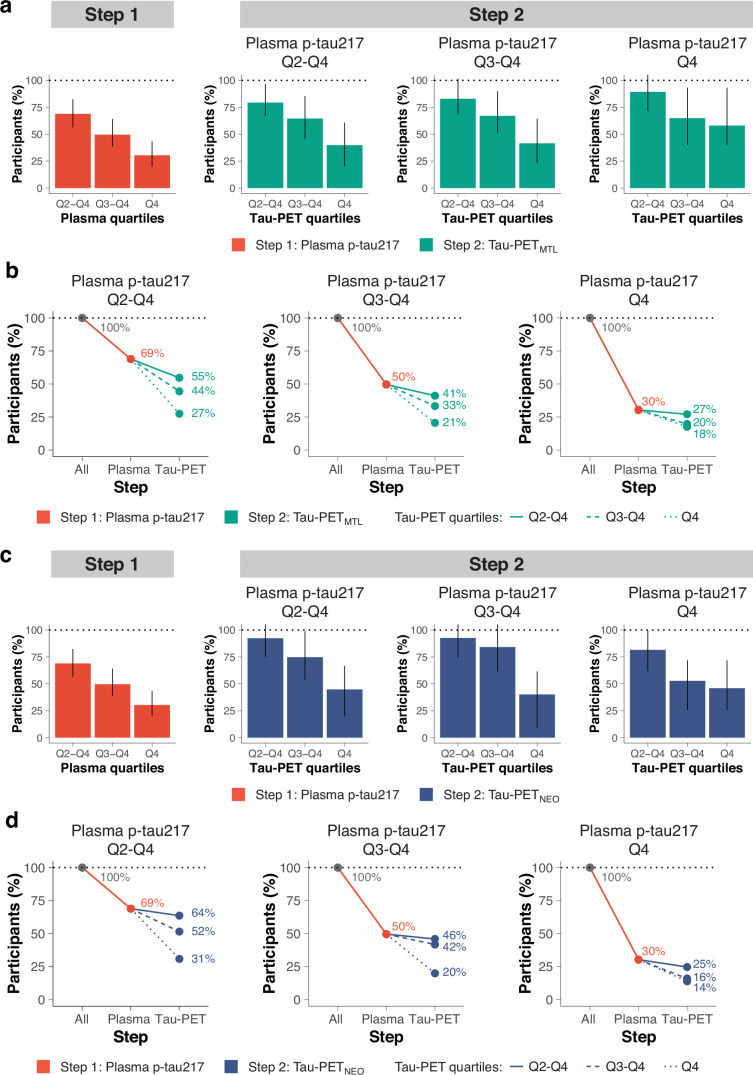
A two-step recruitment approach for clinical trials in preclinical Alzheimer’s disease using the mPACC as outcome measure in Aβ+ participants. Note that all analyses are performed in Aβ+ participants only. **a**, the obtained sample size reduction using sample selection based on different percentiles (75th, 50th and 25th) of baseline plasma p-tau217 levels in step 1 followed by the selection based on the same percentiles (75th, 50th and 25th) of the Tau-PET_MTL_ measurement in step 2 with mPACC5 as the primary endpoint. Note that 100% in step 2 refers to the participants selected by plasma p-tau217 in step 1. Error bars represent the 95% CI around the mean. **b**, shows the calculated sample size reductions for various plasma p-tau217 and Tau-PET_MTL_ quartile combinations. Calculations are based on the assumption of 80% power to detect a 30% change on the mPACC5 in a 4-year trial. Red lines represent step 1 with plasma p-tau217 and green lines represent step 2 with Tau-PET_MTL_. Different line styles represent different quartiles of Tau-PET_MTL_ from those subjects already selected from step 1. Dotted black lines represent 100% participants needed without that step. **c,d** same as above, but now for Tau-PET_NEO_ instead of Tau-PET_MTL._

**Extended Data Fig. 6:**
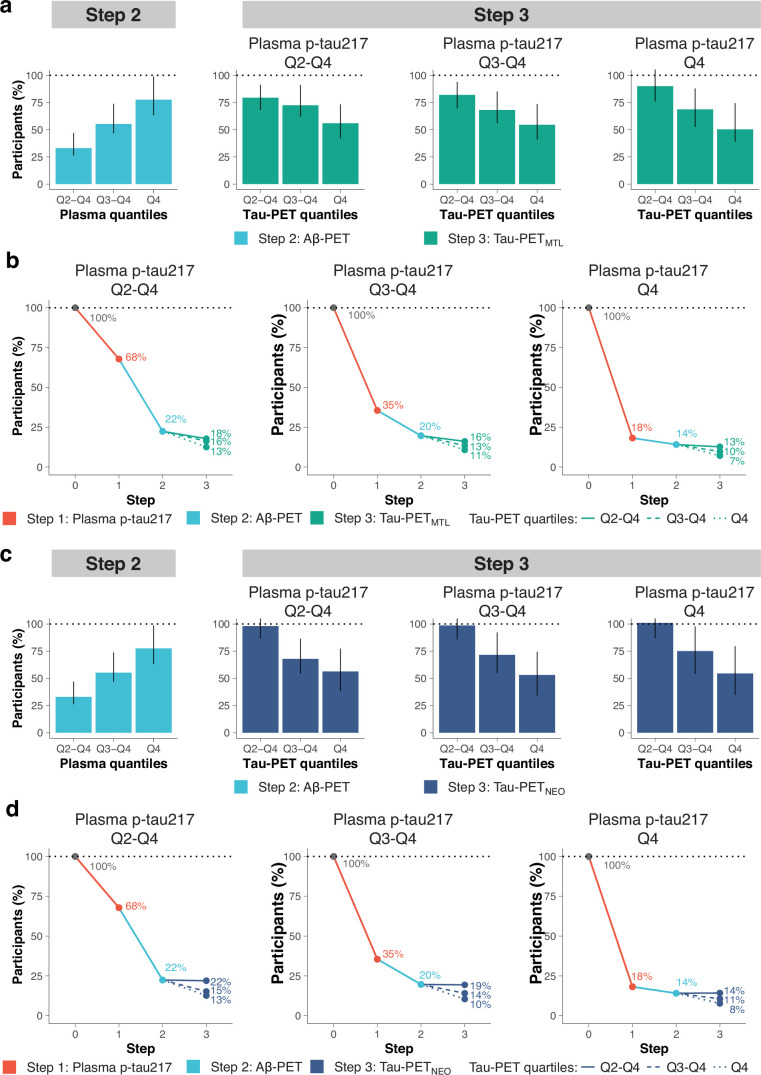
A three-step recruitment approach for clinical trials in preclinical Alzheimer’s disease, now also implementing Amyloid-PET. **a**, the obtained sample size reduction using sample selection based on different percentiles (75th, 50th and 25th) of baseline plasma p-tau217 levels in step 1, followed by adding Amyloid-PET positivity (step 2) followed by the selection based on the same percentiles (75th, 50th and 25th) of the Tau-PET_MTL_ measurement in step 3 with mPACC5 as the primary endpoint. Error bars represent the 95% CI around the mean. Red lines represent step 1 with plasma p-tau217, light blue lines represent step 2 with Amyloid PET and green lines step 3 with Tau-PET_NEO_. Different line styles represent different quartiles of Tau-PET_NEO_ from those subjects already selected from steps 1 and 2. **b**, shows the calculated sample size reductions for various plasma p-tau217 (quartiles), Amyloid-PET (status) and Tau-PET_MTL_ (quartiles) combinations. Calculations are based on the assumption of 80% power to detect a 30% change on the mPACC5 in a 4-year trial. **c,d** same as above, but now for Tau-PET_NEO_ instead of Tau-PET_MTL_.

Next, we tested whether and how a two-step sequential approach (that is, plasma p-tau217 followed by tau-PET) could reduce the number of participants needed for a preclinical AD trial using longitudinal changes in cognitive function as the primary outcome. Substantial sample size reductions can already be achieved in the first step, that is, using only plasma p-tau217. When using the mPACC5 as the outcome measure, assuming 80% power and *α* = 0.05 in a 4-year clinical trial with annual repeated testing, selecting participants with plasma p-tau217 levels in quartiles 2–4 (that is, Q2–Q4, excluding the lowest 25% of plasma p-tau217) would result in a 32% (14–41%) reduction in the number of required participants compared to including the entire study population (Fig. 3 and Supplementary Table 14). Selecting participants in plasma p-tau217 Q3–Q4 and Q4 further reduced the required sample size by 64% (51–72%) and 82% (73–86%), respectively. Using clinical progression to MCI as an outcome measure yielded similar results, that is, plasma p-tau217 Q2–Q4, 28% (21–36%); Q3 and Q4, 55% (48–64%); and Q4, 82% (77–87%) (Fig. 4 and Supplementary Table 14). In the second step, tau-PET measures further reduced the required sample size. For example, in the population with plasma p-tau217 concentrations in Q3 and Q4, selecting participants with tau-PET in Q4 would further reduce the sample size from 64% (51–72%) (plasma p-tau217) to 88% (81–90%) (tau-PET_MTL_; Fig. 3) or to 85% (76–89%) (tau-PET_NEO_; Extended Data Fig. 4) when using the mPACC5 as the outcome measure. As another example in the population with plasma p-tau217 concentrations in Q4, selecting participants with tau-PET in Q3 and Q4 would further reduce the sample size 82% (77–87%) (plasma p-tau217) to 88% (82–94%) (tau-PET_MTL_; Fig. 4) or to 94% (92–97%) (tau-PET_NEO_; Supplementary Fig. 5) when using clinical progression to MCI as the outcome measure. The estimated sample size reductions for all plasma p-tau217 and tau-PET quartile combinations are presented in Supplementary Table 14. Repeating the same set of analyses but now restricted to Aβ-positive CU individuals showed that similarly large sample size reductions can be achieved in this population (Extended Data Fig. 5). Finally, we considered a scenario in which we tested the sequence plasma p-tau217, followed by amyloid-PET and then tau-PET when using change in the mPACC as an outcome measure (Extended Data Fig. 6). The results suggest that, after initial plasma p-tau217 screening (step 1), incorporating amyloid-PET positivity (step 2) further reduces the number of trial participants needed. This number can in turn subtly be lowered further by implementing tau-PET (step 3). Changing the outcome measure from the mPACC5 to progression to MCI yielded very similar results (Supplementary Fig. 6).

### Characterization of combined plasma p-tau217 and tau-PET groups

**Fig. 5:**
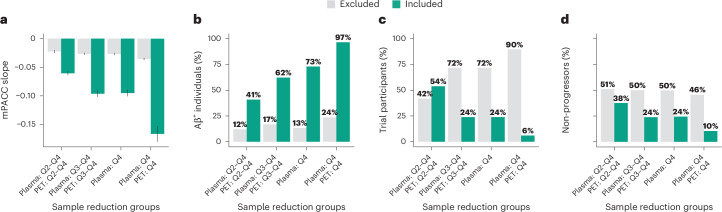
Characterization of different plasma p-tau217 and tau-PET_MTL_ groups in relevant trial measures. This figure shows how different group compositions based on their baseline plasma p-tau217 and tau-PET_MTL_ levels are related to various relevant trial metrics, including the annual mPACC5 slope (**a**, *n* = 1,376), the proportion of Aβ^+^ individuals (**b**, *n* = 1,473), the proportion of individuals from the entire population who would be included in a clinical trial based on the group definitions described on the *x* axis (**c**, all participants) and the proportion of ‘non-progressors’ on the mPACC5 (defined as slope > −0.016, see the Methods for details) (**d**, *n* = 1,376). Error bars in **a** represent the 95% CI. More efficient trials are expected with lower mPACC slopes and higher percentages of Aβ^+^ individuals and trial participants but lower percentages of non-progressors.

Finally, we aimed to characterize the groups resulting from combining different plasma p-tau217 and tau-PET_MTL_ quartiles in terms of rates of mPACC5 decline, Aβ positivity and the proportion of participants who would be included in the trial as well as the proportion of ‘non-progressors’ based on each combination relative to the full dataset. For this purpose, we created four different groups based on increasingly restrictive tau biomarker combinations: (1) a ‘liberal’ group consisting of participants with plasma p-tau217 levels in Q2–Q4 and then tau-PET_MTL_ uptake in Q2–Q4 of those selected by plasma, (2) a ‘moderate’ group consisting of plasma p-tau217 levels in Q3 and Q4 and then tau-PET_MTL_ uptake in Q3 and Q4, (3) a ‘plasma p-tau217 Q4-only’ group consisting of individuals with plasma p-tau217 levels in Q4 regardless of tau-PET and (4) a ‘conservative’ group consisting of plasma p-tau217 levels in Q4 and then tau-PET_MTL_ uptake in Q4. Fig. 5 indicates a progressively worse outcome for individuals from approach (1) to (4), with more rapid decline on the mPACC5 from the liberal (1) to the conservative (4) threshold approach (from standardized *β* = −0.06 ± 0.09 to −0.17 ± 0.13; Fig. 5a), an increasing proportion of Aβ-positive individuals (from 41% to 97%; Fig. 5b) and a smaller proportion of ‘non-progressors’ on the mPACC5 (from 38% to 10%; Fig. 5d). Furthermore, the proportion of participants who would be included in a hypothesized clinical trial with the mPACC5 as an outcome measure decreased from 54% (of the entire population) when using the liberal threshold approach to only 6% when using the conservative threshold approach (Fig. 5c). Notably, there were no group differences between the plasma p-tau217 Q4-only approach versus the moderate combined threshold approach for mPACC5 decline (standardized *β* = −0.09 ± 0.10 versus −0.10 ± 0.11), the proportion of participants selected (24.0% versus 24.0%) and the proportion of ‘non-progressors’ (24.4% versus 23.8%) and only a modest difference in Aβ positivity (that is, 73.0% versus 62.2%). The same set of analyses using tau-PET_NEO_ instead of tau-PET_MTL_ yielded similar results (Supplementary Fig. 7). Detailed group characterizations for all the other possible combinations are presented in Supplementary Table 15 (that is, Aβ positivity, mPACC5 decline and non-progressors) and Supplementary Table 16 (that is, age, sex, *APOE* status and years of education). Additionally, in the BioFINDER-2 study, we applied predefined cutoffs for both plasma p-tau217 and tau-PET and found that tau biomarker-positive individuals showed characteristics very similar to those of individuals in Q4 concerning mPACC5 slopes (plasma p-tau217 positive, −0.22 ± 0.15; tau-PET_MTL_ positive, −0.19 ± 0.15), percent of Aβ-positive individuals (plasma p-tau217, 90%; tau-PET_MTL_, 100%), percent of participants included in the trial (plasma p-tau217, 4%; tau-PET_MTL_, 8%) and percent of non-progressors (plasma p-tau217, 10%; tau-PET_MTL_, 13%; Supplementary Fig. 8).

We also calculated the projected costs that could be saved in a hypothetical trial with mPACC5 or MCI progression rates as an outcome measure using the same four groups as above. When assuming a 1:15 ratio (that is, cost of one tau-PET scan equals 15 plasma p-tau217 assessments) and using tau-PET_MTL_, both the plasma p-tau217 Q4-only (3) group (96.9%) and the conservative combined (4) group (96.6% cost reduction) yielded substantially higher cost reductions than the moderate (2) group (85.6%) and especially the liberal (1) group (54.4%) when using mPACC as an endpoint (Supplementary Fig. 9a, tau-PET_NEO_ results are presented in Supplementary Fig. 9b). Using the same ratio (1:15) but now using clinical progression as the endpoint, the plasma p-tau217 Q4-only (3) group yielded the highest cost reduction (88.6%), followed by the conservative combined (4) group (70.9%), the moderate (2) group (53.4%) and the liberal (1) group (25.1%; Supplementary Fig. 10a, tau-PET_NEO_ results are presented in Supplementary Fig. 10b).

## Discussion

In this multicohort study, we investigated whether tau-PET, a marker of aggregated tau pathology, or plasma p-tau217, a marker of soluble hyperphosphorylated tau, is more strongly associated with future cognitive decline among 1,474 CU individuals and whether they would provide complementary information in screening approaches for clinical trials. This is a timely research question, as both tau biomarkers are frequently incorporated into clinical trials and often in combination, as they reflect different aspects of tau pathophysiology. According to the revised AD criteria by the Alzheimer’s Association Workgroup, plasma p-tau217 is a core 1 biomarker (T_1_) of phosphorylated and secreted AD tau, while tau-PET is a core 2 biomarker (T_2_) of AD tau proteinopathy^32^. In this study, we observed comparably strong associations between plasma p-tau217 and tau-PET with cognitive decline on a sensitive global cognitive composite test (that is, mPACC5) and with clinical progression to MCI in CU individuals. Importantly, models including both plasma p-tau217 and tau-PET consistently outperformed single-biomarker models, suggesting that plasma p-tau217 and tau-PET provide complementary information. Simulations showed that a two-step approach (that is, plasma p-tau217 first, followed by tau-PET in individuals with high plasma p-tau217 only) could substantially reduce the number and the cost of recruiting participants for a preclinical AD clinical trial with change in mPACC5 or progression to MCI as the primary endpoint. We conclude that plasma p-tau217 and tau-PET showed similar associations with future cognitive decline in a CU population, that both tau biomarkers provide complementary information and that their sequential use (that is, plasma p-tau217 followed by tau-PET in a subset with high plasma p-tau217) is useful for screening in clinical trials in preclinical AD.

A main finding of this study is that there were no statistical differences between plasma p-tau217 concentrations and tau-PET uptake in their associations with future cognitive changes in a CU population. This is in contrast with cognitively impaired populations, in which tau-PET generally outperforms plasma p-tau217 in terms of prognostic accuracy^23,24^. This discrepancy between disease stages might be explained by the differences in underlying pathophysiology and subsequent temporal dynamics of the two tau biomarkers. Plasma p-tau217 measures the hyperphosphorylated tau protein in soluble forms and has been shown to change very early in the disease process^33^. This was also observed in the lower range of our population, where plasma p-tau217 *z* scores started to increase while levels of tau-PET retention remained unchanged (Extended Data Fig. 1). Post-mortem studies indicated that ante-mortem plasma p-tau217 levels are associated with the density of both neurofibrillary tau tangle and Aβ plaque pathology in the brain^21,34^. It is conceivable that this mix of Aβ- and tau-related signals reflected by plasma p-tau217 levels contributed to its non-inferiority versus tau-PET for predicting cognitive change over time in this early population. For instance, Aβ toxicity might impact synaptic function or neuroinflammation, which could subsequently influence cognitive performance^35^. By contrast, tau-PET signal largely represents the presence of aggregated paired helical filaments of the tau protein forming insoluble neurofibrillary tangles^36^. In vivo studies have shown that positive tau-PET scans are relatively rare in CU individuals (that is, ~5–10% among Aβ-positive CU individuals in a temporal meta-region of interest)^3,4^, and post-mortem studies have indicated that a positive [^18^F]flortaucipir tau-PET scan (quantitatively or by visual read) represents tau pathology in Braak stages IV and above^37,38^. This may also explain why the relative contribution to cognitive decline of tau-PET versus plasma p-tau217 was greater in Aβ-positive individuals than in Aβ-negative CU individuals. Altogether, this indicates that substantial changes on a tau-PET scan occur in rather advanced clinical and biological stages of AD, which may explain why tau-PET in the current mix of Aβ-positive and Aβ-negative CU individuals did not outperform plasma p-tau217 in predicting future cognitive decline.

Another important finding of the current study was that we did not observe marked differences between tau-PET_MTL_ and tau-PET_NEO_ in this CU population, while previous studies consistently showed that tau-PET signal in neocortical areas is a better predictor of future clinical progression than tau-PET signal in medial temporal lobe regions, especially in cognitively impaired individuals^5,39–43^. This result may also appear contradictory with our previous study in which the tau-PET_NEO_-positive group exhibited a considerably worse prognosis than the tau-PET_MTL_-positive group^29^. This discrepancy can be explained by the different group definitions used in the current study (quartile based) compared to those of the previous study (binary cutoffs). In the previous study, the tau-PET_NEO_-positive group comprised a relatively small proportion of the overall CU population (only ~4%). As a result, even in the highest quartile (Q4) of the current study, most individuals are tau-PET negative (that is, 56% for tau-PET_MTL_ and 84% for tau-PET_NEO_). The scarcity of tau-PET_NEO_-positive individuals in the current study (and in CU populations in general) attenuates the overall association between tau-PET and cognitive decline and dilutes the association even further for the tau-PET_NEO_ group. We conclude that the ‘negative’ result for the tau-PET_NEO_ group is a valid finding but only within the specific context of this study (that is, evaluating a CU population using a quartile-based approach).

Because there were no clear distinctions between the two tau biomarkers in individual models, plasma p-tau217 should be prioritized over tau-PET as a standalone screening tool in CU populations due to its major practical advantages. An exception could be when the goal is to demonstrate target engagement of an anti-tau agent, in which case tau-PET might be the preferred tau biomarker^20^. Compared to single-biomarker models, simultaneous modeling of plasma p-tau217 and tau-PET consistently led to a more accurate forecast of subsequent cognitive decline. We therefore proposed a conceptual framework of a two-step sequential approach for clinical trial design (Fig. 3a), starting with participant selection based on plasma p-tau217 followed by tau-PET, and tested a simplified version of this strategy. This approach decreased the cost of selecting appropriate participants for clinical trials by drastically reducing the number of required tau-PET scans and participants for screening. This was mainly achieved by selecting, from the entire CU study population, the subset of individuals that are at highest risk for short-term cognitive decline due to their elevated levels of both baseline plasma p-tau217 and tau-PET. In such a workflow, the participants’ eligibility in terms of inclusion (for example, age, cognitive status) and exclusion (for example, the absence of major neurological or psychiatric disorders) criteria would be assessed first. Next, participants would be further triaged based on their plasma p-tau217 levels, where ‘low or negative’ participants would not undergo a tau-PET scan as their risk for future cognitive decline would be low, while ‘high or positive’ participants would undergo a tau-PET scan to further refine their risk profile^44^. The degree of tau-PET uptake can then be used to assign CU individuals on a continuum ranging from intermediate (that is, ‘high or positive’ plasma p-tau217, ‘low or negative’ tau-PET) to high (that is, ‘high or positive’ results on both plasma p-tau217 and tau-PET) risk for future progression. The final prescreening decision will be based on factors such as the acceptable rate of screening failures, trial duration, expected effect size of the drug or intervention and whether the trial is a primary or secondary intervention strategy. The framework depicted in Fig. 3a is primarily conceptual, and we recognize that numerous crucial decisions need to be made during its implementation. This includes, among other factors, determining the criteria for ‘high or positive’ versus ‘low or negative’ for both plasma p-tau217 and tau-PET, deciding whether to use p-tau217 alone or in combination with other plasma biomarkers (for example, Aβ_42__/__40_), identifying the target region of interest for tau-PET quantification, selecting a quantitative threshold and/or visual read metric for tau-PET and potentially adding amyloid-PET to the proposed workflow (Extended Data Fig. 6)^45,46^.

The main strength of this study is the multicenter approach that yielded a sufficient sample size for a robust head-to-head comparison between tau-PET and plasma p-tau217 as well as a thorough assessment of their potential complementary value. The main limitations of the study are related to the inherent challenges of a multicenter study that was not co-designed from the start. First and foremost, we did not have plasma p-tau217 cutoffs available from all cohorts and instead used either continuous (*z* scores) or categorical (quartiles) data for the analyses. Also, we aimed to optimize pooling of data across cohorts by standardizing biomarker values using CU Aβ-negative participants. We acknowledge, however, that there may still be residual heterogeneity caused by use of different amyloid-PET and tau-PET tracers and different p-tau217 assays as well as the use of different neuropsychological tests to generate mPACC5 scores. To mitigate this, we present both the pooled (main report) and cohort-specific (supplementary) results. Additionally, tau-PET may have been at a slight disadvantage compared to plasma p-tau217, as tau-PET data were more often analyzed locally, whereas plasma p-tau217 was predominantly analyzed centrally at Lund University. In one of the cohorts (that is, BioFINDER-2) in which we had predefined cutoffs available for both plasma p-tau217 and tau-PET, we found that tau biomarker-positive individuals showed characteristics very similar to those of individuals in Q4 concerning, for example, mPACC5 slopes and percent of Aβ-positive individuals (Supplementary Fig. 8). However, future large-scale studies using predefined cutoffs for both plasma p-tau217 and tau-PET, preferentially in a more diverse population in terms of ethnicity, socioeconomic status and medical comorbidities, are of importance to establish the generalizability of our findings. Another potential threat to the generalizability of our findings is the intentional focus on CU individuals. This may have introduced several biases, including survival bias (that is, the CU individuals in this study may systematically differ from the general population in terms of genetics or lifestyle, especially among those with elevated tau biomarkers) and selection bias (that is, CU individuals were recruited through varying methods across cohorts). Related to the latter, inspection of demographic data (Table 1) suggests that this multicenter study population is enriched for AD risk factors. For example, the proportion of *APOE* ε4 carriers in our study was higher (37%) than in the general population (15–30%, depending on ethnic group variations)^47,48^. This pattern was also observed within the Aβ-positive group, in which 57% were *APOE* ε4 carriers, compared to 51% in an independent multicenter study^49^.

In summary, our data suggest that plasma p-tau217 and tau-PET show similar associations with future cognitive decline and clinical progression in a CU population. We also showed that plasma p-tau217 and tau-PET provide complementary information and that a two-step approach (that is, plasma p-tau217 followed by tau-PET) substantially reduces the number of required tau-PET scans and screened participants. Altogether, our data support the feasibility of a clinical trial design in which all participants undergo screening with plasma p-tau217, but only a subset with high or abnormal plasma p-tau217 will undergo tau-PET.

## Methods

### Participants

We included 1,474 participants from the Swedish BioFINDER-1 (*n* = 39, NCT01208675) and BioFINDER-2 (*n* = 481, NCT03174938) studies at Lund University^7,43^, the MCSA^50^ (*n* = 363), the AIBL^51^ (*n* = 180), the Knight ADRC at Washington University (*n* = 109), TRIAD (*n* = 124) and PREVENT-AD (*n* = 52) at McGill University, the WRAP (*n* = 82) at the University of Wisconsin–Madison and the SCIENCe project^52^, which is part of the ADC (*n* = 44). The overlap of CU participants between the current and our previous multicenter studies where we assessed the prognostic performance of tau-PET and plasma p-tau217 individually was 630 of 1,474 (43%) and 170 of 1,474 (12%), respectively. A brief description of each cohort is provided in Supplementary Table 17. All participants were (1) CU at baseline, defined by neuropsychological test scores within the normative range given an individual’s age, sex and educational background, (2) had amyloid-PET available to determine Aβ status, (3) underwent a tau-PET scan and blood sampling within a maximum interval of 1 year and (4) had at least one clinical follow-up visit available. Follow-up data were collected until 1 November 2023. Written informed consent was obtained from all participants and local institutional review boards for human research approved in the study. This includes the Mayo Clinic and Olmsted Medical Center institutional review boards for the MSCA, the regional ethics committee at Lund University for BioFINDER-1 and BioFINDER-2, institutional human research ethics committees of Austin Health, St. Vincent’s Health, Hollywood Private Hospital and Edith Cowan University for the AIBL, the medical ethics review committee of the Amsterdam University Medical Center for the ADC, the McGill Research Ethics Board for PREVENT-AD and TRIAD, the Washington University School of Medicine in St. Louis for the Knight ADRC and the University of Wisconsin Institutional Review Board for the WRAP.

### Amyloid-PET status

Aβ status was determined using center-specific cutoffs or visual read metrics using [^18^F]flutemetamol PET for BioFINDER-1 and BioFINDER-2, [^11^C]Pittsburgh compound B PET was used for the MCSA, the Knight ADRC and the WRAP, [^18^F]florbetapir PET was used for the ADC, and [^18^F]NAV4694 was used for TRIAD, PREVENT-AD and the AIBL; see Supplementary Table 18 for details.

### Tau positron emission tomography

Tau-PET was performed using [^18^F]flortaucipir for BioFINDER-1, MSCA, Knight ADRC and ADC cohorts using [^18^F]MK6240 for TRIAD, the AIBL and the WRAP and using [^18^F]RO948 for BioFINDER-2. Data were centrally processed at Lund University for BioFINDER-1, BioFINDER-2 and the ADC and locally processed for the other cohorts following previously described procedures (Supplementary Table 19). In line with our previous study^29^, we generated tau-PET standardized uptake value ratios (SUVRs) for a medial temporal lobe (unweighted average of the bilateral entorhinal cortex and the amygdala) and a neocortical temporal (weighted average of bilateral middle temporal and inferior temporal gyri) region of interest^3,53^.

### Plasma p-tau217

Plasma p-tau217 levels were measured using an immunoassay developed by Lilly Research Laboratories on a Meso Scale Discovery platform at Lund University for BioFINDER-1, BioFINDER-2, the ADC, the WRAP, PREVENT-AD and the Knight ADRC^54^ and at the Mayo Clinic for the MSCA^15^. For the AIBL and TRIAD, a plasma p-tau217+ assay developed by Janssen R&D on a Single Molecule Array (Simoa) HD-X platform was used^25^. The correspondence between the two plasma p-tau217 assays used in this study has been shown to be high in a direct head-to-head comparison study^55^.

### Clinical outcome measures

We used both continuous and binary measures of clinical progression. First, we examined cognitive trajectories using a sensitive composite measure specifically developed to detect cognitive changes in preclinical stages of AD (that is, the mPACC5 (refs. ^31,56^)). The mPACC5 consists of tests capturing episodic memory, executive function, semantic memory and global cognition^31^. Individual neuropsychological tests were *z* transformed using the baseline test scores of Aβ-negative participants in each cohort as the reference group and then averaged to obtain a composite *z* score. The composition of the mPACC5 for each cohort is described in Supplementary Table 20. Second, we examined progression from CU to MCI. MCI was established using the Petersen criteria^57^ and is defined as notable cognitive symptoms as assessed by a physician, in combination with cognitive impairment in one or multiple domains (for example, memory, executive functioning, attention, language), that is, below the normative range given an individual’s age, sex and educational background but not sufficiently severe to meet diagnostic criteria for dementia^58^.

### Statistics and reproducibility

All statistical analyses were performed in R version 4.3.1. Statistical significance for all models was set at *P* < 0.05, two sided, without correction for multiple comparisons. To enable pooling of the data across cohorts, we *z* transformed both tau-PET SUVRs and plasma p-tau217 concentrations based on the mean and the standard deviation of Aβ-negative participants within each cohort. We conducted two sets of main analyses, in which we examined (1) the individual and combined utility of tau biomarkers for their associations with longitudinal cognitive decline on the mPACC5 and with clinical progression from CU to MCI and (2) a sequential two-step approach for participant selection in a preclinical AD trial with a cognitive endpoint.

Within analyses assessing tau-PET and plasma p-tau217 versus mPACC5 change and progression to MCI, we used three different statistical models, including (1) only plasma p-tau217 concentration as a (continuous) predictor, (2) only a tau-PET measure as a (continuous) predictor (that is, tau-PET_MTL_ or tau-PET_NEO_ SUVR) or (3) including plasma p-tau217 and one of the tau-PET measures (that is, tau-PET_MTL_ or tau-PET_NEO_ SUVR) simultaneously as predictors. Additionally, we tested two basic models: model 1 including age, sex, years of education and cohort and model 2 consisting of the model 1 variables and *APOE* ε4 carriership. We calculated change in mPACC5 using linear mixed models with random time slopes and a random intercept using the lme4 package. Subsequently, these slopes were entered as the dependent variable in linear regression models with plasma p-tau217 and/or tau-PET as predictors, while adjusting for age, sex, education, cohort and *APOE* ε4 carriership. Differences in model performance were assessed by comparing differences in *R*^2^ by bootstrapping (1,000 repetitions with resample, boot package). Partial *R*^2^ values were calculated to assess the specific contribution of each predictor in the combined plasma p-tau217 and tau-PET models (rsq package).

Next, we examined progression from CU to MCI using Cox proportional hazard models, adjusting for age, sex, years of education, *APOE* ε4 carriership and cohort using the continuous variables as predictors. For individuals who progressed to MCI, we used the respective times at diagnostic progression to MCI for the analyses. To compare the predictive value of tau-PET versus plasma p-tau217 for diagnostic conversion, we compared the difference in AIC_c_ using bootstrapping procedures (1,000 repetitions with resample). In secondary analyses, all the above-mentioned analyses were repeated in Aβ-positive participants.

To assess potential differences between cohorts, we performed several sensitivity analyses. First, for the associations between the tau biomarkers and mPACC5 decline, we examined the effect sizes in all the previous models for each cohort independently (Figs. 1b and 2b). To that end, we applied the same linear regression models used in the main analyses to each cohort separately, without including cohort as a covariate. Similarly, we used Cox proportional hazard models in all cohorts independently to calculate the HRs and C-indices (Supplementary Table 11). Finally, we calculated the root mean square error of the predictions for each cohort when it was excluded from the training set. To do this, we calculated the cognition slopes for all cohorts combined, regressed out the cohort variable using a linear regression model and then trained a model (with age, sex and *APOE* as covariates) on all cohorts except one, subsequently predicting cognitive decline in the excluded cohort. We then calculated the root mean square error from the difference between the predicted data versus the observed data. We also performed additional analyses for both mPACC5 and progression to MCI when including only individuals with a minimum of 4 and 5 years of follow-up data available.

To derive optimal sample size reduction for a clinical trial in the two-step approach, we generated a data-driven estimate of the complementary value of tau-PET and plasma p-tau217 when implementing a sequential two-step approach (that is, plasma p-tau217 first, followed by tau-PET). First, we calculated the obtained sample size reduction when assuming 80% power to detect a 30% change in cognitive change (mPACC5) in a 4-year clinical trial (with annual repeated testing). Sample size was then defined by using different percentiles (75th, 50th and 25th) of the participants’ baseline plasma p-tau217 levels using the lmmpower function in the longpower package. The approach was repeated, selecting the 75th, 50th and 25th percentiles of the new participants’ tau-PET measures. We additionally tested a three-step approach in which, after initial plasma p-tau217 screening (step 1), amyloid-PET positivity (step 2) was incorporated, in turn followed by tau-PET (step 3). The percentage of participants needed compared to the entire study population and the plasma-selected sample are reported. Similar analyses were performed for progression to MC, using the ssizeCT.default function from the powerSurvEpi package. In this analysis, we aimed to detect a 30% reduction of events (that is, progression to MCI) at 80% power.

Next, we compared the characteristics of the sample included and excluded from the hypothetical clinical trial based on the different approaches presented. We focused on four combinations based on the participants selected on their plasma p-tau217 (step 1) and tau-PET_MTL_ (step 2) levels, that is, (1) a ‘liberal’ group comprising Q2–Q4 of plasma p-tau217 levels and Q2–Q4 of tau-PET of those selected by plasma, (2) a ‘moderate’ group consisting of individuals within Q3 and Q4 of plasma p-tau217 levels and Q3 and Q4 of tau-PET of those selected by plasma, (3) plasma p-tau217 Q4-only and (4) a ‘conservative’ group consisting of individuals within Q4 of plasma p-tau217 levels and Q4 of tau-PET of those selected by plasma. Based on these selection criteria, we compared mPACC slopes, the proportion of Aβ positivity, the final number of participants included and the proportion of ‘non-progressors’ between the plasma p-tau217 and tau-PET groups. We defined ‘non-progressors’ based on the mean and s.d. of the mPACC5 slope in the Aβ-negative CU participants from BioFINDER-2 (the largest cohort). By extracting the s.d. from the mean, we accounted for natural variation and/or fluctuations in (longitudinal) cognitive testing and potential learning effects. Based on this approach, every participant with a slope > −0.016 on the mPACC5 was classified as a ‘non-progressor’. As a sensitivity analysis, we repeated this analysis in BioFINDER-2 in which we had predefined cutoffs available for both plasma p-tau217 (0.499)^30^ and tau-PET (medial temporal lobe, 1.34 SUVR; temporal neocortex, 1.36 SUVR)^29^ and could classify individuals as ‘tau positive’ instead of using the quartile approach. Finally, we investigated the percentage of cost reductions of such approaches for participant selection in a hypothetical clinical trial using either mPACC5 or progression to MCI as the outcome measure assuming 80% power to detect a 30% change in mPACC5 or progression to MCI in a 4-year clinical trial (with annual repeated clinical assessments). We provided percentage cost reductions using different ratios of costs for tau-PET versus plasma p-tau217: 1:5 (that is, cost of one tau-PET scan resembling five plasma p-tau217 assessments), 1:10, 1:15 and 1:20.

### Reporting summary

Further information on research design is available in the Nature Portfolio Reporting Summary linked to this article.

## Supplementary information

Supplementary Information

Reporting Summary

## Data Availability

Due to the multicenter design of the study, access to individual participant data from each cohort will have to be made available through the PIs of the respective cohorts (that is, bf_executive@med.lu.se for the Swedish BioFINDER-1 and BioFINDER-2 studies, C.R.J. for the MCSA, C.R. for the AIBL, T.L.S.B. for the Knight ADRC, P.R.-N. for TRIAD, S.V. for the PREVENT-AD, S.J. for the WRAP and W.M.v.d.F. for the ADC). Generally, anonymized data can be shared by request from qualified academic investigators for the purpose of replicating procedures and results presented in the article, if data transfer is in agreement with data protection regulation at the institution and is approved by the local ethics review board.

## Code availability

The codes used for data analyses can be found at https://github.com/OssenKoppeLab.

## References

[1] Hansson, O. Biomarkers for neurodegenerative diseases. *Nat. Med.* **27**, 954–963 (2021). 34083813 10.1038/s41591-021-01382-x

[2] Strikwerda-Brown, C. et al. Association of elevated amyloid and tau positron emission tomography signal with near-term development of Alzheimer disease symptoms in older adults without cognitive impairment. *JAMA Neurol.* **79**, 975–985 (2022). 35907254 10.1001/jamaneurol.2022.2379 PMC9339146

[3] Ossenkoppele, R. et al. Discriminative accuracy of [^18^F]flortaucipir positron emission tomography for Alzheimer disease vs other neurodegenerative disorders. *JAMA* **320**, 1151–1162 (2018). 30326496 10.1001/jama.2018.12917 PMC6233630

[4] Jack, C. R. et al. The bivariate distribution of amyloid-β and tau: relationship with established neurocognitive clinical syndromes. *Brain* **142**, 3230–3242 (2019). 31501889 10.1093/brain/awz268 PMC6763736

[5] Ossenkoppele, R. et al. Accuracy of tau positron emission tomography as a prognostic marker in preclinical and prodromal Alzheimer disease: a head-to-head comparison against amyloid positron emission tomography and magnetic resonance imaging. *JAMA Neurol.* **78**, 961–971 (2021). 34180956 10.1001/jamaneurol.2021.1858 PMC8240013

[6] Pascoal, T. A. et al. ^18^F-MK-6240 PET for early and late detection of neurofibrillary tangles. *Brain* **143**, 2818–2830 (2020). 32671408 10.1093/brain/awaa180

[7] Leuzy, A. et al. Diagnostic performance of RO948 F 18 tau positron emission tomography in the differentiation of Alzheimer disease from other neurodegenerative disorders. *JAMA Neurol.* **77**, 955–965 (2020). 32391858 10.1001/jamaneurol.2020.0989 PMC7215644

[8] Ossenkoppele, R. & Hansson, O. Towards clinical application of tau PET tracers for diagnosing dementia due to Alzheimer’s disease. *Alzheimers Dement.* **17**, 1998–2008 (2021). 33984177 10.1002/alz.12356

[9] Hansson, O., Blennow, K., Zetterberg, H. & Dage, J. Blood biomarkers for Alzheimer’s disease in clinical practice and trials. *Nat. Aging* **3**, 506–519 (2023). 37202517 10.1038/s43587-023-00403-3 PMC10979350

[10] Dore, V. et al. Plasma p217+tau versus NAV4694 amyloid and MK6240 tau PET across the Alzheimer’s continuum. *Alzheimers Dement.* **14**, e12307 (2022). 10.1002/dad2.12307 PMC8984092 35415202

[11] Ashton, N. J. et al. Differential roles of Aβ_42/40_, p-tau231 and p-tau217 for Alzheimer’s trial selection and disease monitoring. *Nat. Med.* **28**, 2555–2562 (2022). 36456833 10.1038/s41591-022-02074-w PMC9800279

[12] Palmqvist, S. et al. Prediction of future Alzheimer’s disease dementia using plasma phospho-tau combined with other accessible measures. *Nat. Med.* **27**, 1034–1042 (2021). 34031605 10.1038/s41591-021-01348-z

[13] Barthelemy, N. R. et al. A soluble phosphorylated tau signature links tau, amyloid and the evolution of stages of dominantly inherited Alzheimer’s disease. *Nat. Med.* **26**, 398–407 (2020). 32161412 10.1038/s41591-020-0781-z PMC7309367

[14] Mila-Aloma, M. et al. Plasma p-tau231 and p-tau217 as state markers of amyloid-β pathology in preclinical Alzheimer’s disease. *Nat. Med.* **28**, 1797–1801 (2022). 35953717 10.1038/s41591-022-01925-w PMC9499867

[15] Mielke, M. M. et al. Performance of plasma phosphorylated tau 181 and 217 in the community. *Nat. Med.* **28**, 1398–1405 (2022). 35618838 10.1038/s41591-022-01822-2 PMC9329262

[16] Thijssen, E. H. et al. Plasma phosphorylated tau 217 and phosphorylated tau 181 as biomarkers in Alzheimer’s disease and frontotemporal lobar degeneration: a retrospective diagnostic performance study. *Lancet Neurol.* **20**, 739–752 (2021). 34418401 10.1016/S1474-4422(21)00214-3 PMC8711249

[17] Sperling, R. A. et al. Amyloid and tau prediction of cognitive and functional decline in unimpaired older individuals: longitudinal data from the A4 and LEARN studies. *J. Prev. Alzheimers Dis.* **11**, 802–813 (2024). 39044488 10.14283/jpad.2024.122 PMC11266444

[18] Palmqvist, S. et al. Blood biomarkers to detect Alzheimer disease in primary care and secondary care. *JAMA* **332**, 1245–1257 (2024). 10.1001/jama.2024.13855 PMC11284636 39068545

[19] Lista, S. et al. A critical appraisal of blood-based biomarkers for Alzheimer’s disease. *Ageing Res. Rev.* **96**, 102290 (2024). 38580173 10.1016/j.arr.2024.102290

[20] Ossenkoppele, R., van der Kant, R. & Hansson, O. Tau biomarkers in Alzheimer’s disease: towards implementation in clinical practice and trials. *Lancet Neurol.* **21**, 726–734 (2022). 35643092 10.1016/S1474-4422(22)00168-5

[21] Salvado, G. et al. Specific associations between plasma biomarkers and postmortem amyloid plaque and tau tangle loads. *EMBO Mol. Med.* **15**, e17123 (2023). 36912178 10.15252/emmm.202217123 PMC10165361

[22] Therriault, J. et al. Association of phosphorylated tau biomarkers with amyloid positron emission tomography vs tau positron emission tomography. *JAMA Neurol.* **80**, 188–199 (2023). 36508198 10.1001/jamaneurol.2022.4485 PMC9856704

[23] Smith, R. et al. Tau-PET is superior to phospho-tau when predicting cognitive decline in symptomatic AD patients. *Alzheimers Dement.* **19**, 2497–2507 (2023). 36516028 10.1002/alz.12875 PMC10264552

[24] Mundada, N. S. et al. Head-to-head comparison between plasma p-tau217 and flortaucipir-PET in amyloid-positive patients with cognitive impairment. *Alzheimers Res. Ther.* **15**, 157 (2023). 37740209 10.1186/s13195-023-01302-w PMC10517500

[25] Feizpour, A. et al. Two-year prognostic utility of plasma p217+tau across the Alzheimer’s continuum. *J. Prev. Alzheimers Dis.* **10**, 828–836 (2023). 37874105 10.14283/jpad.2023.83

[26] Sperling, R. A. et al. Trial of solanezumab in preclinical Alzheimer’s disease. *N. Engl. J. Med.* **389**, 1096–1107 (2023). 37458272 10.1056/NEJMoa2305032 PMC10559996

[27] van Dyck, C. H. et al. Lecanemab in early Alzheimer’s disease. *N. Engl. J. Med.* **388**, 9–21 (2023). 36449413 10.1056/NEJMoa2212948

[28] Sims, J. R. et al. Donanemab in early symptomatic Alzheimer disease: the TRAILBLAZER-ALZ 2 randomized clinical trial. *JAMA* **330**, 512–527 (2023). 37459141 10.1001/jama.2023.13239 PMC10352931

[29] Ossenkoppele, R. et al. Amyloid and tau PET-positive cognitively unimpaired individuals are at high risk for future cognitive decline. *Nat. Med.* **28**, 2381–2387 (2022). 36357681 10.1038/s41591-022-02049-x PMC9671808

[30] Mattsson-Carlgren, N. et al. Prediction of longitudinal cognitive decline in preclinical Alzheimer disease using plasma biomarkers. *JAMA Neurol.* **80**, 360–369 (2023). 36745413 10.1001/jamaneurol.2022.5272 PMC10087054

[31] Papp, K. V., Rentz, D. M., Orlovsky, I., Sperling, R. A. & Mormino, E. C. Optimizing the preclinical Alzheimer’s cognitive composite with semantic processing: the PACC5. *Alzheimers Dement.* **3**, 668–677 (2017). 10.1016/j.trci.2017.10.004 PMC5726754 29264389

[32] Jack, C. R. Jr. et al. Revised criteria for diagnosis and staging of Alzheimer’s disease: Alzheimer’s Association Workgroup. *Alzheimers Dement.* **20**, 5143–5169 (2024). 38934362 10.1002/alz.13859 PMC11350039

[33] Mattsson-Carlgren, N. et al. Longitudinal plasma p-tau217 is increased in early stages of Alzheimer’s disease. *Brain* **143**, 3234–3241 (2020). 33068398 10.1093/brain/awaa286 PMC7719022

[34] Montoliu-Gaya, L. et al. Optimal blood tau species for the detection of Alzheimer’s disease neuropathology: an immunoprecipitation mass spectrometry and autopsy study. *Acta Neuropathol.* **147**, 5 (2023). 38159140 10.1007/s00401-023-02660-3 PMC10757700

[35] Salvado, G. et al. Cerebral amyloid-β load is associated with neurodegeneration and gliosis: mediation by p-tau and interactions with risk factors early in the Alzheimer’s continuum. *Alzheimers Dement.* **17**, 788–800 (2021). 33663013 10.1002/alz.12245 PMC8252618

[36] Marquie, M. et al. Validating novel tau positron emission tomography tracer [F-18]-AV-1451 (T807) on postmortem brain tissue. *Ann. Neurol.* **78**, 787–800 (2015). 26344059 10.1002/ana.24517 PMC4900162

[37] Fleisher, A. S. et al. Positron emission tomography imaging with [^18^F]flortaucipir and postmortem assessment of Alzheimer disease neuropathologic changes. *JAMA Neurol.* **77**, 829–839 (2020). 32338734 10.1001/jamaneurol.2020.0528 PMC7186920

[38] Lowe, V. J. et al. Tau-positron emission tomography correlates with neuropathology findings. *Alzheimers Dement.* **16**, 561–571 (2020). 31784374 10.1016/j.jalz.2019.09.079 PMC7067654

[39] Cho, H. et al. Tau PET in Alzheimer disease and mild cognitive impairment. *Neurology* **87**, 375–383 (2016). 27358341 10.1212/WNL.0000000000002892

[40] Sperling, R. A. et al. The impact of amyloid-β and tau on prospective cognitive decline in older individuals. *Ann. Neurol.* **85**, 181–193 (2019). 30549303 10.1002/ana.25395 PMC6402593

[41] Biel, D. et al. Tau-PET and in vivo Braak-staging as prognostic markers of future cognitive decline in cognitively normal to demented individuals. *Alzheimers Res. Ther.* **13**, 137 (2021). 34384484 10.1186/s13195-021-00880-x PMC8361801

[42] Jack, C. R. Jr. et al. Associations of amyloid, tau, and neurodegeneration biomarker profiles with rates of memory decline among individuals without dementia. *JAMA* **321**, 2316–2325 (2019). 31211344 10.1001/jama.2019.7437 PMC6582267

[43] Ossenkoppele, R. et al. Associations between tau, Aβ, and cortical thickness with cognition in Alzheimer disease. *Neurology* **92**, e601–e612 (2019). 30626656 10.1212/WNL.0000000000006875 PMC6382060

[44] Brum, W. S. et al. A blood-based biomarker workflow for optimal tau-PET referral in memory clinic settings. *Nat. Commun.* **15**, 2311 (2024). 38486040 10.1038/s41467-024-46603-2 PMC10940585

[45] Mattsson-Carlgren, N. et al. Plasma biomarker strategy for selecting patients with Alzheimer disease for antiamyloid immunotherapies. *JAMA Neurol.* **81**, 69–78 (2024). 38048096 10.1001/jamaneurol.2023.4596 PMC10696515

[46] Villemagne, V. L. et al. CenTauR: toward a universal scale and masks for standardizing tau imaging studies. *Alzheimers Dement.* **15**, e12454 (2023). 10.1002/dad2.12454 PMC10326476 37424964

[47] Farrer, L. A. et al. Effects of age, sex, and ethnicity on the association between apolipoprotein E genotype and Alzheimer disease. A meta-analysis. *JAMA* **278**, 1349–1356 (1997). 9343467

[48] Yamazaki, Y., Zhao, N., Caulfield, T. R., Liu, C. C. & Bu, G. Apolipoprotein E and Alzheimer disease: pathobiology and targeting strategies. *Nat. Rev. Neurol.* **15**, 501–518 (2019). 31367008 10.1038/s41582-019-0228-7 PMC7055192

[49] Mattsson, N. et al. Prevalence of the apolipoprotein E ε4 allele in amyloid β positive subjects across the spectrum of Alzheimer’s disease. *Alzheimers Dement.* **14**, 913–924 (2018). 29601787 10.1016/j.jalz.2018.02.009

[50] Roberts, R. O. et al. The Mayo Clinic Study of Aging: design and sampling, participation, baseline measures and sample characteristics. *Neuroepidemiology* **30**, 58–69 (2008). 18259084 10.1159/000115751 PMC2821441

[51] Fowler, C. et al. Fifteen years of the Australian Imaging, Biomarkers and Lifestyle (AIBL) Study: progress and observations from 2,359 older adults spanning the spectrum from cognitive normality to Alzheimer’s disease. *J. Alzheimers Dis. Rep.* **5**, 443–468 (2021). 34368630 10.3233/ADR-210005 PMC8293663

[52] Slot, R. E. R. et al. Subjective Cognitive Impairment Cohort (SCIENCe): study design and first results. *Alzheimers Res. Ther.* **10**, 76 (2018). 30081935 10.1186/s13195-018-0390-y PMC6080529

[53] Jack, C. R. Jr. et al. Defining imaging biomarker cut points for brain aging and Alzheimer’s disease. *Alzheimers Dement.* **13**, 205–216 (2017). 27697430 10.1016/j.jalz.2016.08.005 PMC5344738

[54] Palmqvist, S. et al. Discriminative accuracy of plasma phospho-tau217 for Alzheimer disease vs other neurodegenerative disorders. *JAMA* **324**, 772–781 (2020). 32722745 10.1001/jama.2020.12134 PMC7388060

[55] Groot, C. et al. Diagnostic and prognostic performance to detect Alzheimer’s disease and clinical progression of a novel assay for plasma p-tau217. *Alzheimers Res. Ther.* **14**, 67 (2022). 35568889 10.1186/s13195-022-01005-8 PMC9107269

[56] Donohue, M. C. et al. The preclinical Alzheimer cognitive composite: measuring amyloid-related decline. *JAMA Neurol.* **71**, 961–970 (2014). 24886908 10.1001/jamaneurol.2014.803 PMC4439182

[57] Petersen, R. C. Mild cognitive impairment as a diagnostic entity. *J. Intern. Med.* **256**, 183–194 (2004). 15324362 10.1111/j.1365-2796.2004.01388.x

[58] Petersen, R. C. et al. Practice guideline update summary: mild cognitive impairment: Report of the Guideline Development, Dissemination, and Implementation Subcommittee of the American Academy of Neurology. *Neurology* **90**, 126–135 (2018). 29282327 10.1212/WNL.0000000000004826 PMC5772157

