# Supplementary material for: Plasma p-tau217 and tau-PET predict future cognitive decline among cognitively unimpaired individuals: implications for clinical trials
Source: Nat Aging. 2025 Mar 28;5(5):883–96. doi: 10.1038/s43587-025-00835-z (PMC12092243; doi:10.1038/s43587-025-00835-z)
Supplement: Supplementary file 1 — Supplementary Information [file 43587_2025_835_MOESM1_ESM.pdf]

nature aging

Supplementary information

https://doi.org/10.1038/s43587-025-00835-z

Plasma p-tau217 and tau-PET predict future cognitive decline among cognitively unimpaired individuals: implications for clinical trials

In the format provided by the authors and unedited

SUPPLEMENTARY DATA CONTENT

Table/Figure	Title	Page
Table 1	Participant characteristics by cohort (all participants)	2-10
Table 2	Performance indicators of models predicting mPACC5 decline	11
Table 3	Comparison of different models predicting mPACC5 decline	12
Table 4	Variance explained by different models predicting mPACC5 decline	13
Table 5	RMSE of different models predicting mPACC5 decline by cohort	14
Figure 1	Simple and combined mPACC5 models by cohort	15
Figure 2	mPACC5 models with longer follow-up data	16
Table 6	Performance indicators mPACC5 in Aβ+ participants	17
Table 7	Comparison of different models mPACC5 in Aβ+ participants	18
Table 8	Variance explained mPACC5 in Aβ+ participants	19
Table 9	Performance of different models predicting MCI clinical progression	20
Table 10	Comparison (p-values) predicting MCI across all participants	21
Table 11	C-index predicting MCI across all participants	22
Figure 3	Effect sizes combined plasma/PET models for predicting MCI by cohort	23
Figure 4	Associations between plasma/PET biomarkers and clinical progression to MCI in individuals with longer follow-up	24
Table 12	Performance of different models predicting clinical progression to MCI in Aβ+ participants	25
Table 13	Comparison (p-values) of different models predicting clinical progression to MCI in Aβ+ participants	26
Figure 5	Two-step approach for clinical trials using clinical progression to MCI using Tau-PET_{NEO}	27
Figure 6	A three-step screening with MCI progression as outcome measure	28
Figure 7	Characterization of different plasma p-tau217/Tau-PET_{NEO} groups	29
Table 14	Sample size reductions in a clinical trial following a two-step approach	30
Table 15	Combined plasma p-tau217 and Tau-PET_{MTL} group characterizations: Aβ status and clinical outcomes	31
Table 16	Combined plasma p-tau217 and Tau-PET_{MTL} group characterizations: Demographic information	32
Figure 8	Relevant trial outcomes when using predefined cut-offs in BioFINDER-2	33
Figure 9	Projected costs that could be saved in a hypothetical trial with mPACC5 as an endpoint	34
Figure 10	Projected costs that could be saved in a hypothetical trial with clinical progression to MCI as an endpoint	33
Table 17	Cohort descriptions	34-36
Table 18	Methods to determine Amyloid PET status by cohort	37-38
Table 19	Methods to determine Tau PET status	39
Table 20	Composition of the mPACC5 for each cohort	41
	References	43-44

Supplementary Table 1. Participant characteristics by cohort (all participants)

ADC		
	**All participants**	**Aβ+ participants only**
N	44	17
Age, years	65.0±7.5	66.4±6.3
Sex, % female	45.5%	47.1%
Education, years	12.1±2.7	12.2±2.8
MMSE score	28.8±1.3	28.4±1.3
*APOE e*4 status, % carriers	38.6%	64.7%
Aβ-status, % positive	38.6%	100%
Follow-up duration, years	4.6±1.8	3.8±1.6
Follow-up visits, median (range)	5 (2-8)	5 (3-7)
Plasma p-tau217, z-score	0.62±1.4	1.59±1.28
Tau-PET_{MTL}, z-score	0.71±1.75	1.84±2.10
Tau-PET_{NEO}, z-score	0.81±2.50	2.10±3.51
mPACC5, baseline score	-0.19±0.74	-0.50±0.61
mPACC5, annual change	-0.065±0.084	-0.161±0.148
% Progression to MCI	13.6%	35.3%

AIBL		
	**All participants**	**Aβ+ participants only**
N	180	34
Age, years	74.7±5.3	77.5±6.4
Sex, % female	52.8%	58.8%
Education, years	12.7±2.7	11.5±2.9
MMSE score	28.5 ±1.4	27.9±1.6
*APOE e*4 status, % carriers	29.4%	58.8%
Aβ-status, % positive	18.9%	100%
Follow-up duration, years	3.2±0.8	2.9±0.9
Follow-up visits, median (range)	3 (2-4)	3 (2-4)
Plasma p-tau217, z-score	0.21±0.99	0.95±0.88
Tau-PET_{MTL}, z-score	0.28±1.17	1.57±1.30
Tau-PET_{NEO}, z-score	0.27±1.43	1.27±2.36
mPACC5, baseline score	-0.02±0.71	-0.27±0.80
mPACC5, annual change	-0.045±0.068	-0.130±0.142
% Progression to MCI	3.9%	8.8%

BioFINDER-1		
	**All participants**	**Aβ+ participants only**
N	40	12
Age, years	73.5±7.0	74.2±5.9
Sex, % female	52.5%	58.3%
Education, years	11.9±3.7	10.7±3.0
MMSE score	28.6±1.3	28.3±1.7
*APOE e*4 status, % carriers	52.5%	75.0%
Aβ-status, % positive	30.0%	100%
Follow-up duration, years	3.4±0.75	3.3±0.9
Follow-up visits, median (range)	2 (2-5)	2 (2-5)
Plasma p-tau217, z-score	0.13±1.23	0.53±1.70
Tau-PET_{MTL}, z-score	0.36±1.69	1.40±2.48
Tau-PET_{NEO}, z-score	0.41±1.91	1.45±2.99
mPACC5, baseline score	0.04±0.74	-0.22±0.87
mPACC5, annual change	-0.042±0.059	-0.082±0.095
% Progression to MCI	12.5%	41.7%

BioFINDER-2		
	**All participants**	**Aβ+ participants only**
N	481	137
Age, years	65.0±11.4	70.1±9.1
Sex, % female	52.4%	49.6%
Education, years	12.8±3.5	12.8±3.8
MMSE score	28.9±1.3	28.7±1.4
*APOE e*4 status, % carriers	48.2%	71.5%
Aβ-status, % positive	28.5%	100%
Follow-up duration, years	3.0±1.1	3.0±1.2
Follow-up visits, median (range)	3 (2-6)	3 (2-6)
Plasma p-tau217, z-score	0.48±1.36	1.78±1.23
Tau-PET_{MTL}, z-score	0.26±1.58	1.53±2.05
Tau-PET_{NEO}, z-score	0.13±1.66	0.99±2.52
mPACC5, baseline score	0.17±0.78	-0.11±0.81
mPACC5, annual change	-0.034±0.088	-0.113±0.174
% Progression to MCI	11.0%	26.3%

Knight ADRC		
	**All participants**	**Aβ+ participants only**
N	109	34
Age, years	70.2±6.4	70.6±6.3
Sex, % female	53.2%	61.8%
Education, years	16.3±2.3	16.6±2.3
MMSE score	29.3±1.1	29.4±1.1
*APOE e*4 status, % carriers	29.4%	35.3%
Aβ-status, % positive	31.2%	100%
Follow-up duration, years	3.9±1.7	3.6±1.5
Follow-up visits, median (range)	4 (2-8)	4 (2-8)
Plasma p-tau217, z-score	0.71±1.79	2.10±2.23
Tau-PET_{MTL}, z-score	0.27±1.21	0.85±1.39
Tau-PET_{NEO}, z-score	0.31±1.53	0.94±2.17
mPACC5, baseline score	-0.08±0.68	-0.13±0.76
mPACC5, annual change	-0.050±0.083	-0.138±0.144
% Progression to MCI	11.9%	20.6%

MCSA		
	**All participants**	**Aβ+ participants only**
N	363	108
Age, years	68.3±12.0)	76.4±7.9
Sex, % female	45.7%	53.7%
Education, years	15.1±2.3	14.7±2.5
MMSE score	28.8±1.0	28.5±1.2
*APOE e*4 status, % carriers	29.2%	47.2%
Aβ-status, % positive	108 (29.8%)	100%
Follow-up duration, years	5.6±2.1	4.9±2.2
Follow-up visits, median (range)	5 (2-7)	5 (2-7)
Plasma p-tau217, z-score	0.42±1.29	1.34±1.40
Tau-PET_{MTL}, z-score	0.17±1.18	0.76±1.41
Tau-PET_{NEO}, z-score	0.06±1.09	0.47±1.20
mPACC5, baseline score	-0.01±0.75	-0.42±0.67
mPACC5, annual change	-0.038±0.053	-0.102±0.084
% Progression to MCI	11.0%	25.0%

PREVENT-AD		
	**All participants**	**Aβ+ participants only**
N	51	19
Age, years	68.4±4.9	69.9±5.3
Sex, % female	70.6%	68.4%
Education, years	15.2±3.39	14.5±3.2
MMSE score	28.5±1.5	27.7±2.5
*APOE e*4 status, % carriers	47.1%	63.2%
Aβ-status, % positive	37.3%	100%
Follow-up duration, years	3.5±1.9	3.69±2.0
Follow-up visits, median (range)	4 (1-6)	4 (1-5)
Plasma p-tau217, z-score	0.95±1.92	2.43±2.23
Tau-PET$_{MTL}$, z-score	0.59±1.33	1.27±1.60
Tau-PET$_{NEO}$, z-score	0.20±1.36	0.76±1.90
mPACC5, baseline score	-0.18±0.87	-0.41±0.95
mPACC5, annual change	-0.078±0.078	-0.101±0.109
% Progression to MCI	29.4%	52.6%

TRIAD		
	**All participants**	**Aβ+ participants only**
N	124	27
Age, years	71.4±5.8	74.2±4.8
Sex, % female	66.9%	74.1%
Education, years	15.7±3.6	14.1±3.2
MMSE score	29.2±0.9	29.0±1.1
*APOE e*4 status, % carriers	22.6%	25.9%
Aβ-status, % positive	21.8%	100%
Follow-up duration, years	2.4±0.7	2.2±0.5
Follow-up visits, median (range)	3 (2-4)	3 (2-4)
Plasma p-tau217, z-score	0.31±1.20	1.61±0.98
Tau-PET_{MTL}, z-score	0.36±1.38	1.55±1.88
Tau-PET_{NEO}, z-score	0.15±1.12	0.60±1.28
mPACC5, baseline score	-0.02±0.75	-0.083±0.81
mPACC5, annual change	-0.053±0.070	-0.107±0.160
% Progression to MCI	13.7%	33.3%

WRAP		
	**All participants**	**Aβ+ participants only**
N	82	20
Age, years	68.1±5.9	70.5±4.5
Sex, % female	58.5%	50.0%
Education, years	16.5±2.1	17.1±2.1
MMSE score	29.4±0.9	28.9±1.3
*APOE e*4 status, % carriers	41.5%	55.0%
Aβ-status, % positive	24.4%	100%
Follow-up duration, years	3.0±1.1	2.68±0.79
Follow-up visits, median (range)	2 (2-3)	2 (2-3)
Plasma p-tau217, z-score	0.70±1.66	2.82±1.43
Tau-PET$_{MTL}$, z-score	0.43±1.79	1.90±2.66
Tau-PET$_{NEO}$, z-score	0.25±1.53	0.93±2.52
mPACC5, baseline score	0.01±0.74	-0.22±0.88
mPACC5, annual change	-0.053±0.083	-0.121±0.140
% Progression to MCI	7.3%	25.0%

Supplementary Table 2. Performance indicators of models predicting decline on the mPACC5 across all participants

**Model**	**plasma p-tau217 β_{std} [95%CI]**	**p plasma p-tau217**	**Tau-PET β_{std} [95%CI]**	**p Tau-PET**	**R^2**	**AICc**
**All participants**						
Basic without *APOE*	-	-	-	-	0.23	7524.3
Basic with *APOE*	-	-	-	-	0.24	7507.5
Plasma p-tau217	-0.08 [-0.10, -0.07]	<0.001	-	-	0.33	7239.1
Tau-PET_{MTL}	-	-	-0.08 [-0.09, -0.06]	<0.001	0.34	7232.8
Tau-PET_{NEO}	-	-	-0.07 [-0.08, -0.06]	<0.001	0.33	7252.6
Plasma p-tau217 & Tau-PET_{MTL}	-0.06 [-0.08, -0.05]	<0.001	-0.06 [-0.07, -0.04]	<0.001	0.35	7146.6
Plasma p-tau217 & Tau-PET_{NEO}	-0.07 [-0.08, -0.06]	<0.001	-0.05 [-0.07, -0.04]	<0.001	0.35	7149.6

Standardized β-coefficients, R^2 and corrected AIC derived from linear regression models testing the association between the tau biomarker and annual change on the mPACC5, while adjusting for age, sex, education, cohort and *APOE* ε4 status, across all participants.

Supplementary Table 3. Comparison of different models predicting cognitive decline on the mPACC5 across all participants

**P-values**	Basic without APOE	Basic with APOE	Plasma p-tau217	Tau-PET$_{MTL}$	Tau-PET$_{NEO}$	Plasma p-tau217 & Tau-PET$_{MTL}$	Plasma p-tau217 & Tau-PET$_{NEO}$
**All Participants**							
Basic without *APOE*	1	0.032	<0.001	<0.001	<0.001	<0.001	<0.001
Basic with *APOE*		1	<0.001	<0.001	0.001	<0.001	<0.001
Plasma p-tau217			1	0.653	0.752	<0.001	0.001
Tau-PET$_{MTL}$				1	0.356	<0.001	0.028
Tau-PET$_{NEO}$					1	0.001	<0.001
Plasma p-tau217 & Tau-PET$_{MTL}$						1	0.757
Plasma p-tau217 & Tau-PET$_{NEO}$							1

Numbers represent p-values derived from linear regression models comparing different models (rows vs columns).

Supplementary Table 4. Variance explained by different models predicting cognitive decline on the mPACC5 across all participants

**Model**	**Total R^2**	**Partial R^2 covariates**	**Partial R^2 plasma p-tau217**	**Partial R^2 Tau-PET**	**Partial R^2 shared**
**All participants**					
Basic without *APOE*	0.23	0.25	-	-	0.00
Basic with *APOE*	0.24	0.27	-	-	0.00
Plasma p-tau217	0.32	0.19	0.10	-	0.03
Tau-PET_{MTL}	0.32	0.20	-	0.11	0.02
Tau-PET_{NEO}	0.31	0.24	-	0.09	0.00
Plasma p-tau217 & Tau-PET_{MTL}	0.36	0.16	0.05	0.06	0.08
Plasma p-tau217 & Tau-PET_{NEO}	0.35	0.19	0.06	0.06	0.05

Numbers represent R^2's derived from linear regression models

Supplementary Table 5. Performance indicator (RMSE) [95% CI] of different models predicting decline on the mPACC5 by cohort

**Cohort**	**N**	**Basic without APOE**	**Basic with APOE**	**Plasma p-tau217**	**Tau-PET$_{MTL}$**	**Tau-PET $_{NEO}$**	**Plasma p-tau217 & Tau-PET$_{MTL}$**	**Plasma p-tau217 & Tau-PET$_{NEO}$**
ADC	44	0.081 [0.081,0.082]	0.079 [0.078,0.080]	0.067 [0.064,0.069]	0.061 [0.057,0.063]	0.068 [0.066,0.069]	0.057 [0.054,0.059]	0.062 [0.060,0.063]
AIBL	179	0.069 [0.067,0.071]	0.069 [0.067,0.071]	0.066 [0.064,0.067]	0.066 [0.064,0.068]	0.064 [0.062,0.065]	0.064 [0.062,0.065]	0.062 [0.061,0.064]
BioFINDER-1	37	0.050 [0.047,0.053]	0.051 [0.047,0.054]	0.049 [0.046,0.052]	0.049 [0.044,0.053]	0.045 [0.041,0.049]	0.049 [0.045,0.053]	0.046 [0.043,0.049]
BioFINDER-2	481	0.081 [0.080,0.082]	0.080 [0.079,0.081]	0.076 [0.075,0.077]	0.076 [0.075,0.077]	0.075 [0.074,0.076]	0.074 [0.073,0.074]	0.074 [0.072,0.074]
Knight ADRC	58	0.080 [0.079,0.080]	0.079 [0.078,0.080]	0.067 [0.066,0.067]	0.070 [0.068,0.071]	0.075 [0.074,0.075]	0.064 [0.063,0.065]	0.067 [0.066,0.068]
MCSA	362	0.074 [0.073,0.074]	0.073 [0.072,0.073]	0.068 [0.067,0.068]	0.073 [0.072,0.073]	0.071 [0.070,0.072]	0.069 [0.067,0.069]	0.068 [0.066,0.068]
PREVENT-AD	44	0.076 [0.076,0.077]	0.073 [0.072,0.075]	0.068 [0.066,0.069]	0.072 [0.070,0.073]	0.065 [0.063,0.067]	0.066 [0.064,0.067]	0.062 [0.060,0.064]
TRIAD	113	0.060 [0.059,0.060]	0.061 [0.059,0.061]	0.064 [0.062,0.066]	0.059 [0.058,0.060]	0.059 [0.057,0.060]	0.062 [0.060,0.063]	0.062 [0.060,0.063]
WRAP	58	0.071 [0.069,0.072]	0.071 [0.069,0.072]	0.068 [0.066,0.069]	0.064 [0.062,0.066]	0.065 [0.063,0.066]	0.063 [0.061,0.064]	0.065 [0.063,0.066]

Numbers represent Root-mean-square deviation (RMSE) derived from linear regression models

Supplementary Figure 1. Effect sizes of combined plasma and PET models for predicting mPACC5 decline by cohort

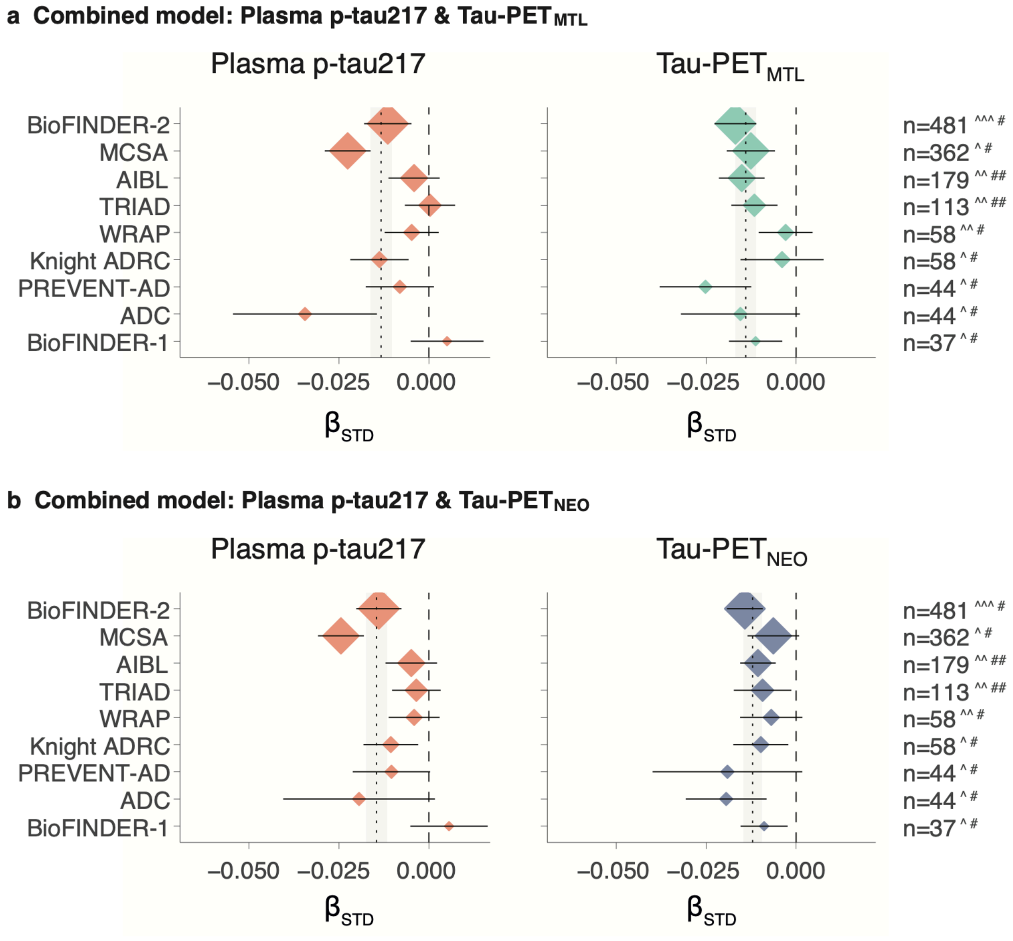

Effect sizes and 95%CI (expressed as standardized beta's) for predicting longitudinal changes on the mPACC5 in each of the cohorts. Size of the rhomboid relates to the sample size of each cohort. The vertical dashed line represents standardized beta = 0, while the vertical dotted line represent the average standardized beta across all cohorts with the 95% CI indicated in gray. Standardized β-coefficients shown here relate to the tau biomarker as a continuous variable.
^ [^{18}F]flortaucipir PET, ^^ [^{18}F]MK6240 PET, ^^^ [^{18}F]RO948 PET; # Lilly plasma p-tau217 immunoassay, ## Janssen plasma p-tau217+ assay.

Supplementary Figure 2. Associations between plasma/PET biomarkers and mPACC5 decline in individuals with longer follow-up

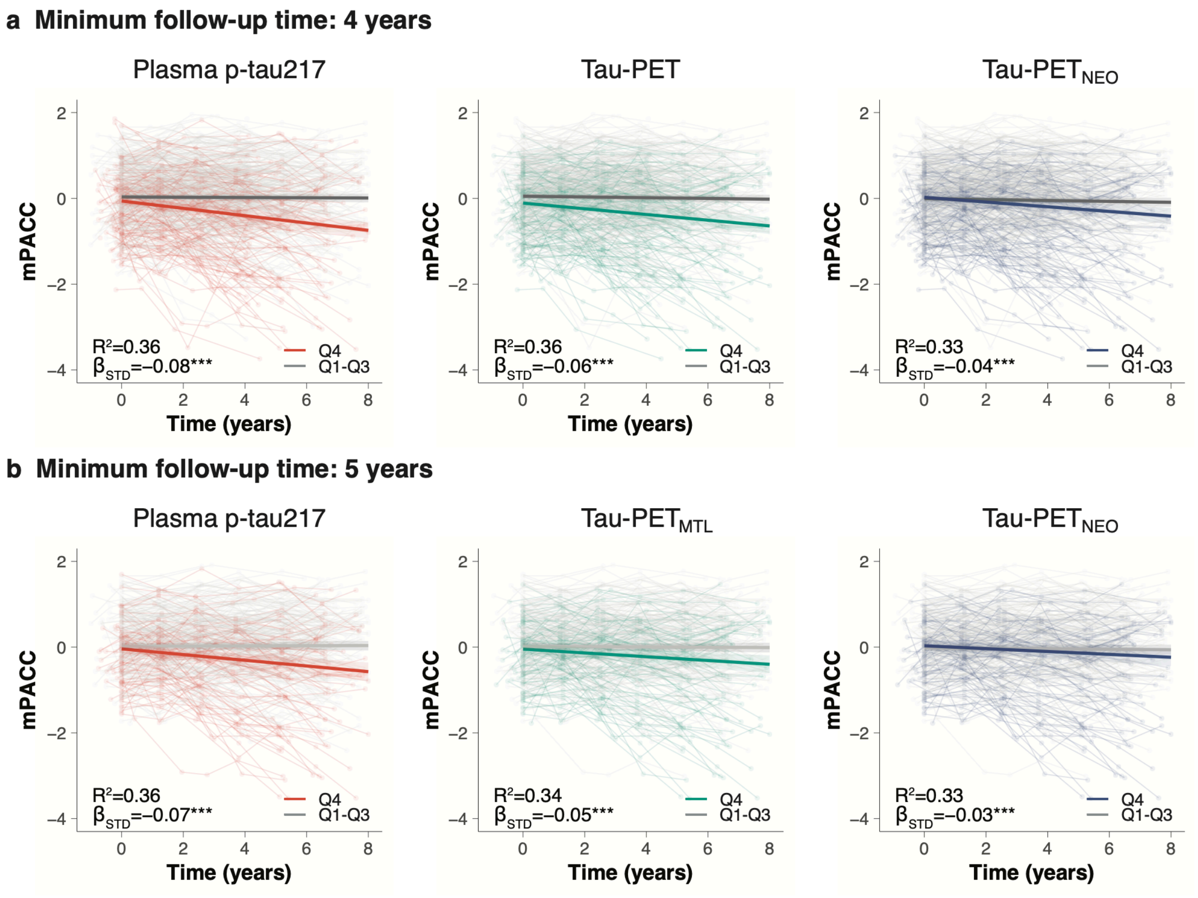

We included individuals that had at least 4 (**a**) or 5 (**b**) years of follow-up, respectively. Classification into quartiles was done for visualization purposes only. Standardized β-coefficients and R^2 statistics relate to the tau biomarker as a continuous variable. The shadow area indicates the 95% confidence interval derived from linear regression models. ***p<0.001.

Supplementary Table 6. Performance indicators of models predicting decline on the mPACC5 in Aβ+ participants

**Model**	**plasma p-tau217 β_{std} [95%CI]**	**p plasma p-tau217**	**Tau-PET β_{std} [95%CI]**	**p Tau-PET**	**R^2**	**AICc**
**Aβ+ participants**						
Basic without *APOE*	-	-	-	-	0.20	2535.5
Basic with *APOE*	-	-	-	-	0.20	2536.6
Plasma p-tau217	-0.13 [-0.16, -0.10]	<0.001	-	-	0.33	2444.9
Tau-PET$_{MTL}$	-	-	-0.11 [-0.14, -0.09]	<0.001	0.37	2420.0
Tau-PET$_{NEO}$	-	-	-0.11 [-0.13, -0.09]	<0.001	0.36	2405.6
Plasma p-tau217 & Tau-PET$_{MTL}$	-0.09 [-0.12, -0.06]	<0.001	-0.09 [-0.11, -0.06]	<0.001	0.39	2389.7
Plasma p-tau217 & Tau-PET$_{NEO}$	-0.09 [-0.12, -0.06]	<0.001	-0.08 [-0.10, -0.06]	<0.001	0.38	2377.7

The effect sizes are derived from linear regression models.

Supplementary Table 7. Comparison of different models predicting cognitive decline on the mPACC5 in Aβ+ participants

**P-values**	Basic without APOE	Basic with APOE	Plasma p-tau217	Tau-PET$_{MTL}$	Tau-PET$_{NEO}$	Plasma p-tau217 & Tau-PET$_{MTL}$	Plasma p-tau217 & Tau-PET$_{NEO}$
**Aβ+ participants**							
Basic without *APOE*	1	0.661	<0.001	<0.001	<0.001	<0.001	<0.001
Basic with *APOE*		1	<0.001	<0.001	<0.001	<0.001	<0.001
Plasma p-tau217			1	0.257	0.241	<0.001	0.004
Tau-PET$_{MTL}$				1	0.725	0.005	0.079
Tau-PET$_{NEO}$					1	0.306	0.018
Plasma p-tau217 & Tau-PET$_{MTL}$						1	0.759
Plasma p-tau217 & Tau-PET$_{NEO}$							1

Numbers represent p-values derived from linear regression models comparing different models (rows vs columns).

Supplementary Table 8. Variance explained by different models predicting cognitive decline on the mPACC5 in Aβ+ participants

Model	Total R^2	Partial R^2 covariates	Partial R^2 plasma p-tau217	Partial R^2 Tau-PET	Partial R^2 shared
**Aβ+ participants**					
Basic without *APOE*	0.23	0.25	-	-	0.00
Basic with *APOE*	0.24	0.27	-	-	0.00
Plasma p-tau217	0.32	0.19	0.10	-	0.03
Tau-PET_{MTL}	0.32	0.20	-	0.11	0.02
Tau-PET_{NEO}	0.31	0.24	-	0.09	0.00
Plasma p-tau217 & Tau-PET_{MTL}	0.36	0.16	0.05	0.06	0.08
Plasma p-tau217 & Tau-PET_{NEO}	0.35	0.19	0.06	0.06	0.05

Numbers represent R^2's derived from linear regression models

Supplementary Table 9. Performance of different models predicting clinical progression to MCI across all participants

Model	N non-progressor	N progressor	HR plasma p-tau217	p plasma p-tau217	HR Tau-PET	p Tau-PET	C-index	AICc
**All participants**								
Basic without *APOE*	1264	162	-	-	-	-	0.75	2054
Basic with *APOE*	1264	162	-	-	-	-	0.77	2038
Plasma p-tau217	1264	162	1.57 [1.43, 1.72]	<0.001	-	-	0.83	1960
Tau-PET_{MTL}	1264	162	-	-	1.61 [1.48, 1.76]	<0.001	0.83	1937
Tau-PET_{NEO}	1264	162	-	-	1.43 [1.34, 1.52]	<0.001	0.81	1967
Plasma p-tau217 & Tau-PET_{MTL}	1264	162	1.37 [1.23, 1.53]	<0.001	1.43 [1.30, 1.57]	<0.001	0.84	1910
Plasma p-tau217 & Tau-PET_{NEO}	1264	162	1.42 [1.28, 1.57]	<0.001	1.27 [1.18, 1.37]	<0.001	0.83	1927

Hazard ratios, C-index and corrected AIC derived from Cox proportional hazard models testing the association between the tau biomarker and progression to MCI, while adjusting for age, sex, education, cohort and *APOE* ε4 status, across all participants.

Supplementary Table 10. Comparison (p-values) of different models predicting clinical progression to MCI across all participants

**P-values**	Basic without *APOE*	Basic with *APOE*	Plasma p-tau217	Tau-PET$_{MTL}$	Tau-PET$_{NEO}$	Plasma p-tau217 & Tau-PET$_{MTL}$	Plasma p-tau217 & Tau-PET$_{NEO}$
**All Participants**							
Basic without *APOE*	1	0.055	0.002	<0.001	0.001	<0.001	<0.001
Basic with *APOE*		1	0.001	<0.001	<0,001	<0.001	<0.001
Plasma p-tau217			1	0.322	0.750	<0.001	0.009
Tau-PET$_{MTL}$				1	0.059	0.017	0.597
Tau-PET$_{NEO}$					1	0.002	0.004
Plasma p-tau217 & Tau-PET$_{MTL}$						1	0.122
Plasma p-tau217 & Tau-PET$_{NEO}$							1

Numbers represent p-values derived from Cox proportional hazard models comparing different models (rows vs columns).

Supplementary Table 11. C-index of different models predicting clinical progression to MCI across all participants

Cohort	N	Basic without *APOE*	Basic with *APOE*	Plasma p-tau217	Tau-PET$_{MTL}$	Tau-PET$_{NEO}$	Plasma p-tau217 & Tau-PET$_{MTL}$	Plasma p-tau217 & Tau-PET$_{NEO}$
ADC	44	0.711 [0.677,0.790]	0.786 [0.741,0.848]	0.934 [0.911,0.968]	0.912 [0.893,0.940]	0.952 [0.941,0.971]	0.947 [0.931,0.972]	0.960 [0.948,0.974]
AIBL	179	0.637 [0.587,0.679]	0.662 [0.628,0.707]	0.682 [0.649,0.726]	0.656 [0.628,0.687]	0.708 [0.689,0.738]	0.673 [0.642,0.716]	0.705 [0.673,0.737]
BioFINDER-1	38	0.612 [0.515,0.707]	0.785 [0.696,0.883]	0.738 [0.669,0.815]	0.877 [0.847,0.965]	0.854 [0.812,0.898]	0.792 [0.741,0.849]	0.815 [0.775,0.862]
BioFINDER-2	441	0.714 [0.705,0.727]	0.731 [0.725,0.748]	0.828 [0.820,0.845]	0.806 [0.796,0.827]	0.826 [0.818,0.842]	0.836 [0.826,0.854]	0.838 [0.829,0.854]
Knight ADRC	109	0.822 [0.792,0.863]	0.748 [0.708,0.791]	0.827 [0.802,0.856]	0.740 [0.686,0.801]	0.703 [0.658,0.742]	0.793 [0.762,0.819]	0.784 [0.749,0.821]
MCSA	363	0.754 [0.748,0.766]	0.776 [0.767,0.792]	0.829 [0.823,0.842]	0.793 [0.786,0.806]	0.826 [0.819,0.841]	0.830 [0.822,0.841]	0.852 [0.846,0.866]
PREVENT-AD	46	0.711 [0.671,0.760]	0.717 [0.698,0.751]	0.754 [0.744,0.772]	0.784 [0.765,0.825]	0.838 [0.836,0.870]	0.762 [0.746,0.782]	0.784 [0.772,0.802]
TRIAD	124	0.618 [0.600,0.642]	0.640 [0.623,0.672]	0.631 [0.610,0.644]	0.667 [0.643,0.694]	0.595 [0.568,0.619]	0.664 [0.645,0.685]	0.625 [0.600,0.639]
WRAP	82	0.627 [0.595,0.635]	0.614 [0.577,0.650]	0.910 [0.889,0.942]	0.885 [0.857,0.910]	0.856 [0.827,0.894]	0.923 [0.899,0.946]	0.904 [0.874,0.928]

The presented C-index is derived from Cox proportional hazard models.

Supplementary Figure 3. Effect sizes of combined plasma and PET models for predicting clinical progression to MCI by cohort

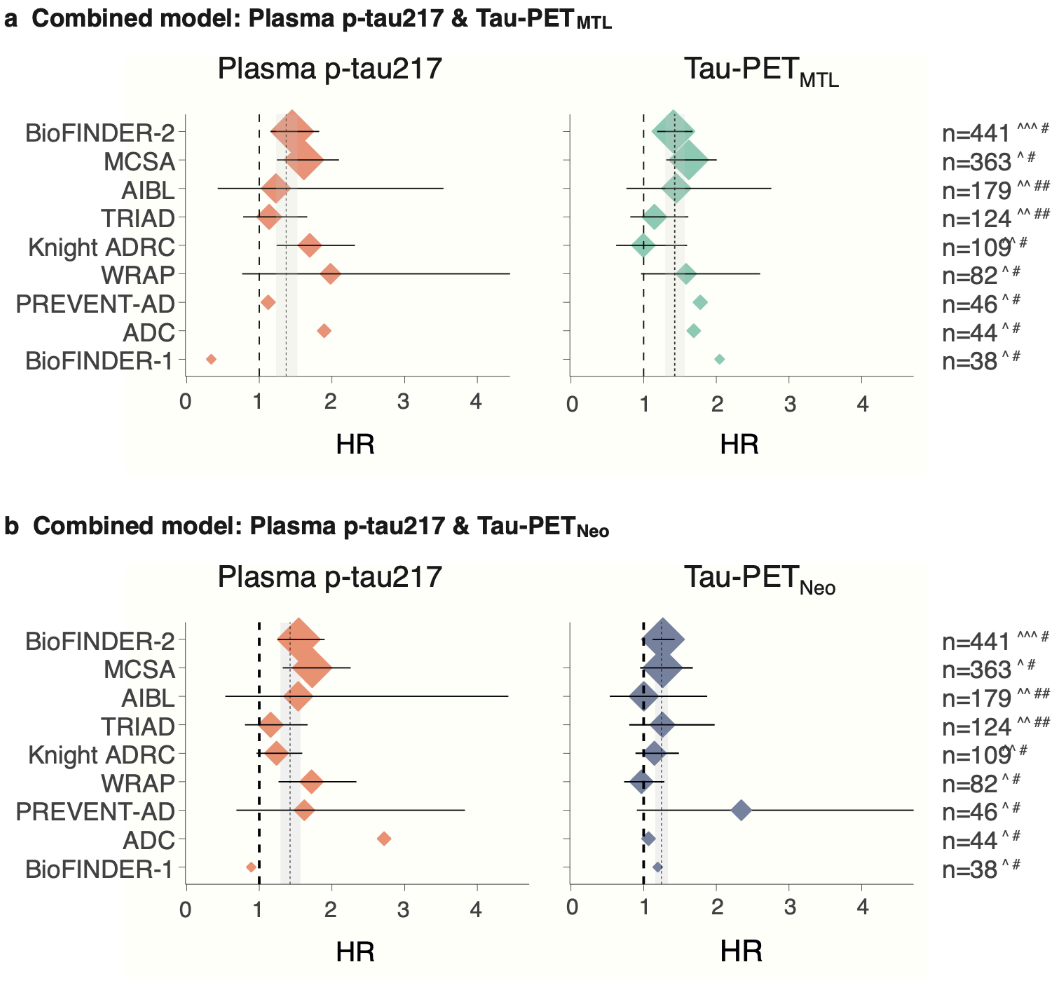

Hazard ratios and 95%CI for predicting progression to MCI in each of the cohorts. The vertical dashed line represents HR=1, while the vertical dotted line represent the average hazard ratio across all cohorts with the 95% CI indicated in gray. HRs shown here relate to the tau biomarker as a continuous variable.
^ [^{18}F]flortaucipir PET, ^^ [^{18}F]MK6240 PET, ^^^ [^{18}F]RO948 PET; # Lilly plasma p-tau217 immunoassay, ## Janssen plasma p-tau217+ assay.

Supplementary Figure 4. Associations between plasma/PET biomarkers and clinical progression to MCI in individuals with longer follow-up

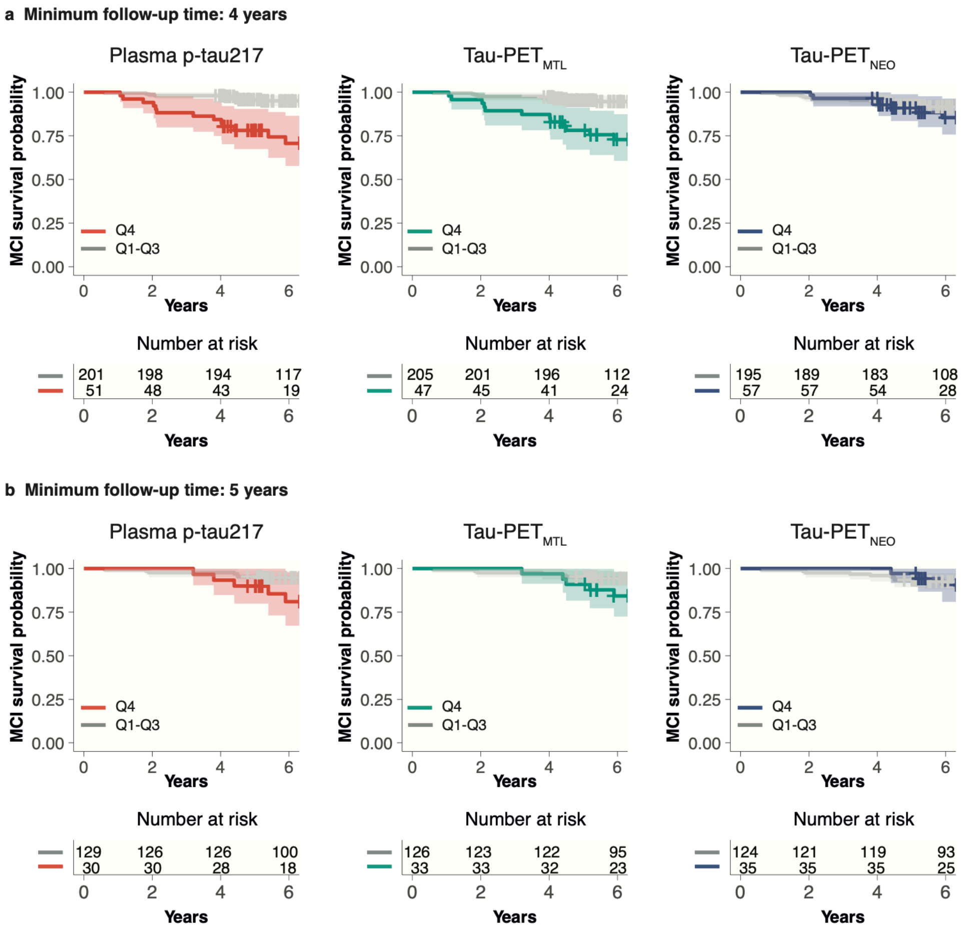

Survival curves for progression to mild cognitive impairment (Quartile 1-3 vs Quartile 4) across all participants with at least 4 (**a**) or 5 (**b**) years of follow-up data, including a Table showing the total number of participants available at each time point. The shadow area indicates the 95% confidence interval around the mean.

Supplementary Table 12. Performance of different models predicting clinical progression to MCI in Aβ+ participants

**Model**	**N non-progressor**	**N progressor**	**HR plasma p-tau217**	**p plasma p-tau217**	**HR Tau-PET**	**p Tau-PET**	**C-index**	**AICc**
**Aβ+ participants**								
Basic without *APOE*	288	108	-	-	-	-	0.67	1139
Basic with *APOE*	288	108	-	-	-	-	0.67	1136
Plasma p-tau217	288	108	1.58 [1.38, 1.80]	<0.001	-	-	0.75	1094
Tau-PET_{MTL}	288	108	-	-	1.53 [1.39, 1.70]	<0.001	0.78	1072
Tau-PET_{NEO}	288	108	-	-	1.34 [1.25, 1.44]	<0.001	0.75	1088
Plasma p-tau217 & Tau-PET_{MTL}	288	108	1.40 [1.21, 1.62]	<0.001	1.42 [1.27, 1.58]	<0.001	0.79	1055
Plasma p-tau217 & Tau-PET_{NEO}	288	108	1.40 [1.21, 1.62]	<0.001	1.25 [1.15, 1.35]	<0.001	0.77	1070

Presented effect sizes are derived from from Cox proportional hazard models.

Supplementary Table 13. Comparison (p-values) of different models predicting clinical progression to MCI in Aβ+ participants

**P-values**	Basic without *APOE*	Basic with *APOE*	Plasma p-tau217	Tau-PET$_{MTL}$	Tau-PET$_{NEO}$	Plasma p-tau217 & Tau-PET$_{MTL}$	Plasma p-tau217 & Tau-PET$_{NEO}$
**Aβ+ participants**							
Basic without APOE	1	0.524	0,010	<0.001	0.002	<0.001	0.001
Basic with APOE		1	0.004	<0.001	0.003	<0.001	0.001
Plasma p-tau217			1	0.200	0.711	0.007	0.031
Tau-PET$_{MTL}$				1	0.209	0.042	0.891
Tau-PET$_{NEO}$					1	0.024	0.061
Plasma p-tau217 & Tau-PET$_{MTL}$						1	0.133
Plasma p-tau217 & Tau-PET$_{NEO}$							1

Numbers represent p-values derived from Cox proportional hazard models comparing different models (rows vs columns).

Supplementary Figure 5. Two-step approach for clinical trials using clinical progression using Tau-PET$_{\text{NEO}}$

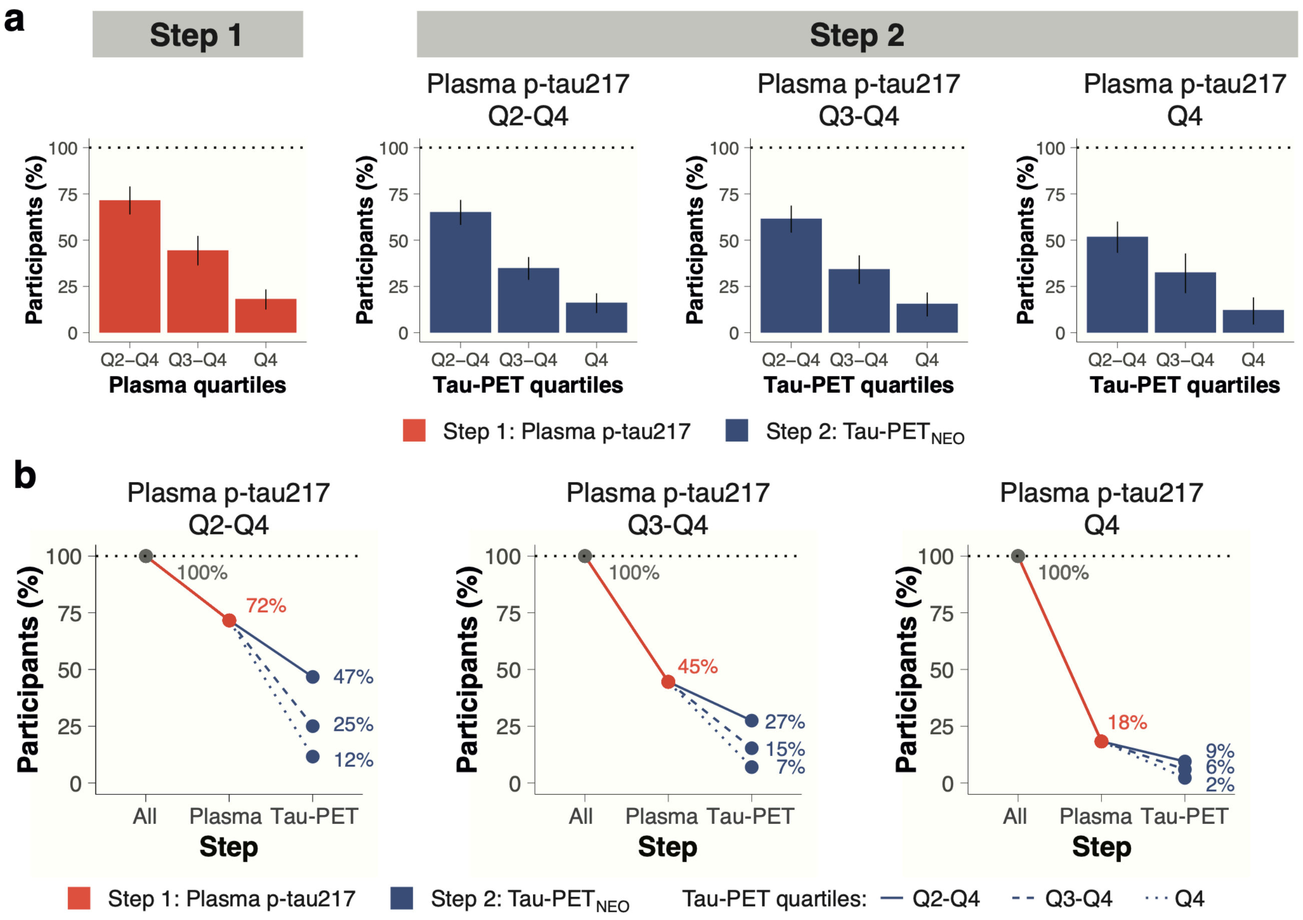

a, the obtained sample size reduction using different percentiles (75th, 50th and 25th) of the samples' baseline plasma p-tau217 baseline levels using the mPACC5 as the primary endpoint (step 1). Then, we repeated the approach selecting the 75th, 50th and 25th percentiles of the new samples' Tau-PET$_{\text{NEO}}$ measures (step 2). Red lines represent step 1 with plasma p-tau217 and blue lines represent step 2 with Tau-PET$_{\text{NEO}}$. Different linestyles represent different quartiles of Tau-PET$_{\text{NEO}}$ from those subjects already selected from step 1. Dotted black lines represent 100% participants needed without that step. Note that 100% in step 2 refers to the participants selected by plasma p-tau217 in step 1. **b** shows the calculated sample size reductions for various plasma p-tau217 and Tau-PET$_{\text{NEO}}$ quantile combinations. The analyses presented in this figure are based on 1376 CU individuals.

Supplementary Figure 6. A three-step screening with MCI progression as outcome measure

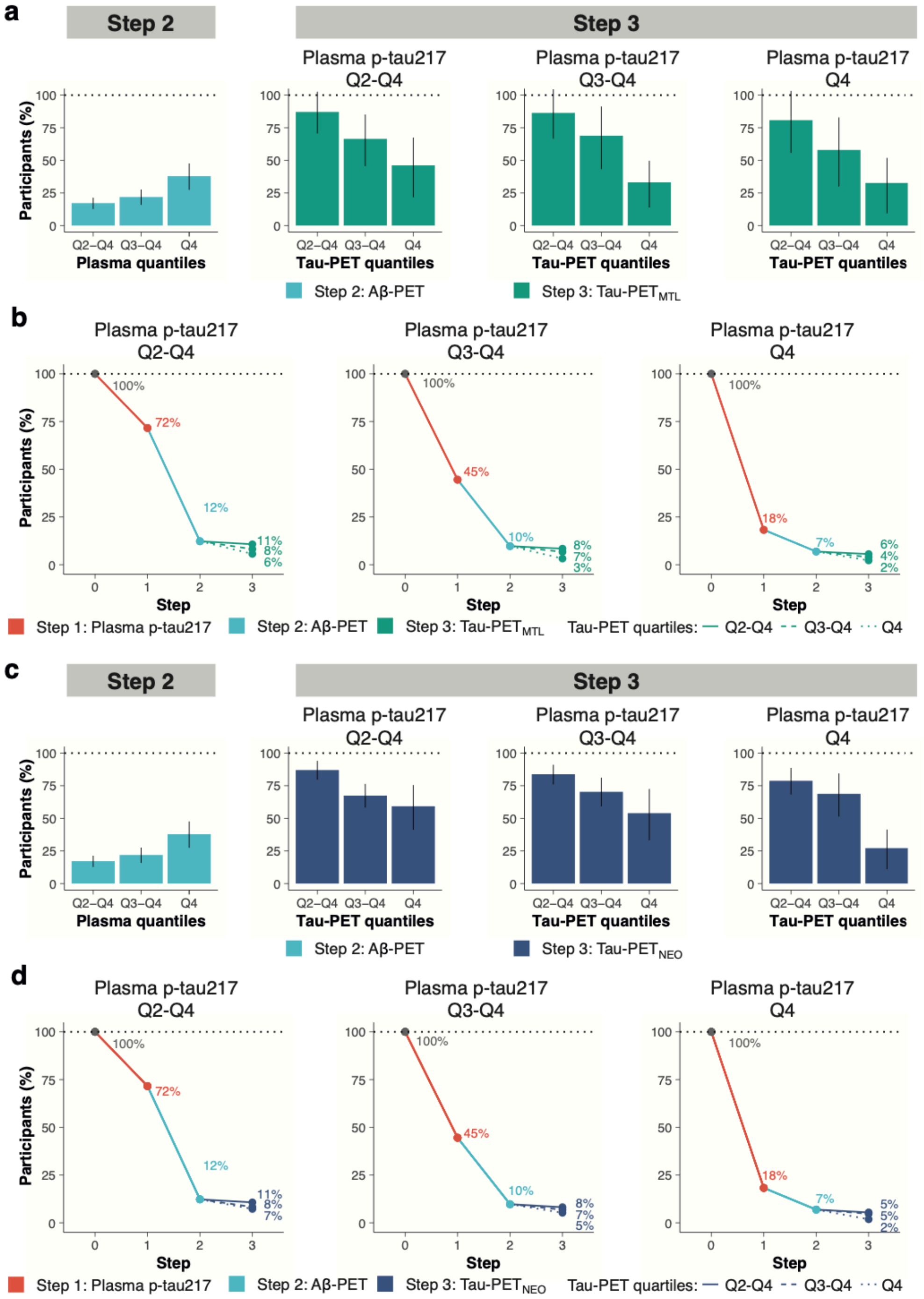

a,c, the obtained sample size reduction using different percentiles (75th, 50th and 25th) of the samples' baseline plasma p-tau217 baseline levels using the mPACC5 as the primary endpoint (step 1). Then, we repeated the Aβ-PET positive individuals (step 2) from those selected in step 1. Finally, we repeated the approach selecting the 75th, 50th and 25th percentiles of the new samples' Tau-PET$_{MTL}$ (**a**) Tau-PET$_{NEO}$ (**c**) measures (step 3) from those already selected in step 2. Dotted black lines represent 100% participants needed without that step. Note that 100% in step 2 refers to the participants selected by plasma p-tau217. Also, 100% in step 3 in step 1 refers to the participants selected by plasma p-tau217 and Aβ-PET positivity (step 2). **b** shows the calculated sample size reductions for various plasma p-tau217 and Tau-PET$_{NEO}$ quantile combinations. Red lines represent step 1 with plasma p-tau217, light blue lines represent Aβ-PET, and green/dark blue lines represent step 2 with Tau-PET$_{MTL}$/Tau-PET$_{NEO}$. Different linestyles represent different quartiles of Tau-PET$_{NEO}$ from those subjects already selected from step 1. The analyses presented in this figure are based on 1426 CU individuals.

Supplementary Figure 7. Characterization of different plasma p-tau217/Tau-PET$_{NEO}$ groups

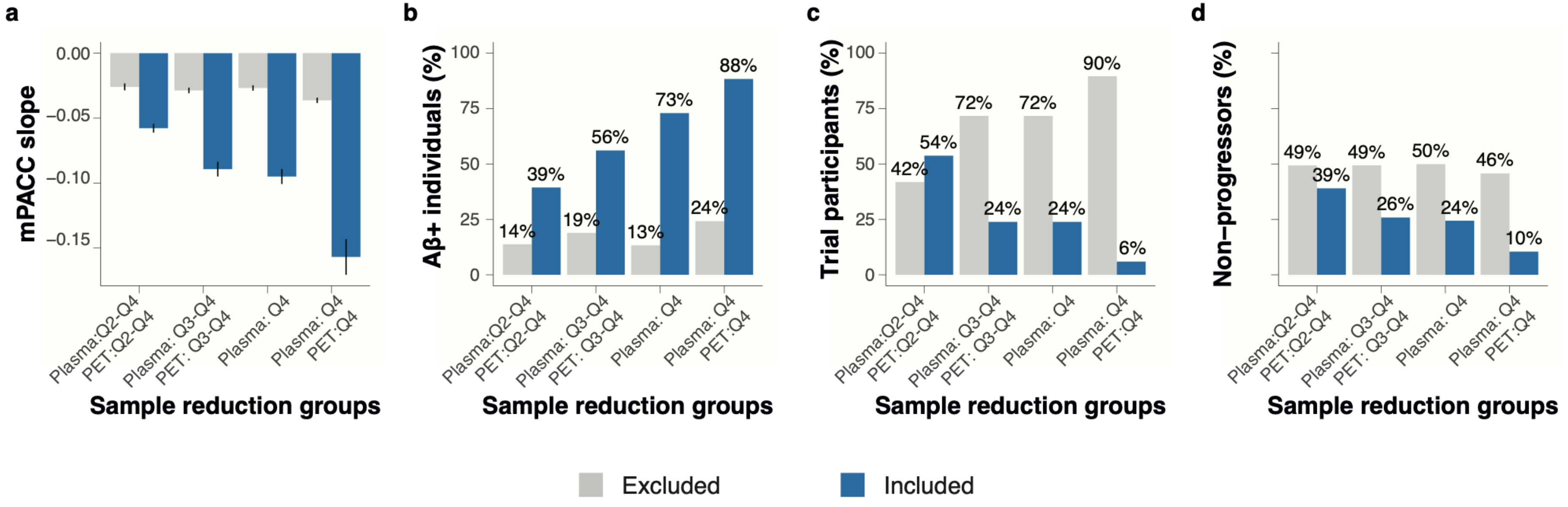

This figure shows how different group compositions based on their baseline plasma p-tau217 and Tau-PET$_{NEO}$ levels are related to various relevant trial metrics, including the annual mPACC5 slope (**a**, n=1376), proportion of Aβ+ individuals (**b**, n=1473), the proportion of individuals from the entire population that would be included in a clinical trial based on the group definitions described on the x-axis (**c**, all participants) and the proportion of "non-progressors" on the mPACC5 (defined as slope > -0.016, see Methods section for details) (**d**, n=1376). Error bars in **a** represent the 95% CI around the mean. More efficient trials are expected with lower mPACC slopes, higher percentages of Aβ+ individuals and trial participants, but lower percentages of non-progressors

Supplementary Table 14. Sample size reductions in a clinical trial following a two-step approach

Step 1. Quantile Plasma	Step 2. Quantile PET	Plasma (%)	Tau-PET$_{MTL}$ (%)	Tau-PET$_{NEO}$ (%)	Tau-PET$_{MTL}$ (%, ref plasma)	Tau-PET$_{NEO}$ (%, ref plasma)
**Modified Preclinical Alzheimer Cognitive Composite 5 (mPACC5)**						
**Q2-Q4**	Q2-Q4	68[59, 86]	51[44, 72]	56[49, 78]	75[65, 96]	83[73, 105]
	Q3-Q4		35[32, 53]	43[37, 64]	52[46, 74]	63[54, 88]
	Q4		18[15, 28]	22[17, 34]	27[21, 40]	32[23, 48]
**Q3-Q4**	Q2-Q4	36[28, 49]	28[22, 41]	29[23, 42]	79[65, 97]	81[70, 97]
	Q3-Q4		19[15, 29]	22[18, 33]	54[43, 71]	61[51, 82]
	Q4		12[10, 19]	15[11, 24]	34[27, 49]	43[30, 62]
**Q4**	Q2-Q4	18[14, 27]	15[12, 24]	17[13, 26]	84[68, 108]	94[79, 114]
	Q3-Q4		10[8, 16]	13[10, 20]	56[42, 77]	69[52, 94]
	Q4		7[6, 12]	9[7, 14]	39[29, 59]	48[31, 73]
**Clinical progression to mild cognitive impairment (MCI)**						
**Q2-Q4**	Q2-Q4	72[64, 79]	60[49, 70]	47[40, 53]	84[71, 96]	65[58, 72]
	Q3-Q4		44[31, 55]	25[20, 30]	61[45, 75]	35[29, 41]
	Q4		25[13, 34]	12[7, 15]	34[19, 48]	16[11, 21]
**Q3-Q4**	Q2-Q4	45[36, 52]	37[27, 46]	27[21, 33]	83[68, 97]	62[54, 69]
	Q3-Q4		29[18, 39]	15[11, 19]	66[46, 84]	34[26, 42]
	Q4		12[5, 17]	7[4, 10]	26[13, 37]	16[9, 22]
**Q4**	Q2-Q4	18[13, 23]	16[9, 22]	9[6, 13]	89[68, 109]	52[43, 60]
	Q3-Q4		12[6, 18]	6[3, 8]	66[39, 91]	33[21, 43]
	Q4		4[1, 7]	2[1, 4]	24[7, 38]	12[4, 19]

Table shows the percentages of participants required for a clinical based on the different plasma (step 1) and Tau-PET (step 2) combinations. Results are shown for Tau-PET$_{MTL}$ and Tau-PET$_{MTL}$, both in actual percentages and in percentages relative to the reductions already achieved by plasma p-tau217 (columns including label "(%, ref plasma)". Data are presented for both mPACC5 (top) and progression to MCI (bottom) and are based on the assumption of 80% power to detect a 30% change during a 4-year clinical trial.

Supplementary Table 15. Combined plasma p-tau217 and Tau-PET$_{MTL}$ group characterizations: Aβ status and clinical outcomes

				INCLUDED POPULATION			EXCLUDED POPULATION		
Plasma	PET	Excluded	Included	Aβ+	mPACC slope	% Progressors	Aβ+	mPACC slope	% Progressors
Q2-Q4	All	344	1032	35.0%	-0.05 (0.09)	59.1%	7.8%	-0.02 (0.06)	48.5%
Q2-Q4	Q2-Q4	602	774	40.8%	-0.06 (0.09)	62.4%	12.0%	-0.02 (0.06)	48.8%
Q2-Q4	Q3-Q4	860	516	48.4%	-0.08 (0.10)	68.4%	16.0%	-0.02 (0.07)	49.3%
Q2-Q4	Q4	1118	258	67.8%	-0.11 (0.11)	79.8%	19.1%	-0.03 (0.07)	51.1%
Q3-Q4	All	688	688	47.4%	-0.07 (0.10)	66.3%	9.0%	-0.02 (0.06)	46.7%
Q3-Q4	Q2-Q4	860	516	53.7%	-0.08 (0.10)	70.5%	12.9%	-0.02 (0.06)	48.0%
Q3-Q4	Q3-Q4	1032	344	62.2%	-0.10 (0.11)	76.2%	16.9%	-0.03 (0.07)	49.9%
Q3-Q4	Q4	1204	172	82.0%	-0.13 (0.12)	85.5%	20.5%	-0.03 (0.07)	52.3%
Q4	All	1032	344	73.0%	-0.09 (0.11)	75.6%	13.3%	-0.03 (0.07)	50.1%
Q4	Q2-Q4	1118	258	79.8%	-0.11 (0.11)	79.1%	16.3%	-0.03 (0.07)	51.3%
Q4	Q3-Q4	1204	172	88.4%	-0.13 (0.12)	85.5%	19.6%	-0.03 (0.07)	52.3%
Q4	Q4	1290	86	96.5%	-0.17 (0.13)	89.5%	23.6%	-0.04 (0.07)	54.3%

mPACC slopes are expressed as standardized beta-coefficients with a 95% confidence around the mean.

Supplementary Table 16. Combined plasma p-tau217 and Tau-PET$_{MTL}$ group characterizations: Demographic information

			INCLUDED POPULATION					EXCLUDED POPULATION			
Plasma	PET		Age	Females	Education	*APOE* ε4+		Age	% female	Education	*APOE* ε4+
Q2-Q4	All		70.0 (10.3)	51.6%	13.9 (3.3)	40.2%		66.7 (10.5)	56.1%	14.1 (3.5)	27.0%
Q2-Q4	Q2-Q4		71.3 (9.9)	49.6%	14.0 (3.3)	41.5%		66.5 (10.6)	56.8%	13.9 (3.3)	31.1%
Q2-Q4	Q3-Q4		73.1 (9.0)	50.8%	13.9 (3.3)	44.0%		66.8 (10.6)	54.0%	14.1 (3.3)	32.7%
Q2-Q4	Q4		74.5 (7.8)	51.6%	13.7 (3.5)	49.6%		68.0 (10.6)	53.0%	14.1 (3.3)	34.0%
Q3-Q4	All		71.2 (10.3)	50.6%	13.9 (3.4)	45.3%		67.2 (10.3)	54.9%	14.0 (3.2)	28.5%
Q3-Q4	Q2-Q4		72.5 (9.7)	49.6%	13.9 (3.5)	47.5%		67.2 (10.4)	54.7%	14.0 (3.2)	30.6%
Q3-Q4	Q3-Q4		73.8 (9.1)	50.3%	13.7 (3.4)	50.6%		67.7 (10.5)	53.6%	14.1 (3.3)	32.4%
Q3-Q4	Q4		75.3 (7.2)	55.2%	13.6 (3.6)	57.6%		68.3 (10.6)	52.4%	14.0 (3.3)	34.0%
Q4	All		73.6 (9.2)	52.6%	13.8 (3.5)	51.7%		67.7 (10.5)	52.8%	14.0 (3.3)	32.0%
Q4	Q2-Q4		74.8 (8.5)	51.9%	13.8 (3.6)	55.0%		67.9 (10.5)	53.0%	14.0 (3.3)	32.7%
Q4	Q3-Q4		75.3 (7.9)	53.5%	13.6 (3.5)	60.05%		68.3 (10.5)	52.7%	14.0 (3.3)	33.6%
Q4	Q4		74.0 (7.7)	59.3%	13.4 (3.5)	66.3%		68.9 (10.6)	52.3%	14.0 (3.3)	35.0%

Table shows the characteristics of populations included and excluded for a clinical trial based on different plasma (step 1) and Tau-PET (step 2) combinations.

Supplementary Figure 8. Relevant trial outcomes when using predefined cut-offs in BioFINDER-2

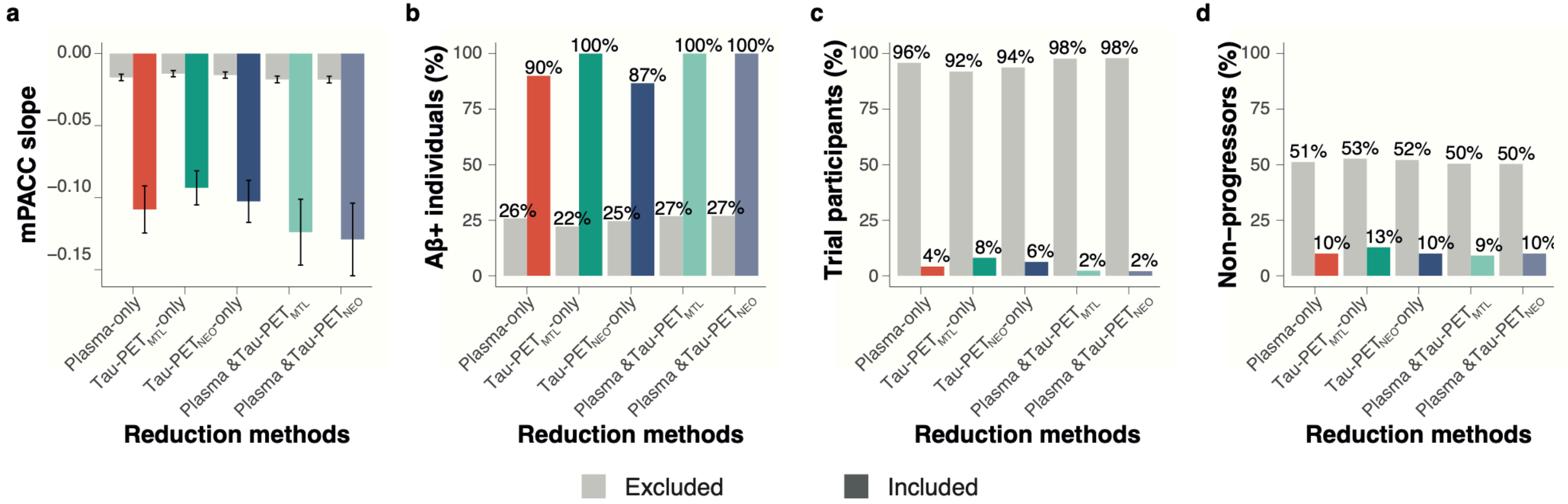

Using pre-specified cut-offs in the BioFINDER-2 cohort, this figure shows how different group compositions based on different methods are related to various relevant trial metrics, including the annual mPACC5 slope (**a**), proportion of Aβ+ individuals (**b**), the proportion of individuals from the entire population that would be included in a clinical trial based on the group definitions described on the x-axis (**c**) and the proportion of “non-progressors” on the mPACC5 (defined as slope > -0.016, see Methods section for details) (**d**). Errorbars in **a** represent the 95% CI. More efficient trials are expected with lower mPACC slopes, higher percentages of Aβ+ individuals and trial participants, but lower percentages of non-progressors. The analyses presented in this figure are based on 441 CU individuals.

Supplementary Figure 9. Projected costs that could be saved in a hypothetical trial with mPACC5 as an endpoint

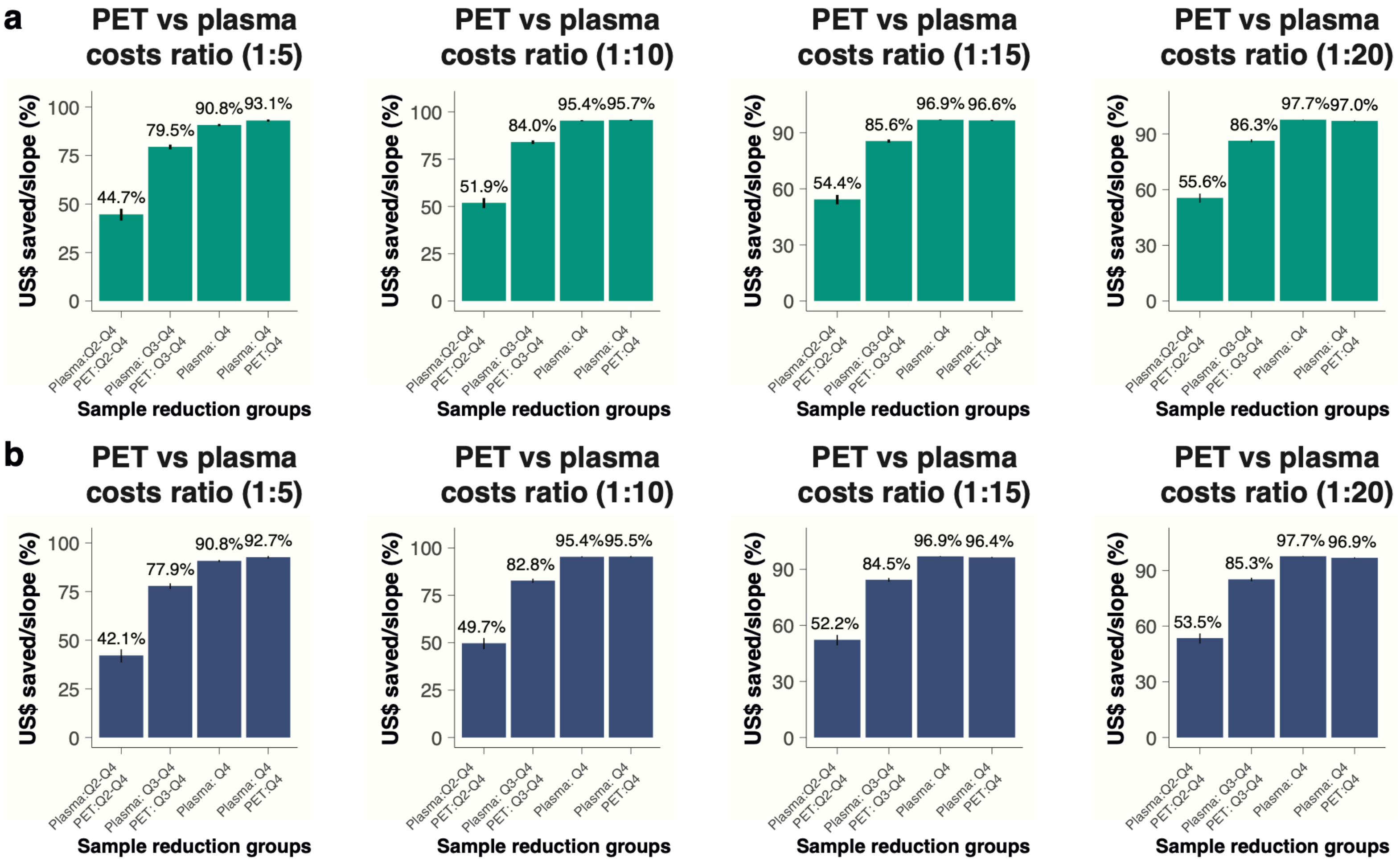

Figure shows the % of cost reductions that can be achieved when implementing different Tau-PET (Tau-PET$_{MTL}$ in panel **a,** Tau-PET$_{NEO}$ in panel **b**) vs plasma p-tau217 combinations when using the mPACC as an endpoint. The ratio of 1:5 reflects that the cost of 1 Tau-PET scan resembles the cost of 5 plasma p-tau217 assessment.

Supplementary Figure 10. Projected costs that could be saved in a hypothetical trial with clinical progression to MCI as an endpoint

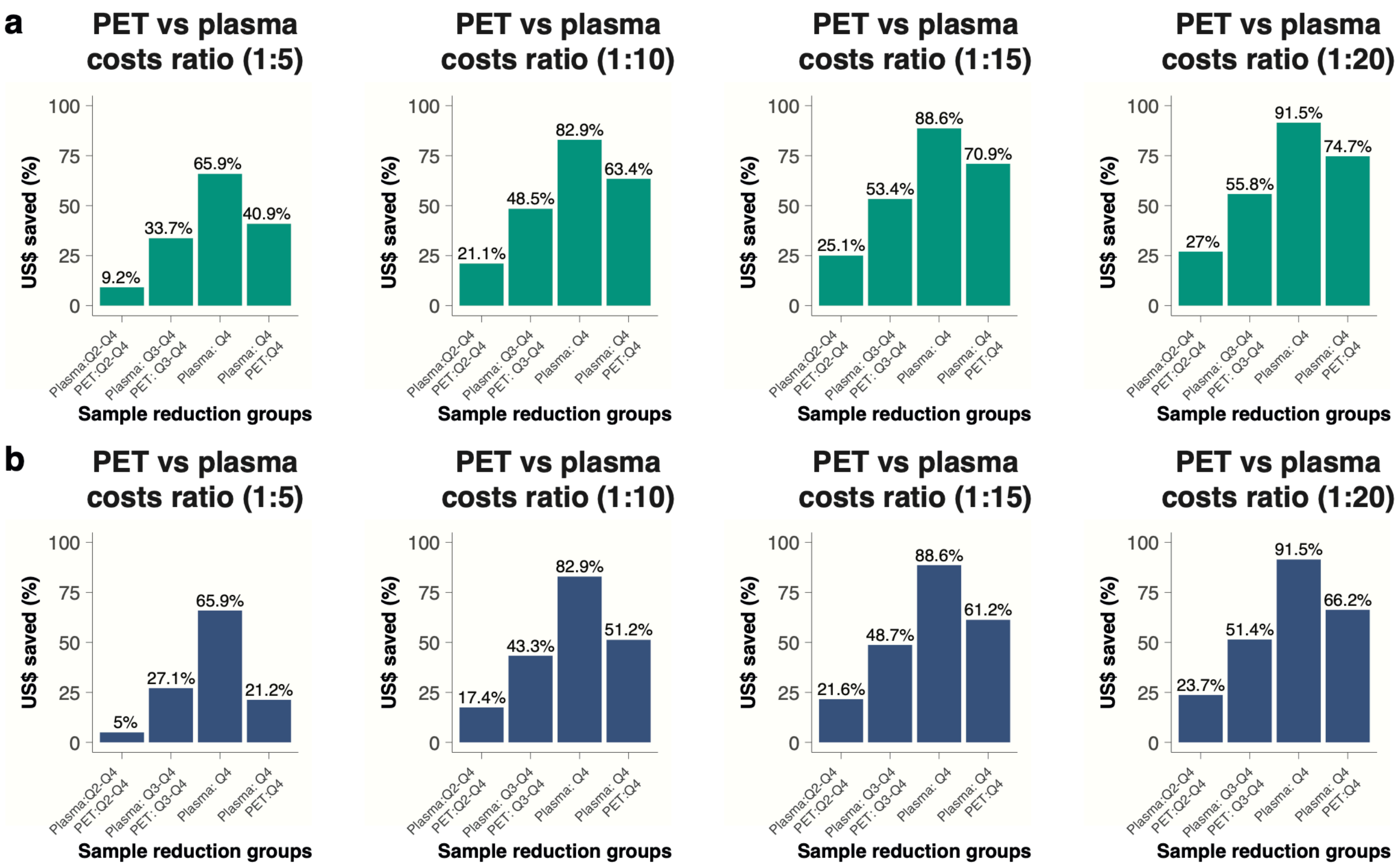

The % of cost reductions that can be achieved when implementing different Tau-PET (Tau-PET$_{MTL}$ in panel **a,** Tau-PET$_{NEO}$ in panel **b**) vs plasma p-tau217 combinations when using clinical progression to MCI as an endpoint. The ratio of 1:5 reflects that the cost of 1 Tau-PET scan resembles the cost of 5 plasma p-tau217 assessment.

Supplementary Table 17. Cohort descriptions

Cohort	Cohort description	References
BioFINDER-1 & BioFINDER-2	The Swedish BioFINDER studies are longitudinal studies covering the entire AD continuum in which participants were recruited at Skåne University Hospital and the Hospital of Angelholm, Sweden. The main inclusion criteria were absence of cognitive symptoms as assessed by a physician with special interest in cognitive disorders, being fluent in Swedish, having no significant unstable systemic illness that made it difficult to participate in the study, having no current significant alcohol or substance misuse, and no significant neurological or psychiatric illness. For the current study participants above > 50 years old were included. Both cognitively healthy older adults and SCD participants were included. The SCD participants were referred from participating memory clinic because of cognitive complaints, but did not fulfill criteria for MCI (defined using criteria by Petersen and operationalized according to[1,2]) following a neuropsychological test battery.	[3,4]
MCSA	The Mayo Clinic Study of Aging (MCSA) is a longitudinal population-based study of cognitive aging in Olmsted County, Minnesota. The study was designed to study prevalence, incidence and risk factors for MCI and dementia. Potential participants are randlomly enumerated from the Olmsted County, MN, census and enrolled by age/sex strata. Enumeration is repeated to maintain a sample of approximately 3000 active participants. At entry, every person underwent evaluations that included a medical history review and interview with the participant and a study partner, a neurological examination by a physician; and a neuropsychological examination. For this study, participants were considered MCI only if the study coordinator, physician, and neuropsychologist were all in agreement regarding the MCI diagnosis. Participants were judged cognitively normal if they did not meet MCI criteria. Participants aged between 50 and 89 years old were included in the current study.	[5]
Knight ADRC	The Charles F. and Joanne Knight Alzheimer Disease Research Center (Knight ADRC) is one of approximately 30 Centers funded by the National Institute on Aging (NIA) located at major medical institutions across the United States. Researchers at these Centers are working to translate research advances into improved diagnosis and care for people with Alzheimer disease, as well as working to find a treatment or way to prevent Alzheimer disease and other types of dementia.	[6]
PREVENT-AD	The PREVENT-AD (Pre-symptomatic Evaluation of Experimental or Novel Treatments for Alzheimer Disease) cohort is composed of cognitively healthy participants over 55 years old, at risk of developing Alzheimer Disease (AD) as their parents and/or siblings were/are affected by the disease. These	[7]

	'at-risk' participants have been followed for a naturalistic study of the presymptomatic phase of AD since 2011 using multimodal measurements of various disease indicators. Two clinical trials intended to test pharmaco-preventive agents have also been conducted.	
AIBL	The Australian Imaging, Biomarker & Lifestyle Flagship Study of Ageing (AIBL) is a longitudinal, prospective cohort with participants coming from two-site study – Melbourne and Perth. To be included in the study, participants were (1) ≥60 years old; (2) fluent in English; (4) had completed at least 7 years of education; (5) did not have any history of neurological or psychiatric disorders, drug or alcohol abuse or dependence, or any other unstable medical condition; and (6) were deemed to be cognitively unimpaired (CU), based on their performance on a battery of cognitive assessments that AIBL participants undergo every 12 to 18 months. A multidisciplinary clinical review panel determines whether an individual is CU, based on the available clinical and neuropsychological information.	8
ADC	The Amsterdam Dementia Cohort (ADC) is a prospective cohort study including (amongst others) individuals with subjective cognitive decline (SCD) presenting at the Alzheimer Center of the VU University Medical Center Amsterdam. All participants have been referred to the memory clinic by their general practitioner, and a neurologist or geriatrician in the case of a second opinion for evaluation of cognitive complaints. They receive standardized dementia screening at the memory clinic, including an interview with a neurologist, physical and neurological examination, neuropsychological assessment. Individuals with SCD can additionally be included in the SCIENCe study, for which the main inclusion criteria are a diagnosis of SCD (i.e., cognitive complaints and normal cognition) and age ≥ 45 years. Exclusion criteria for participation in the SCIENCe study are MCI, dementia, major psychiatric disorder (i.e., current depression, personality disorders, schizophrenia), neurological diseases known to cause memory complaints (i.e., Parkinson's disease, epilepsy), HIV, abuse of alcohol or other substances, and language barrier.	9
WRAP	The Wisconsin Registry for Alzheimer's Prevention is a longitudinal observational cohort study enriched with persons with a parental history (PH) of probable Alzheimer's disease (AD) dementia. Recruitment sources included memory clinics in which a parent was diagnosed or treated, limited radio and newspaper advertisements, and word of mouth. Participants generally meet the following inclusion criteria at study entry: age 40–65 years; fluent English speaker; visual and auditory acuity adequate for neuropsychological testing; good health with no diseases expected to interfere with study participation over time. Participants are excluded from enrollment if they have a prior diagnosis of dementia or evidence of dementia at baseline testing (one was excluded due to baseline dementia).	10

TRIAD	The Translational Biomarkers of Aging and Dementia (TRIAD) cohort study is a longitudinal observational cohort study in Montréal, Québec, Canada. Participants are recruited from the community and from the the McGill Centre for Studies in Aging. All participants are clinically evaluated by dementia specialists. Participants were excluded from this study if they had systemic conditions which were not adequately controlled through a stable medication regimen. Other exclusion criteria were active substance abuse, recent head trauma, recent major surgery, or MRI/PET safety contraindications. The study was approved by the Montreal Neurological Institute PET working committee and the Douglas Mental Health University Institute Research Ethics Board. Written informed consent was obtained for all participants.	11

Supplementary Table 18. Methods to determine Amyloid PET status by cohort

Cohort	Tracer	Methodology	Cut-off	References
BioFINDER-1	[^{18}F]flutemetamol	Global neocortical composite standardized uptake value ratios (SUVR) for the 90-110min interval p.i. with whole cerebellum as reference region	>1.03 SUVR	[4]
BioFINDER-2	[^{18}F]flutemetamol	Global neocortical composite SUVR for the 90-110min interval p.i. with whole cerebellum as reference region	>1.03 SUVR	[4]
MCSA	[^{11}C]PIB	Late uptake amyloid PET images were acquired from 40-60 minutes p.i. A meta-ROI was calculated as the voxel-number weighted average of uptake in a target region including prefrontal, orbitofrontal, parietal, temporal, anterior and posterior cingulate, and precuneus regions divided by the uptake in the cerebellar crus gray matter.	>1.48 SUVR (>21CL)	[5]
Knight ADRC	[^{11}C]PIB	Data were processed using a region of interest approach using Freesurfer. Amyloid deposition was summarized using the average across the left and right lateral orbitofrontal, medial orbitofrontal, rostral middle frontal, superior frontal, superior temporal, middle temporal, and precuneus regions.	>20 CL	[6]
PREVENT-AD	[^{18}F]NAV4694	Aβ-PET images were realigned onto their respective MRI, masked to remove the scalp and CSF in an attempt to avoid contamination by nongray or nonwhite matter voxels, and smoothed using a full width at half maximum Gaussian kernel of 8mm. Resulting images were scaled using whole cerebellum uptake values (whole cerebellum was preferred to cerebellum gray matter to account better for white matter off-target binding variability between tracers). Global neocortical Aβ burden was quantified by extracting, in native space, the mean standardized uptake value ratio (SUVR) of the frontal, temporal, parietal, and posterior cingulate cortex of the Desikan-Killiany atlas	>1.33 SUVR	[12]
AIBL	[[^{18}F]NAV4694	The standard Centiloid (CL) cortical and whole cerebellar volumes of interest template were applied to the summed and spatially normalised PET images in order to obtain SUVR's. These SUVR were transformed into CL units by linear transformation using the PET tracer-specific equations published for conversion of CL method SUVR to CL units.	>24 CL	[13]

ADC	$[^{18}F]$florbetapir	Visual read following guidelines provided by Avid Radiopharmaceuticals corresponding to >17 CL.	-	[14]
WRAP	$[^{11}C]$PIB	Amyloid burden was assessed as a global average ^{11}C-PiB distribution volume ratio (DVR; Logan graphical analysis, cerebellum gray matter reference region), taken across 8 bilateral cortical ROIs. A+ was ascertained using a global ^{11}C-PiB DVR≥1.16 a threshold previously shown to predict subsequent amyloid accumulation.	>1.16 DVR	[15]
TRIAD	$[^{18}F]$NAV4694	$[^{18}F]$AZD4694 PET images were acquired 40-70 min after bolus injection and reconstructed on a 4-dimensional volume with 3 frames (3 x 600s). Amyloid-β SUVR from a neocortical region of interest (ROI) for each participant was estimated by averaging the SUVR from the precuneus, prefrontal, orbitofrontal, parietal, temporal, and cingulate cortices, with amyloid-β positivity defined as an $[^{18}F]$AZD4694 above 1.55.	>1.55 SUVR	[16]

CL = Centiloid; DVR = Distribution volume ratio; SUVR = Standardized uptake value ratio.

Centiloid (CL) units were presented when available.

Supplementary Table 19. Methods to determine Tau PET status in the medial temporal lobe (MTL) and neocortex (NEO) by cohort

Cohort	Tracer	Scanning interval	Reference region	Reference
BioFINDER-1	[^{18}F]flortaucipir	80-100min p.i.	Inferior cerebellar GM	[17]
BioFINDER-2	[^{18}F]RO948	70-90min p.i.	Inferior cerebellar GM	[18]
MCSA	[^{18}F]flortaucipir	80-100min p.i.	Cerebellar crus GM	[19]
Knight ADRC	[^{18}F]flortaucipir	80-100min p.i.	Cerebellar GM	[6]
PREVENT-AD	[^{18}F]flortaucipir	80-100min p.i.	Inferior cerebellar GM	[7]
AIBL	[^{18}F]MK6204	90-110 min p.i.	Cerebellar GM	[13]
ADC	[^{18}F]flortaucipir	80-100min p.i.	Cerebellar GM	[20]
WRAP	[^{18}F]MK6240	70-90min p.i.	Inferior cerebellar GM	[15]
TRIAD	[^{18}F]MK6240	90-100min p.i.	Cerebellar Crus GM	[21]

GM = Gray matter; MTL = Medial temporal lobe; NEO = Neocortical; p.i. = Post-injection; SUVR = Standardized uptake value ratio.

The cut-offs were generated in each individual cohort, based on the mean + 2*standard deviation across all Aβ-negative participants within each cohort. We computed tau PET status for a medial temporal lobe (MTL; unweighted average of bilateral entorhinal cortex and amygdala) and a neocortical (NEO; weighted average of bilateral middle temporal and inferior temporal gyri) region-of-interest.

Supplementary Table 20. Composition of the mPACC5 for each cohort

Cohort	Global Cognition	Episodic Memory	Time executive function	Semantic memory
BioFINDER-1	MMSE	ADAS-COG delayed word recall	Symbol digit modalities test	Animal fluency
BioFINDER-2	MMSE	ADAS-COG delayed word recall	Symbol digit modalities test	Animal fluency
MCSA	MMSE[a]	AVLT delayed recall	WAIS-R Digit Symbol	Sum of animal, fruits and vegetables fluency
Knight ADRC	MMSE	CVLT – Delayed recall	Symbol digit modalities test	Animal fluency
PREVENT-AD	RBANS total score	RBANS – Delayed recall	RBANS - EE	Animal fluency
AIBL	MMSE	CVLT – Delayed recall	Symbol digit modalities test	Sum of animal and names fluency
ADC	MMSE	RAVLT – Delayed recall	TMT-B	Animal fluency
WRAP	MMSE	AVLT – Delayed recall	WAIS-R Digit Symbol	Animal fluency
TRIAD	MMSE	Logical Memory test - Delayed recall	Letter fluency	Category fluency

Note that the episodic memory test was given double weight and thus accounted for 40% of the mPACC5 score.

[a] A 38-point test, the Short Test of Mental Status (STMS)[22], was converted to MMSE scores using an in-house developed algorithm[23].

REFERENCES

1. Palmqvist, S., *et al.* Discriminative Accuracy of Plasma Phospho-tau217 for Alzheimer Disease vs Other Neurodegenerative Disorders. *JAMA* **324**, 772-781 (2020).
2. Petrazzuoli, F., *et al.* Brief Cognitive Tests Used in Primary Care Cannot Accurately Differentiate Mild Cognitive Impairment from Subjective Cognitive Decline. *J Alzheimers Dis* **75**, 1191-1201 (2020).
3. Leuzy, A., *et al.* Diagnostic Performance of RO948 F 18 Tau Positron Emission Tomography in the Differentiation of Alzheimer Disease From Other Neurodegenerative Disorders. *JAMA Neurol* **77**, 955-965 (2020).
4. Palmqvist, S., *et al.* Accuracy of brain amyloid detection in clinical practice using cerebrospinal fluid beta-amyloid 42: a cross-validation study against amyloid positron emission tomography. *JAMA Neurol* **71**, 1282-1289 (2014).
5. Jack, C.R., Jr. & Holtzman, D.M. Biomarker modeling of Alzheimer's disease. *Neuron* **80**, 1347-1358 (2013).
6. Gordon, B.A., *et al.* The relationship between cerebrospinal fluid markers of Alzheimer pathology and positron emission tomography tau imaging. *Brain* **139**, 2249-2260 (2016).
7. Strikwerda-Brown, C., *et al.* Association of Elevated Amyloid and Tau Positron Emission Tomography Signal With Near-Term Development of Alzheimer Disease Symptoms in Older Adults Without Cognitive Impairment. *JAMA Neurol* **79**, 975-985 (2022).
8. Fowler, C., *et al.* Fifteen Years of the Australian Imaging, Biomarkers and Lifestyle (AIBL) Study: Progress and Observations from 2,359 Older Adults Spanning the Spectrum from Cognitive Normality to Alzheimer's Disease. *J Alzheimers Dis Rep* **5**, 443-468 (2021).
9. Slot, R.E.R., *et al.* Subjective Cognitive Impairment Cohort (SCIENCe): study design and first results. *Alzheimers Res Ther* **10**, 76 (2018).
10. Johnson, S.C., *et al.* The Wisconsin Registry for Alzheimer's Prevention: A review of findings and current directions. *Alzheimers Dement (Amst)* **10**, 130-142 (2018).
11. Pascoal, T.A., *et al.* Discriminative accuracy of the A/T/N scheme to identify cognitive impairment due to Alzheimer's disease. *Alzheimers Dement (Amst)* **15**, e12390 (2023).
12. Gonneaud, J., *et al.* Association of education with Abeta burden in preclinical familial and sporadic Alzheimer disease. *Neurology* **95**, e1554-e1564 (2020).
13. Krishnadas, N., *et al.* Rates of regional tau accumulation in ageing and across the Alzheimer's disease continuum: an AIBL (18)F-MK6240 PET study. *EBioMedicine* **88**, 104450 (2023).
14. Coomans, E.M., *et al.* Performance of a [(18)F]Flortaucipir PET Visual Read Method Across the Alzheimer Disease Continuum and in Dementia With Lewy Bodies. *Neurology* **101**, e1850-e1862 (2023).
15. Cody, K.A., *et al.* Characterizing brain tau and cognitive decline along the amyloid timeline in Alzheimer's disease. *Brain* (2024).
16. Therriault, J., *et al.* Determining Amyloid-beta Positivity Using (18)F-AZD4694 PET Imaging. *J Nucl Med* **62**, 247-252 (2021).
17. Ossenkoppele, R., *et al.* Associations between tau, Abeta, and cortical thickness with cognition in Alzheimer disease. *Neurology* **92**, e601-e612 (2019).
18. Coomans, E.M., *et al.* Interactions between vascular burden and amyloid-beta pathology on trajectories of tau accumulation. *Brain* **147**, 949-960 (2024).

19. Jack, C.R., *et al.* The bivariate distribution of amyloid-beta and tau: relationship with established neurocognitive clinical syndromes. *Brain* **142**, 3230-3242 (2019).
20. Visser, D., *et al.* Tau pathology as determinant of changes in atrophy and cerebral blood flow: a multi-modal longitudinal imaging study. *Eur J Nucl Med Mol Imaging* **50**, 2409-2419 (2023).
21. Pascoal, T.A., *et al.* In vivo quantification of neurofibrillary tangles with [(18)F]MK-6240. *Alzheimers Res Ther* **10**, 74 (2018).
22. Kokmen, E., Smith, G.E., Petersen, R.C., Tangalos, E. & Ivnik, R.C. The short test of mental status. Correlations with standardized psychometric testing. *Arch Neurol* **48**, 725-728 (1991).
23. Tang-Wai, D.F., *et al.* Comparison of the short test of mental status and the mini-mental state examination in mild cognitive impairment. *Arch Neurol* **60**, 1777-1781 (2003).

Acknowledgements for the contributors to the PREVENT-AD Dataset

Full Name	Citation Name	Affiliations	Degrees	Titles	Roles
Sylvia Villeneuve	Villeneuve, Sylvia	• McGill University, Montreal, QC, CA<br>◦ Douglas Mental Health University Institute Research Centre, Montreal, QC, CA<br>▪ StoP-Alzheimer Centre, Montreal, QC, CA	• PhD		• Director<br>• Investigator<br>• Processing and Evaluation (MRI/PET/MEG)<br>• Project Administration
Judes Poirier	Poirier, Judes	• McGill University, Montreal, QC, CA<br>◦ Douglas Mental Health University Institute Research Centre, Montreal, QC, CA<br>▪ StoP-Alzheimer Centre, Montreal, QC, CA	• PhD		• Co-Director<br>• Investigator<br>• Genetic Analysis and Biochemical Assays
John C.S. Breitner	Breitner, John	• McGill University, Montreal, QC, CA<br>◦ Douglas Mental Health University Institute Research Centre, Montreal, QC, CA<br>▪ StoP-Alzheimer Centre, Montreal, QC, CA	• MD<br>• MPH		• Director Emeritus<br>• Investigator<br>• Project Administration
Mohamed Badawy	Badawy, Mohamed	• McGill University, Montreal, QC, CA<br>◦ Montreal Neurological Institute and Hospital, Montreal, QC, CA	• MD		• Investigator<br>• LP/CSF Collection
Sylvain Baillet	Baillet, Sylvain	• McGill University, Montreal, QC, CA<br>◦ Montreal Neurological Institute and Hospital, Montreal, QC, CA	• PhD		• Investigator
Andrée-Ann Baril	Baril, Andrée-Ann	• McGill University, Montreal, QC, CA<br>◦ Douglas Mental Health University Institute Research Centre, Montreal, QC, CA<br>▪ StoP-Alzheimer Centre, Montreal, QC, CA	• PhD		• Investigator<br>• Data Analysis

Pierre Bellec	Bellec, Pierre	• Université de Montréal, Montreal, QC, CA<br>◦ Centre de recherche Institut Universitaire de Gériatrie de Montréal, Montreal, QC, CA<br>• McGill University, Montreal, QC, CA<br>◦ Douglas Mental Health University Institute Research Centre, Montreal, QC, CA<br>▪ StoP-Alzheimer Centre, Montreal, QC, CA	• PhD		• Investigator<br>• Data Analysis<br>• Processing and Evaluation (MRI/PET/MEG)
Véronique Bohbot	Bohbot, Véronique	• McGill University, Montreal, QC, CA<br>◦ Douglas Mental Health University Institute Research Centre, Montreal, QC, CA<br>▪ StoP-Alzheimer Centre, Montreal, QC, CA	• PhD		• Investigator
Danilo Bzdok	Bzdok, Danilo	• McGill University, Montreal, QC, CA	• PhD		• Investigator
Mallar Chakravarty	Chakravarty, Mallar	• McGill University, Montreal, QC, CA<br>◦ Douglas Mental Health University Institute Research Centre, Montreal, QC, CA<br>▪ StoP-Alzheimer Centre, Montreal, QC, CA	• PhD		• Investigator<br>• Processing and Evaluation (MRI/PET/MEG)
D. Louis Collins	Collins, D. Louis	• McGill University, Montreal, QC, CA<br>◦ Montreal Neurological Institute and Hospital, Montreal, QC, CA<br>◦ Douglas Mental Health University Institute Research Centre, Montreal, QC, CA<br>▪ StoP-Alzheimer Centre, Montreal, QC, CA	• PhD		• Investigator<br>• Data Analysis<br>• Processing and Evaluation (MRI/PET/MEG)
Mahsa Dadar	Dadar, Mahsa	• McGill University, Montreal, QC, CA<br>◦ Montreal Neurological Institute and Hospital, Montreal, QC, CA	• PhD		• Investigator<br>• Data Analysis<br>• Processing and Evaluation (MRI/PET/MEG)

Simon Ducharme	Ducharme, Simon	• McGill University, Montreal, QC, CA<br>◦ Montreal Neurological Institute and Hospital, Montreal, QC, CA<br>◦ Douglas Mental Health University Institute Research Centre, Montreal, QC, CA<br>▪ StoP-Alzheimer Centre, Montreal, QC, CA	• MD		• Investigator<br>• Clinical Evaluation<br>• LP/CSF Collection
Alan Evans	Evans, Alan	• McGill University, Montreal, QC, CA<br>◦ Montreal Neurological Institute and Hospital, Montreal, QC, CA<br>◦ Douglas Mental Health University Institute Research Centre, Montreal, QC, CA<br>▪ StoP-Alzheimer Centre, Montreal, QC, CA	• PhD		• Investigator<br>• Processing and Evaluation (MRI/PET/MEG)
Claudine Gauthier	Gauthier, Claudine	• Concordia University, Montreal, QC, CA	• PhD		• Investigator<br>• Data Analysis<br>• Processing and Evaluation (MRI/PET/MEG)
Maiya R. Geddes	Geddes, Maiya	• McGill University, Montreal, QC, CA<br>◦ Montreal Neurological Institute and Hospital, Montreal, QC, CA<br>◦ Douglas Mental Health University Institute Research Centre, Montreal, QC, CA<br>▪ StoP-Alzheimer Centre, Montreal, QC, CA<br>▪ McGill University Research Centre for Studies in Aging, Montreal, QC, CA	• MD		• Investigator<br>• Clinical Evaluation
Rick Hoge	Hoge, Rick	• McGill University, Montreal, QC, CA<br>◦ Montreal Neurological Institute and Hospital, Montreal, QC, CA<br>◦ Douglas Mental Health University Institute Research Centre, Montreal, QC, CA<br>▪ StoP-Alzheimer Centre, Montreal, QC, CA	• PhD		• Investigator<br>• Processing and Evaluation (MRI/PET/MEG)

Yasser Ituria-Medina	Ituria-Medina, Yasser	• McGill University, Montreal, QC, CA<br>◦ Montreal Neurological Institute and Hospital, Montreal, QC, CA<br>◦ Douglas Mental Health University Institute Research Centre, Montreal, QC, CA<br>▪ StoP-Alzheimer Centre, Montreal, QC, CA	• PhD		• Investigator<br>• Data Analysis<br>• Processing and Evaluation (MRI/PET/MEG)
Maxime Montembeault	Montembeault, Maxime	• McGill University, Montreal, QC, CA<br>◦ Douglas Mental Health University Institute Research Centre, Montreal, QC, CA<br>▪ StoP-Alzheimer Centre, Montreal, QC, CA	• PhD		• Investigator<br>• Consultant
Gerhard Multhaup	Multhaup, Gerhard	• McGill University, Montreal, QC, CA	• PhD		• Investigator
Lisa-Marie Münter	Münter, Lisa-Marie	• McGill University, Montreal, QC, CA	• PhD		• Investigator
Natasha Rajah	Rajah, Natasha	• McGill University, Montreal, QC, CA<br>◦ Douglas Mental Health University Institute Research Centre, Montreal, QC, CA<br>▪ StoP-Alzheimer Centre, Montreal, QC, CA	• PhD		• Investigator<br>• Processing and Evaluation (MRI/PET/MEG)
Pedro Rosa-Neto	Rosa-Neto, Pedro	• McGill University, Montreal, QC, CA<br>◦ Douglas Mental Health University Institute Research Centre, Montreal, QC, CA<br>▪ McGill University Research Centre for Studies in Aging, Montreal, QC, CA<br>▪ StoP-Alzheimer Centre, Montreal, QC, CA	• PhD<br>• MD		• Investigator<br>• Clinical Evaluation<br>• LP/CSF Collection
Taylor Schmitz	Schmitz, Taylor	• Western University, London, ON, CA	• PhD		• Investigator
Jean-Paul Soucy	Soucy, Jean-Paul	• McGill University, Montreal, QC, CA<br>◦ Montreal Neurological Institute and Hospital, Montreal, QC, CA<br>◦ Douglas Mental Health University Institute Research Centre, Montreal, QC, CA<br>▪ StoP-Alzheimer Centre, Montreal, QC, CA	• MD		• Investigator

Nathan Spreng	Spreng, Nathan	• McGill University, Montreal, QC, CA<br>◦ Montreal Neurological Institute and Hospital, Montreal, QC, CA	• PhD		• Investigator<br>• Processing and Evaluation (MRI/PET/MEG)
Christine Tardif	Tardif, Christine	• McGill University, Montreal, QC, CA<br>◦ Douglas Mental Health University Institute Research Centre, Montreal, QC, CA<br>▪ StoP-Alzheimer Centre, Montreal, QC, CA	• PhD		• Investigator<br>• Acquisition (MRI/PET/MEG)<br>• Data Analysis<br>• Processing and Evaluation (MRI/PET/MEG)
Etienne Vachon-Presseau	Vachon-Presseau, Etienne	• McGill University, Montreal, QC, CA<br>◦ Douglas Mental Health University Institute Research Centre, Montreal, QC, CA<br>• Northwestern University, Chicago, IL, USA	• PhD		• Investigator<br>• Consultant
Mohammadali Javanray	Javanray, Mohammadali	• McGill University, Montreal, QC, CA	• MSc		• Data Analysis<br>• Processing and Evaluation (MRI/PET/MEG)
Meishan Ai	Ai, Meishan	• Northeastern University, Boston, MA, USA	• BA		• Data Analysis
Philippe Amouyel	Amouyel, Philippe	• Université de Lille, Lille, HDF, FR	• PhD<br>• MD		• Processing and Evaluation (MRI/PET/MEG)
Jiarui Ao	Ao, Jiarui	• McGill University, Montreal, QC, CA	• BSc		• Data Analysis<br>• Genetic Analysis and Biochemical Assays

Gabriel Aumont-Rodrigue	Aumont-Rodrigue, Gabriel	• McGill University, Montreal, QC, CA<br>◦ Douglas Mental Health University Institute Research Centre, Montreal, QC, CA	• MSc		• Data Analysis<br>• Genetic Analysis and Biochemical Assays
Julie Bailly	Bailly, Julie	• McGill University, Montreal, QC, CA<br>◦ Douglas Mental Health University Institute Research Centre, Montreal, QC, CA	• PhD		• Acquisition (MRI/PET/MEG)<br>• Data Entry<br>• Project Administration
Guilia Baracchini	Baracchini, Guilia	• McGill University, Montreal, QC, CA	• PhD		• Data Analysis<br>• Data Entry
Charles Beauchesne	Beauchesne, Charles	• McGill University, Montreal, QC, CA	• BSc		• Acquisition (MRI/PET/MEG)<br>• Data Entry
Kaj Blennow	Blennow, Kaj	• University of Gothenburg, Gothenburg, SE-O, SE	• PhD<br>• MD		• Genetic Analysis and Biochemical Assays
Christian Bocti	Bocti, Christian	• Université de Sherbrooke, Sherbrooke, QC, Canada	• PhD<br>• MD		• Clinical Evaluation<br>• Consultant
Lianne Boisvert	Boisvert, Lianne		• BA		• Data Entry
Daniel Bowie	Bowie, Daniel	• McGill University, Montreal, QC, CA	• PhD		• Data Analysis
Ann Brinkmalm Westman	Brinkmalm Westman, Ann	• University of Gothenburg, Gothenburg, SE-O, SE	• PhD		• Genetic Analysis and Biochemical Assays

Nolan-Patrick Cunningham	Cunningham, Nolan-Patrick	• McGill University, Montreal, QC, CA<br>◦ Douglas Mental Health University Institute Research Centre, Montreal, QC, CA<br>▪ StoP-Alzheimer Centre, Montreal, QC, CA	• BA		• Acquisition (MRI/PET/MEG)<br>• Cognitive Evaluation<br>• Data Entry<br>• Interview Data Collection
Alain Dagher	Dagher, Alain	• McGill University, Montreal, QC, CA<br>◦ Montreal Neurological Institute and Hospital, Montreal, QC, CA	• PhD		• Data Analysis
Xing Dai	Dai, Xing	• McGill University, Montreal, QC, CA<br>◦ Douglas Mental Health University Institute Research Centre, Montreal, QC, CA			• Clinical Evaluation
Thien Thanh Dang-Vu	Dang-Vu, Thien Thanh	• Concordia University, Montreal, QC, CA	• PhD<br>• MD		• Consultant
Samir Das	Das, Samir	• McGill University, Montreal, QC, CA<br>◦ Montreal Neurological Institute and Hospital, Montreal, QC, CA	• MSc		• Database Management
Marina Dauar-Tedeschi	Dauar-Tedeschi, Marina	• McGill University, Montreal, QC, CA<br>◦ Douglas Mental Health University Institute Research Centre, Montreal, QC, CA<br>▪ McGill University Research Centre for Studies in Aging, Montreal, QC, CA<br>▪ StoP-Alzheimer Centre, Montreal, QC, CA	• PhD<br>• MD		• Clinical Evaluation<br>• Data Analysis<br>• Genetic Analysis and Biochemical Assays<br>• LP/CSF Collection

Christine Dery	Dery, Christine	• McGill University, Montreal, QC, CA<br>◦ Douglas Mental Health University Institute Research Centre, Montreal, QC, CA<br>▪ StoP-Alzheimer Centre, Montreal, QC, CA	• MSc		• Cognitive Evaluation<br>• Data Entry<br>• Project Administration
Maxime Descoteaux	Descoteaux, Maxime	• Université de Sherbrooke, Sherbrooke, QC, Canada	• PhD		• Consultant<br>• Processing and Evaluation (MRI/PET/MEG)
Manon Edde	Edde, Manon	• Université de Sherbrooke, Sherbrooke, QC, Canada	• PhD		• Processing and Evaluation (MRI/PET/MEG)
Alfonso Fajardo Valdez	Fajardo Valdez, Alfonso	• McGill University, Montreal, QC, CA	• MSc		• Data Analysis
Sofia Fernandez Lozano	Fernandez Lozano, Sofia	• McGill University, Montreal, QC, CA	• MSc		• Processing and Evaluation (MRI/PET/MEG)
Vladimir Fonov	Fonov, Vladimir	• McGill University, Montreal, QC, CA<br>◦ Montreal Neurological Institute and Hospital, Montreal, QC, CA	• PhD		• Data Analysis<br>• Processing and Evaluation (MRI/PET/MEG)
David G. Morgan	G. Morgan, David	• Michigan State University, Grand Rapids, MI, CA	• MD		• Genetic Analysis and Biochemical Assays
Jonathan Gallago	Gallego, Jonathan	• McGill University, Montreal, QC, CA	• MSc		• Acquisition (MRI/PET/MEG)<br>• Data Analysis

Aurelie Garrone	Garrone, Aurelie	• McGill University, Montreal, QC, CA<br>◦ Douglas Mental Health University Institute Research Centre, Montreal, QC, CA<br>▪ StoP-Alzheimer Centre, Montreal, QC, CA	• BSc		• Acquisition (MRI/PET/MEG)<br>• Cognitive Evaluation<br>• Data Entry<br>• Interview Data Collection
Louise Hudon	Hudon, Louise	• McGill University, Montreal, QC, CA<br>◦ Douglas Mental Health University Institute Research Centre, Montreal, QC, CA<br>▪ StoP-Alzheimer Centre, Montreal, QC, CA	• BSc		• Cognitive Evaluation<br>• Interview Data Collection
Adam Hull	Hull, Adam	• McGill University, Montreal, QC, CA	• PhD		• Data Entry
Gabriel Jean	Jean, Gabriel	• McGill University, Montreal, QC, CA<br>◦ Douglas Mental Health University Institute Research Centre, Montreal, QC, CA<br>▪ StoP-Alzheimer Centre, Montreal, QC, CA	• MSc		• Acquisition (MRI/PET/MEG)<br>• Data Entry<br>• Project Administration
Anne Labonté	Labonté, Anne	• McGill University, Montreal, QC, CA<br>◦ Douglas Mental Health University Institute Research Centre, Montreal, QC, CA<br>▪ StoP-Alzheimer Centre, Montreal, QC, CA	• BSc		• Genetic Analysis and Biochemical Assays
Robert Laforce	Laforce, Robert	• Université Laval, Quebec, QC, CA	• PhD<br>• MD		• Clinical Evaluation<br>• Consultant
Marc Lalancette	Lalancette, Marc	• McGill University, Montreal, QC, CA<br>◦ Montreal Neurological Institute and Hospital, Montreal, QC, CA	• MSc		• Acquisition (MRI/PET/MEG)

Jean-Charles Lambert	Lambert, Jean-Charles	• Université de Lille, Lille, HDF, FR	• PhD		• Genetic Analysis and Biochemical Assays
Corina Lazarenco	Lazarenco, Corina	• McGill University, Montreal, QC, CA	• BA		• Data Entry
Jeannie-Marie Leoutsakos	Leoutsakos, Jeannie-Marie	• Johns Hopkins University, Baltimore, MD, USA	• PhD		• Consultant<br>• Data Analysis
Julia Loncke	Loncke, Julia	• McGill University, Montreal, QC, CA	• BSc		• Data Analysis<br>• Genetic Analysis and Biochemical Assays
Laurence Maligne Bruneau	Maligne Bruneau, Laurence	• McGill University, Montreal, QC, CA<br>◦ Douglas Mental Health University Institute Research Centre, Montreal, QC, CA<br>▪ StoP-Alzheimer Centre, Montreal, QC, CA			• Clinical Evaluation<br>• Data Entry<br>• Genetic Analysis and Biochemical Assays<br>• Interview Data Collection<br>• LP/CSF Collection
Amelie Metz	Metz, Amelie	• McGill University, Montreal, QC, CA	• MSc		• Data Analysis<br>• Processing and Evaluation (MRI/PET/MEG)
Bratislav Misic	Misic, Bratislav	• McGill University, Montreal, QC, CA<br>◦ Montreal Neurological Institute and Hospital, Montreal, QC, CA	• PhD		• Data Analysis

Bery Mohammediyan	Mohammediyan, Bery	• McGill University, Montreal, QC, CA<br>◦ Douglas Mental Health University Institute Research Centre, Montreal, QC, CA	• BSc		• Acquisition (MRI/PET/MEG)
Eugenia Nita Capota	Nita Capota, Eugenia	• McGill University, Montreal, QC, CA<br>◦ Douglas Mental Health University Institute Research Centre, Montreal, QC, CA	• BSc		• Clinical Evaluation<br>• Interview Data Collection<br>• LP/CSF Collection
Alix Noly-Gandon	Noly-Gandon, Alix	• McGill University, Montreal, QC, CA	• PhD		• Interview Data Collection
Adrian Eduardo Noriega de la Colina	Noriega de la Colina, Adrian	• McGill University, Montreal, QC, CA<br>◦ Montreal Neurological Institute and Hospital, Montreal, QC, CA	• PhD<br>• MD		• Interview Data Collection
Pierre Orban	Orban, Pierre	• Université de Montréal, Montreal, QC, CA<br>◦ Centre de recherche Institut Universitaire de Gériatrie de Montréal, Montreal, QC, CA<br>• McGill University, Montreal, QC, CA<br>◦ Douglas Mental Health University Institute Research Centre, Montreal, QC, CA<br>▪ StoP-Alzheimer Centre, Montreal, QC, CA	• PhD		• Data Analysis<br>• Processing and Evaluation (MRI/PET/MEG)
Valentin Ourry	Ourry, Valentin	• McGill University, Montreal, QC, CA<br>◦ Douglas Mental Health University Institute Research Centre, Montreal, QC, CA	• PhD		• Data Analysis
Cynthia Picard	Picard, Cynthia	• McGill University, Montreal, QC, CA<br>◦ Douglas Mental Health University Institute Research Centre, Montreal, QC, CA<br>▪ StoP-Alzheimer Centre, Montreal, QC, CA	• PhD		• Genetic Analysis and Biochemical Assays

Alexa Pichet Binette	Pichet Binette, Alexa	• McGill University, Montreal, QC, CA<br>◦ Douglas Mental Health University Institute Research Centre, Montreal, QC, CA<br>▪ StoP-Alzheimer Centre, Montreal, QC, CA	• PhD		• Cognitive Evaluation<br>• Data Analysis<br>• Interview Data Collection<br>• Processing and Evaluation (MRI/PET/MEG)
Nathalie Prenevost	Prenevost, Nathalie	• McGill University, Montreal, QC, CA<br>◦ Montreal Neurological Institute and Hospital, Montreal, QC, CA	• BSc		• Acquisition (MRI/PET/MEG)<br>• Cognitive Evaluation<br>• Data Entry<br>• Interview Data Collection
Ting Qiu	Qiu, Ting	• McGill University, Montreal, QC, CA	• MSc		• Data Analysis
Marc James Quesnel	Quesnel, Marc	• McGill University, Montreal, QC, CA<br>◦ Douglas Mental Health University Institute Research Centre, Montreal, QC, CA	• MSc		• Data Analysis<br>• Genetic Analysis and Biochemical Assays
Charles Ramassamy	Ramassamy, Charles	• Institut national de la recherche scientifique, Laval, QC, CA	• PhD		• Genetic Analysis and Biochemical Assays
Jean-Michel Raoult	Raoult, Jean-Michel	• McGill University, Montreal, QC, CA<br>◦ Douglas Mental Health University Institute Research Centre, Montreal, QC, CA<br>▪ StoP-Alzheimer Centre, Montreal, QC, CA	• BSc		• Database Management<br>• Database Programming

Jordana Remz	Remz, Jordana	• McGill University, Montreal, QC, CA<br>◦ Douglas Mental Health University Institute Research Centre, Montreal, QC, CA	• BSc		• Data Analysis<br>• Database Programming<br>• Processing and Evaluation (MRI/PET/MEG)
Erica Rothman	Rothman, Erica	• McGill University, Montreal, QC, CA			• Data Entry
Safa Sanami	Sanami, Safa	• Concordia University, Montreal, QC, CA	• PhD		• Data Analysis<br>• Processing and Evaluation (MRI/PET/MEG)
Isabel Sarty	Sarty, Isabel	• McGill University, Montreal, QC, CA<br>◦ Douglas Mental Health University Institute Research Centre, Montreal, QC, CA	• MSc		• Data Analysis<br>• Genetic Analysis and Biochemical Assays
Elisabeth Sylvain	Sylvain, Elisabeth	• McGill University, Montreal, QC, CA<br>◦ Douglas Mental Health University Institute Research Centre, Montreal, QC, CA<br>▪ StoP-Alzheimer Centre, Montreal, QC, CA	• BA		• Acquisition (MRI/PET/MEG)<br>• Cognitive Evaluation<br>• Data Entry<br>• Interview Data Collection
Andras Tikasz	Tikasz, Andras	• McGill University, Montreal, QC, CA	• PhD		• Data Analysis
Stefanie Tremblay	Tremblay, Stefanie	• Concordia University, Montreal, QC, CA	• MSc		• Data Analysis

Jennifer Tremblay-Mercier	Tremblay-Mercier, Jennifer	• McGill University, Montreal, QC, CA<br>◦ Douglas Mental Health University Institute Research Centre, Montreal, QC, CA<br>▪ StoP-Alzheimer Centre, Montreal, QC, CA	• MSc		• Data Analysis<br>• Project Administration<br>• Randomization and Pharmacy Allocation
Stephanie Tullo	Tullo, Stephanie	• McGill University, Montreal, QC, CA<br>◦ Douglas Mental Health University Institute Research Centre, Montreal, QC, CA	• BSc		• Data Analysis<br>• Processing and Evaluation (MRI/PET/MEG)
Jacob Turcotte	Turcotte, Jacob	• McGill University, Montreal, QC, CA	• BSc		• Data Analysis
Irem Ulku	Ulku, Irem	• McGill University, Montreal, QC, CA	• PhD		• Data Analysis
Paolo Vitali	Vitali, Paolo	• McGill University, Montreal, QC, CA<br>◦ Douglas Mental Health University Institute Research Centre, Montreal, QC, CA<br>▪ McGill University Research Centre for Studies in Aging, Montreal, QC, CA<br>◦ Montreal Neurological Institute and Hospital, Montreal, QC, CA	• PhD<br>• MD		• Consultant
Ellen Wang	Wang, Ellen	• McGill University, Montreal, QC, CA	• MSc		• Processing and Evaluation (MRI/PET/MEG)
Alfie Wearn	Wearn, Alfie	• McGill University, Montreal, QC, CA	• PhD		• Data Analysis<br>• Processing and Evaluation (MRI/PET/MEG)
Kayla Williams	Williams, Kayla	• McGill University, Montreal, QC, CA	• BA		• Data Analysis<br>• Data Entry

Yara Yakoub	Yakoub, Yara	• McGill University, Montreal, QC, CA<br>◦ Douglas Mental Health University Institute Research Centre, Montreal, QC, CA	• MSc		• Data Analysis<br>• Data Entry<br>• Processing and Evaluation (MRI/PET/MEG)
Robert Zatorre	Zatorre, Robert	• McGill University, Montreal, QC, CA<br>◦ Montreal Neurological Institute and Hospital, Montreal, QC, CA	• PhD		• Consultant
Henrik Zetterberg	Zetterberg, Henrik	• University of Gothenburg, Gothenburg, SE-O, SE	• PhD		• Genetic Analysis and Biochemical Assays
