# Supplementary material for: Plasma p-tau217 and tau-PET predict future cognitive decline among cognitively unimpaired individuals: implications for clinical trials
Source: Nat Aging. 2025 Mar 28;5(5):883–96. doi: 10.1038/s43587-025-00835-z (PMC12092243; doi:10.1038/s43587-025-00835-z)
Supplement: Supplementary file 2 — Reporting Summary [file 43587_2025_835_MOESM2_ESM.pdf]

Corresponding author(s): Rik Ossenkoppele
Oskar Hansson

Last updated by author(s): Jan 15, 2025

Reporting Summary

Nature Portfolio wishes to improve the reproducibility of the work that we publish. This form provides structure for consistency and transparency in reporting. For further information on Nature Portfolio policies, see our Editorial Policies and the Editorial Policy Checklist.

Statistics

For all statistical analyses, confirm that the following items are present in the figure legend, table legend, main text, or Methods section.

n/a	Confirmed	
☐	☒	The exact sample size (n) for each experimental group/condition, given as a discrete number and unit of measurement
☐	☒	A statement on whether measurements were taken from distinct samples or whether the same sample was measured repeatedly
☐	☒	The statistical test(s) used AND whether they are one- or two-sided<br>*Only common tests should be described solely by name; describe more complex techniques in the Methods section.*
☐	☒	A description of all covariates tested
☐	☒	A description of any assumptions or corrections, such as tests of normality and adjustment for multiple comparisons
☐	☒	A full description of the statistical parameters including central tendency (e.g. means) or other basic estimates (e.g. regression coefficient) AND variation (e.g. standard deviation) or associated estimates of uncertainty (e.g. confidence intervals)
☐	☒	For null hypothesis testing, the test statistic (e.g. F, t, r) with confidence intervals, effect sizes, degrees of freedom and P value noted<br>*Give P values as exact values whenever suitable.*
☒	☐	For Bayesian analysis, information on the choice of priors and Markov chain Monte Carlo settings
☒	☐	For hierarchical and complex designs, identification of the appropriate level for tests and full reporting of outcomes
☐	☒	Estimates of effect sizes (e.g. Cohen's d, Pearson's r), indicating how they were calculated

Our web collection on statistics for biologists contains articles on many of the points above.

Software and code

Policy information about availability of computer code

Data collection	No specific code was used in data collection, but the custom code can be requested through the corresponding authors
Data analysis	R (v4.1.0) was used for statistical analyses as described in the manuscript. The following packages were used: rsq (v2.6), longpower (v1.0.24), powerSurvEpi (v0.1.3) and boot (v1.3-28.1).

For manuscripts utilizing custom algorithms or software that are central to the research but not yet described in published literature, software must be made available to editors and reviewers. We strongly encourage code deposition in a community repository (e.g. GitHub). See the Nature Portfolio guidelines for submitting code & software for further information.

Data

Policy information about availability of data

All manuscripts must include a data availability statement. This statement should provide the following information, where applicable:

- Accession codes, unique identifiers, or web links for publicly available datasets
- A description of any restrictions on data availability
- For clinical datasets or third party data, please ensure that the statement adheres to our policy

Due to the multicenter design of the study, access to individual participant data from each cohort will have to be made available through the PIs of the respective

cohorts. Generally, anonymised data can be shared by request from qualified academic investigators for the purpose of replicating procedures and results presented in the article, if data transfer is in agreement with the data protection regulation at the institution and is approved by the local Ethics Review Board.

Research involving human participants, their data, or biological material

Policy information about studies with human participants or human data. See also policy information about sex, gender (identity/presentation), and sexual orientation and race, ethnicity and racism.

Reporting on sex and gender	Sex is reported in Table 1, based on self-reporting information. No sex-specific analyses were conducted.
Reporting on race, ethnicity, or other socially relevant groupings	Race and ethnicity were not available in all cohorts, and thus, not shown. Other socially relevant data such as education level is shown in Table 1.
Population characteristics	Sample characteristics are shown in Table 1
Recruitment	Subjects were included from prospective cohort studies in Australia, Canada, Netherlands, Sweden, and the United States. Subject recruitment protocols for each cohort study are provided in the supplementary material.
Ethics oversight	Written informed consent was obtained from all participants and local institutional review boards for human research approved the study. This includes the Mayo Clinic and Olmsted Medical Center institutional review boards for MSCA, regional ethics committee at Lund University for BioFINDER-1 and BioFINDER-2, institutional human research ethics committees of Austin Health, St. Vincent's Health, Hollywood Private Hospital and Edith Cowan University for AIBL, the medical ethics review committee of the Amsterdam University Medical Center for ADC, the McGill Research Ethics Board for PREVENT-AD and TRIAD, the Washington University School of Medicine in St. Louis for Knight ADRC and the University of Wisconsin Institutional Review Board for WRAP.

Note that full information on the approval of the study protocol must also be provided in the manuscript.

Field-specific reporting

Please select the one below that is the best fit for your research. If you are not sure, read the appropriate sections before making your selection.

☒ Life sciences ☐ Behavioural & social sciences ☐ Ecological, evolutionary & environmental sciences

For a reference copy of the document with all sections, see nature.com/documents/nr-reporting-summary-flat.pdf

Life sciences study design

All studies must disclose on these points even when the disclosure is negative.

Sample size	We included a total of 1,474 participants from 9 independent cohorts. Sample size was not determined a priori and was instead determined based on availability of data.
Data exclusions	There was no data exclusion in this study.
Replication	Results of this study were consistent across 9 observational cohorts. Results by individual cohorts are shown in Supplementary material and extended data.
Randomization	This is an obervational diagnostic study and no allocation into groups was performed. Hence randomization is not relevant to this study.
Blinding	Plasma measurments were performed blinded to the clinical data.

Reporting for specific materials, systems and methods

We require information from authors about some types of materials, experimental systems and methods used in many studies. Here, indicate whether each material, system or method listed is relevant to your study. If you are not sure if a list item applies to your research, read the appropriate section before selecting a response.

Materials & experimental systems

n/a	Involved in the study	
☐	☒	Antibodies
☒	☐	Eukaryotic cell lines
☒	☐	Palaeontology and archaeology
☒	☐	Animals and other organisms
☐	☒	Clinical data
☒	☐	Dual use research of concern
☒	☐	Plants

Methods

n/a	Involved in the study	
☒	☐	ChIP-seq
☒	☐	Flow cytometry
☒	☐	MRI-based neuroimaging

Antibodies

Antibodies used	Detailed information of the immunoassays in the manuscript has been published previously (and is referred to in the manuscript).
Validation	The plasma p-tau217 Lilly immunoassay used in BioFINDER-1, BioFINDER-2, ADC, WRAP, PREVENT-AD, MCSA and Knight ADRC has been previously validated and described in full detail by Palmqvist et al. (JAMA. 2020:324(8):772-781) and by Groot et al (Alz Res Ther; 2022 May 14;14(1):67). For AIBL and TRIAD a plasma p-tau217+ assay developed by Janssen R&D (CA, USA) on a Single Molecule Array (Simoa) HD-X platform was used, which was previously validated (JPAD; 2023; 10:828-836)

Clinical data

Policy information about clinical studies

All manuscripts should comply with the ICMJE guidelines for publication of clinical research and a completed CONSORT checklist must be included with all submissions.

Clinical trial registration	NCT03174938 (BioFINDER-2) and NCT01208675 (BioFINDER-1)
Study protocol	For BioFINDER-2: www.biofinder.se.
Data collection	All participants were i) cognitively unimpaired at baseline defined by neuropsychological test scores within the normative range given an individuals' age, sex and educational background, ii) had Amyloid-PET available to determine Ab status, iii) underwent a Tau-PET scan and blood sampling within a maximum 1 year interval, and iv) had at least one clinical follow-up visit available. Follow-up data was collected until November 1st, 2023.
Outcomes	Main outcomes were cognitive decline, as measured by change in mPACC, and progression to MCI.

Plants

Seed stocks	NA
Novel plant genotypes	NA
Authentication	NA
